# Author Correction: Major restructuring of marine plankton assemblages under global warming

**DOI:** 10.1038/s41467-021-26564-6

**Published:** 2021-10-26

**Authors:** Fabio Benedetti, Meike Vogt, Urs Hofmann Elizondo, Damiano Righetti, Niklaus E. Zimmermann, Nicolas Gruber

**Affiliations:** 1 Environmental Physics, Institute of Biogeochemistry and Pollutant Dynamics, ETH Zürich, Zürich, Switzerland; 2 Dynamic Macroecology, Swiss Federal Research Institute WSL, Birmensdorf, Switzerland; 3 Department of Environmental Systems Science, ETH Zurich, Zürich, Switzerland

**Keywords:** Climate-change ecology, Macroecology, Marine biology

Correction to: *Nature Communications* 10.1038/s41467-021-25385-x, published online 1 September 2021.

In the Acknowledgements section, the funding source ‘F.B. received support from ETH Zürich and from the European Union’s Horizon 2020 research and innovation program under grant agreement no. SEP-210591007’ should have read ‘F.B. received support from ETH Zürich. This project has received funding from the European Union’s Horizon 2020 research and innovation programme under grant agreement no. 862923. This output reflects only the author’s view, and the European Union cannot be held responsible for any use that may be made of the information contained therein.’. The original article has been corrected.

