# Supplementary material for: Major restructuring of marine plankton assemblages under global warming
Source: Nat Commun. 2021 Sep 1;12:5226. doi: 10.1038/s41467-021-25385-x (PMC8410869; doi:10.1038/s41467-021-25385-x)
Supplement: Supplementary file 1 — Supplementary Information [file 41467_2021_25385_MOESM1_ESM.pdf]

Nature Communications

Supplementary Information file for

Major restructuring of marine plankton assemblages under global warming

Fabio Benedetti[1*], Meike Vogt[1], Urs Hofmann Elizondo[1], Damiano Righetti[1], Niklaus E. Zimmermann[2,3], and Nicolas Gruber[1]

[1]Environmental Physics, Institute of Biogeochemistry and Pollutant Dynamics, ETH Zürich, 8092 Zürich, Switzerland. [2]Dynamic Macroecology, Swiss Federal Research Institute WSL, 8903 Birmensdorf, Switzerland. [3]Department of Environmental Systems Science, ETH Zurich, 8092 Zürich, Switzerland.

*Corresponding author: fabio.benedetti@usys.ethz.ch

Supplementary Figure 1

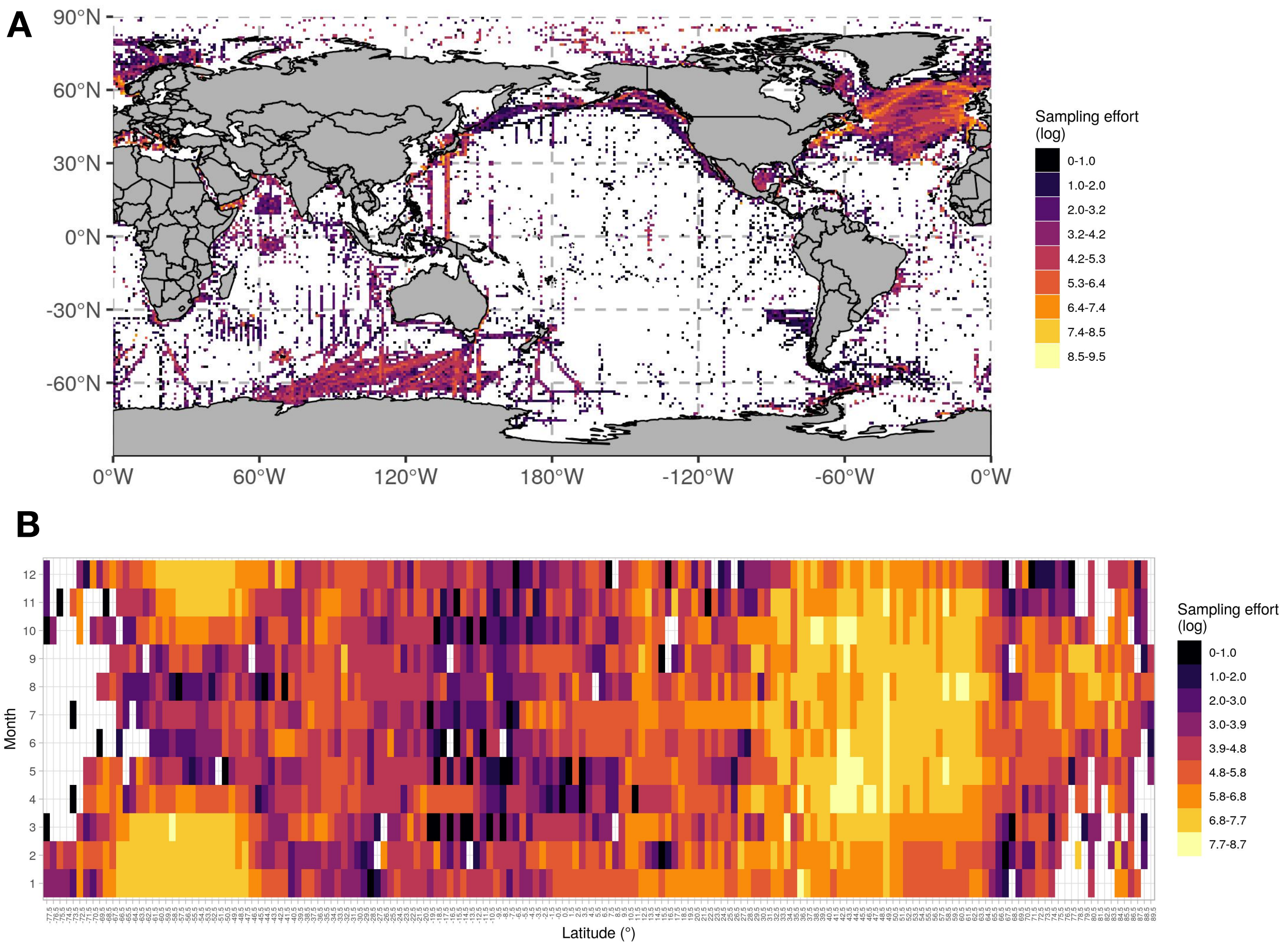

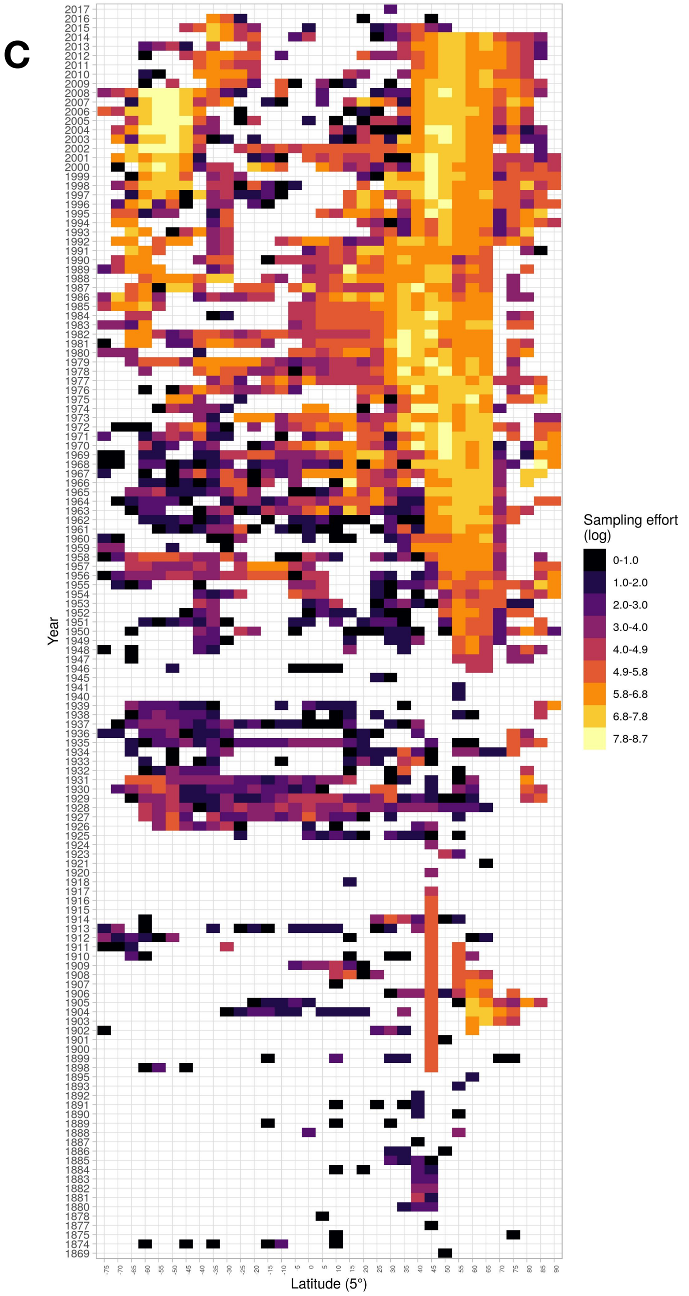

Supplementary Figure 1: Spatial and temporal distribution of sampling effort (i.e. total number of occurrences) in the zooplankton occurrence dataset per A) 1°x1° grid cells, B) 1° monthly grid cell (1 = January and 12 = December), and C) 5° yearly grid cells. Spatial and temporal distribution of sampling effort of the phytoplankton occurrence dataset have already been extensively described and analyzed by Righetti et al. [6,7].

Supplementary Figure 2

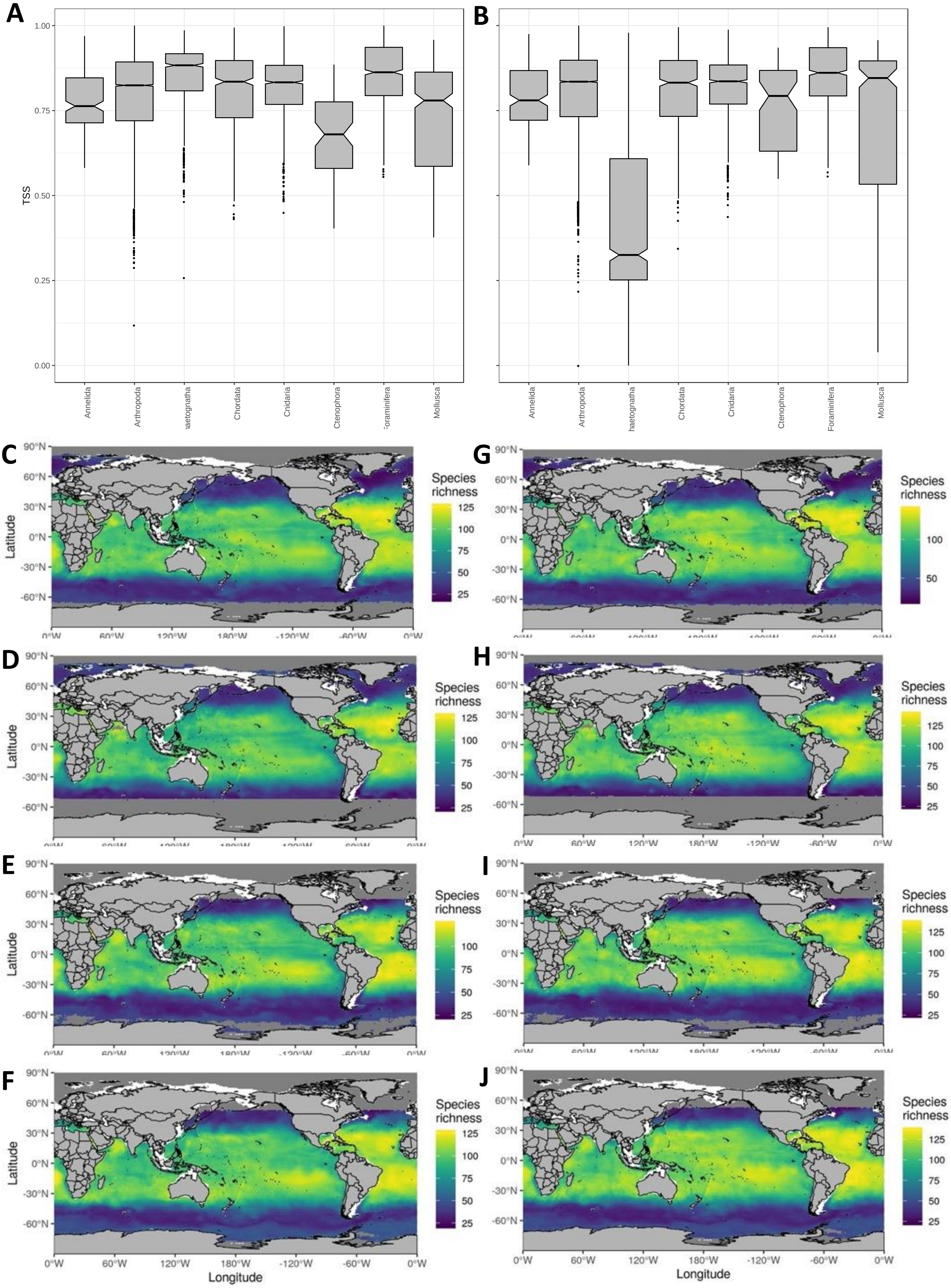

Supplementary Figure 2: Distribution of True Skill Statistic (TSS) for each zooplankton groups for the species distribution models (SDMs) based on (A) total-group background data, and (B) target-group background data and the resulting species richness patterns (sum of species habitat suitability indices) for (C) to (F) total-group background data, and (G) to (J) target-group background data. The maps (C) and (G) represent April species richness, (D) and (H) represent July species richness, (E) and (I) represent October species richness, and (F) and (J) represent January species richness. Only those species presenting at least 100 occurrences were modelled (n = 371). SDMs used the following preliminary set of predictors: SST, SSS, dSST, logChl, MLD, PAR, logSiOH4 and N*. The lower, middle and upper boundaries of the boxplots shown in (A) and (B) correspond to the 25th, 50th and 75th percentiles. The lower and upper whiskers extend from the hinges to the lowest or largest value no further than 1.5*IQR (inter-quartile range) from the lower and upper hinges. For the boxplots shown in (A) and (B), the sample size (N species modelled x 10 cross evaluation runs) is: N = 40 for Annelida, N = 2590 for Arthropoda, N = 250 for Chaetognatha, N = 110 for Chordata, N = 370 for Cnidaria, N = 20 for Ctenophora, N = 230 for Foraminifera, and N = 100 for Mollusca.

Supplementary Figure 3: Distribution of (A) True Skill Statistics (TSS) and (B) Area Under the Curve (AUC) for all phytoplankton (N = 348 species modelled, in blue) and zooplankton species (N = 541 species modelled, in red), between each of the final four sets of environmental predictors. TSS and AUC are the two main indices used for evaluating and validating the species distributions models (SDMs) developed: Generalized Linear Models (GLM), Generalized Additive Models (GAM), Artificial Neural Networks (ANN) and classification Random Forest (RF). The lower, middle and upper boundaries of all the boxplots correspond to the 25th, 50th and 75th percentiles. The lower and upper whiskers extend from the hinges to the lowest or largest value no further than 1.5*IQR (inter-quartile range) from the lower and upper hinges. The sample size of each boxplot is N species modelled x 10 SDMs cross evaluation runs.

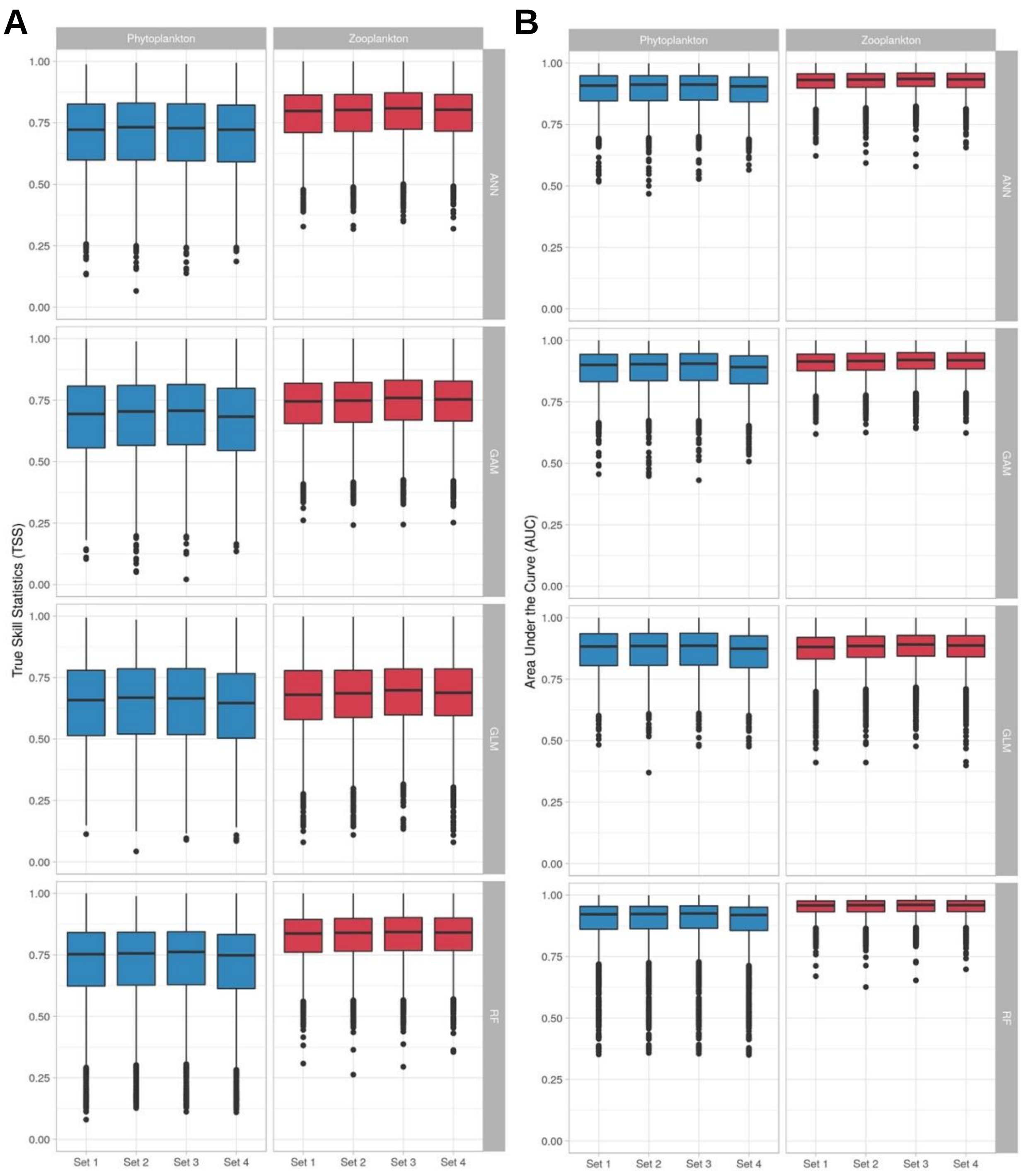

Supplementary Figure 4

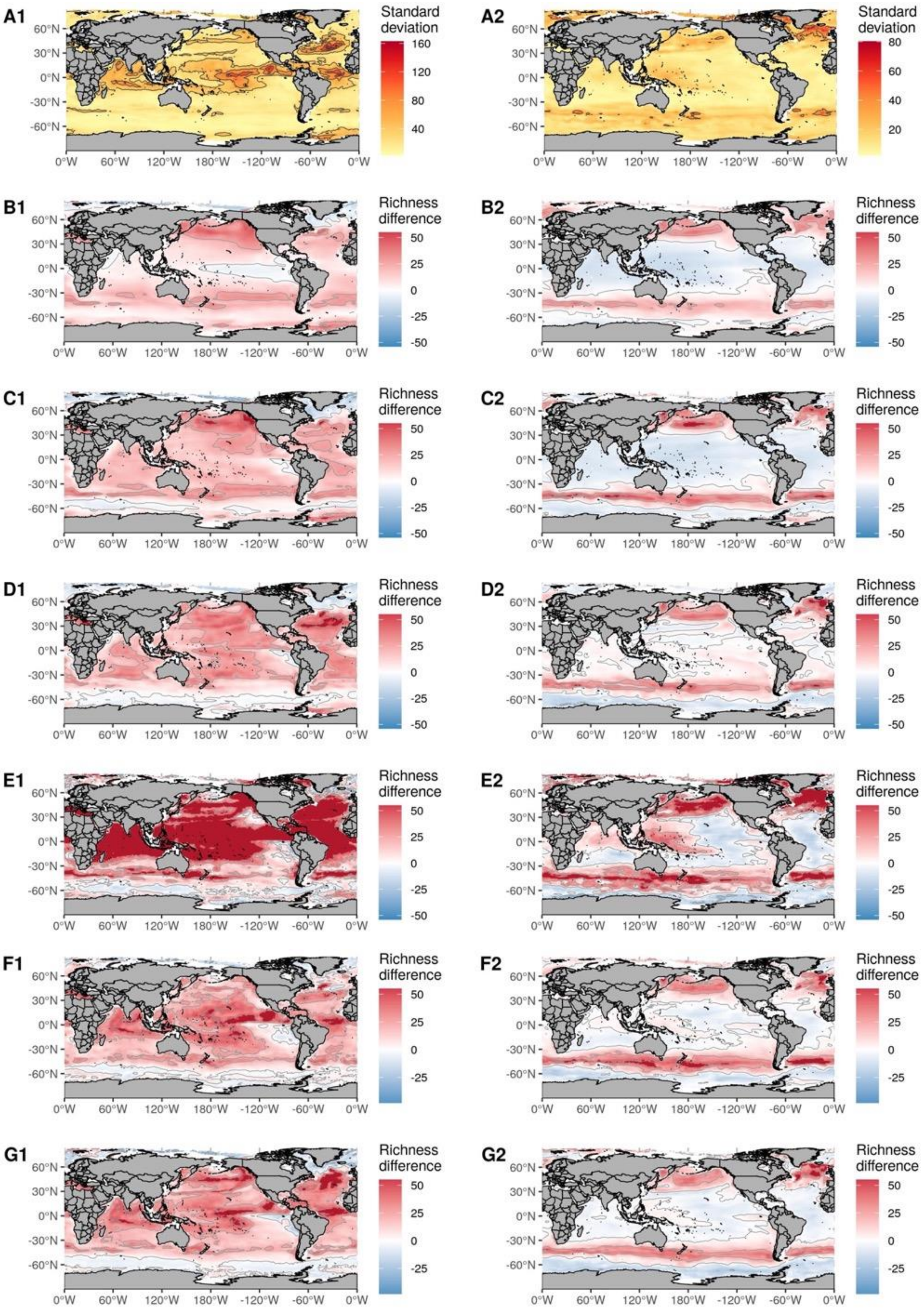

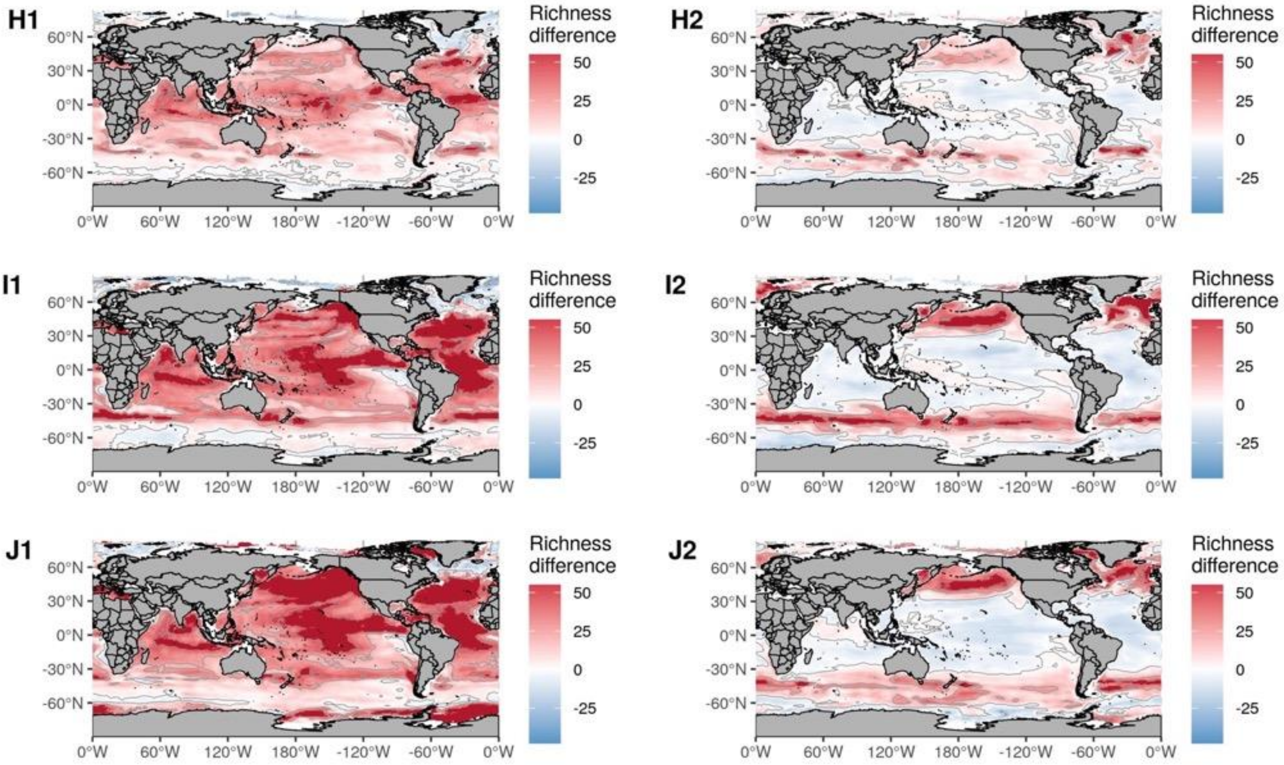

Supplementary Figure 4: Spatial distribution of (A) the uncertainty of our mean ensemble projections of (A1) phytoplankton and (A2) zooplankton changes in mean annual species richness as well as the ensemble projections of difference in mean annual species richness for every (B) to (E) species distribution model (B = GLM, C = GAM, D = ANN and E = RF) and every F-J) earth system model (F = CESM1, G = CNRM-CM5, H = GFDL-ESM2M, I = IPSL-CM5A-LR, J = MIROC5). Maps labeled with a “1” on the left hand side correspond to phytoplankton projections. Maps labeled with a “2” on the right hand side correspond to zooplankton projections. The amplitude and the spatial patterns of standard deviation indicate the level of uncertainties in our ensemble projections that is due to disagreement between the models used (SDMs and ESMS alike). Uncertainty is higher for phytoplankton species richness projections than for zooplankton richness, but, the main spatial features of %ΔSR are conserved across projections for both groups. Higher levels of uncertainty arise in regions where models disagree on the amplitude of the SR response to climate change because of their relative sensitivity.

Supplementary Figure 5

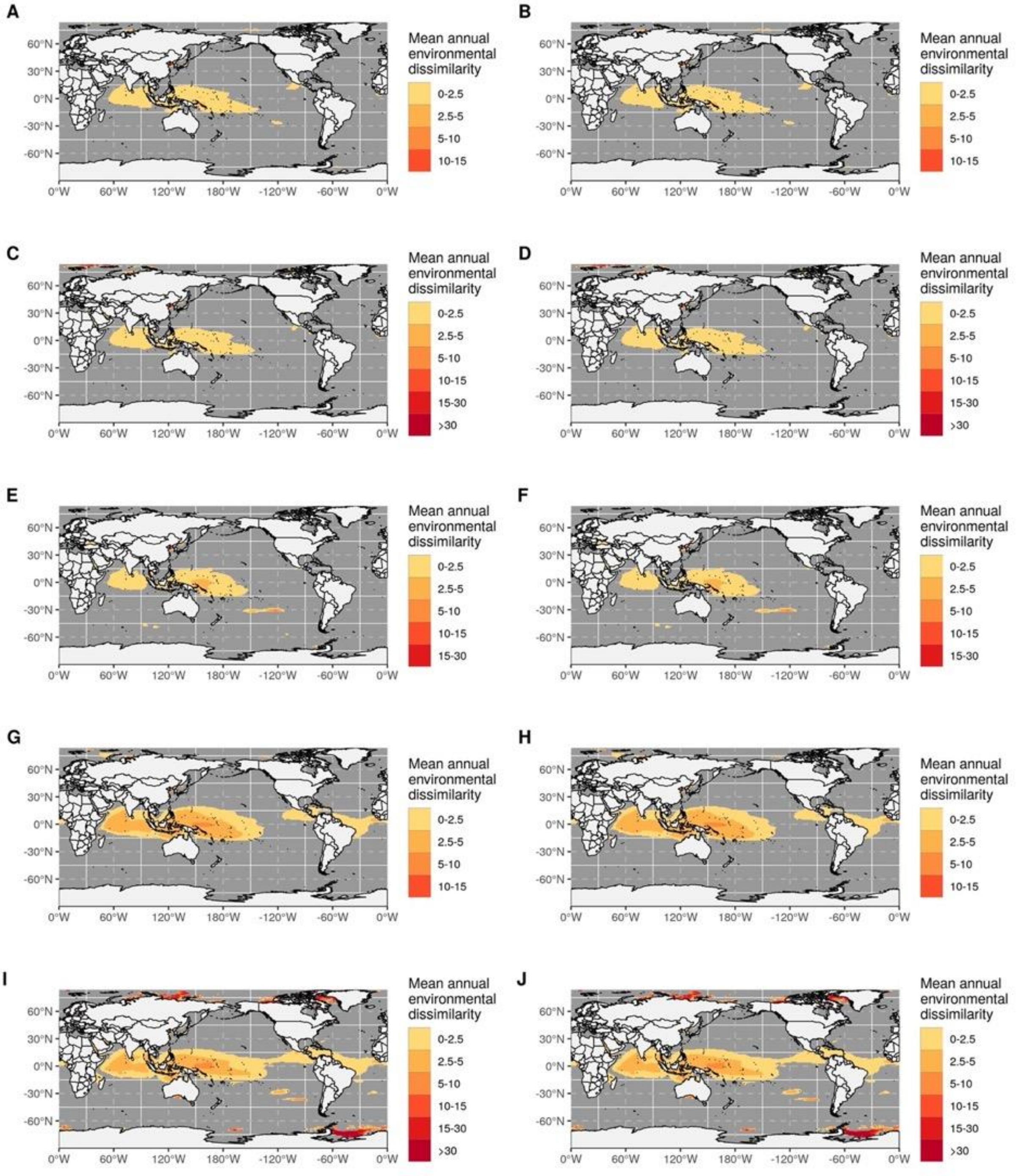

Supplementary Figure 5: Maps of mean annual environmental dissimilarity between the species environmental envelope of reference (i.e. environmental training set of the species distribution models) and the future environmental conditions estimated from the Multivariate Environmental Similarity Surface (MESS) algorithm for A,C,E,G,I) phytoplankton species and B,D,F,H,I) zooplankton species for every earth system model: (A)and (B) CESM; (C) and (D) CNRM-CM5; (E) and (F) GFDL-ESM2M; (G) and (H) IPSL- CM5A- LR; (I) and (J) MIROC5. The MESS were computed at species-level for every set of predictors and for every monthly ESM projection

(860x12x4x5=206400 MESS estimates). To summarize this information, mean annual MESS values were computed for each ESM separately and by distinguishing phyto- from zooplankton since their predictors set slightly differ (see Methods). Mean annual MESS estimates indicate those regions of the future ocean where non analog environmental conditions outside the species' reference envelope emerge on an annual scale.

Supplementary Figure 6

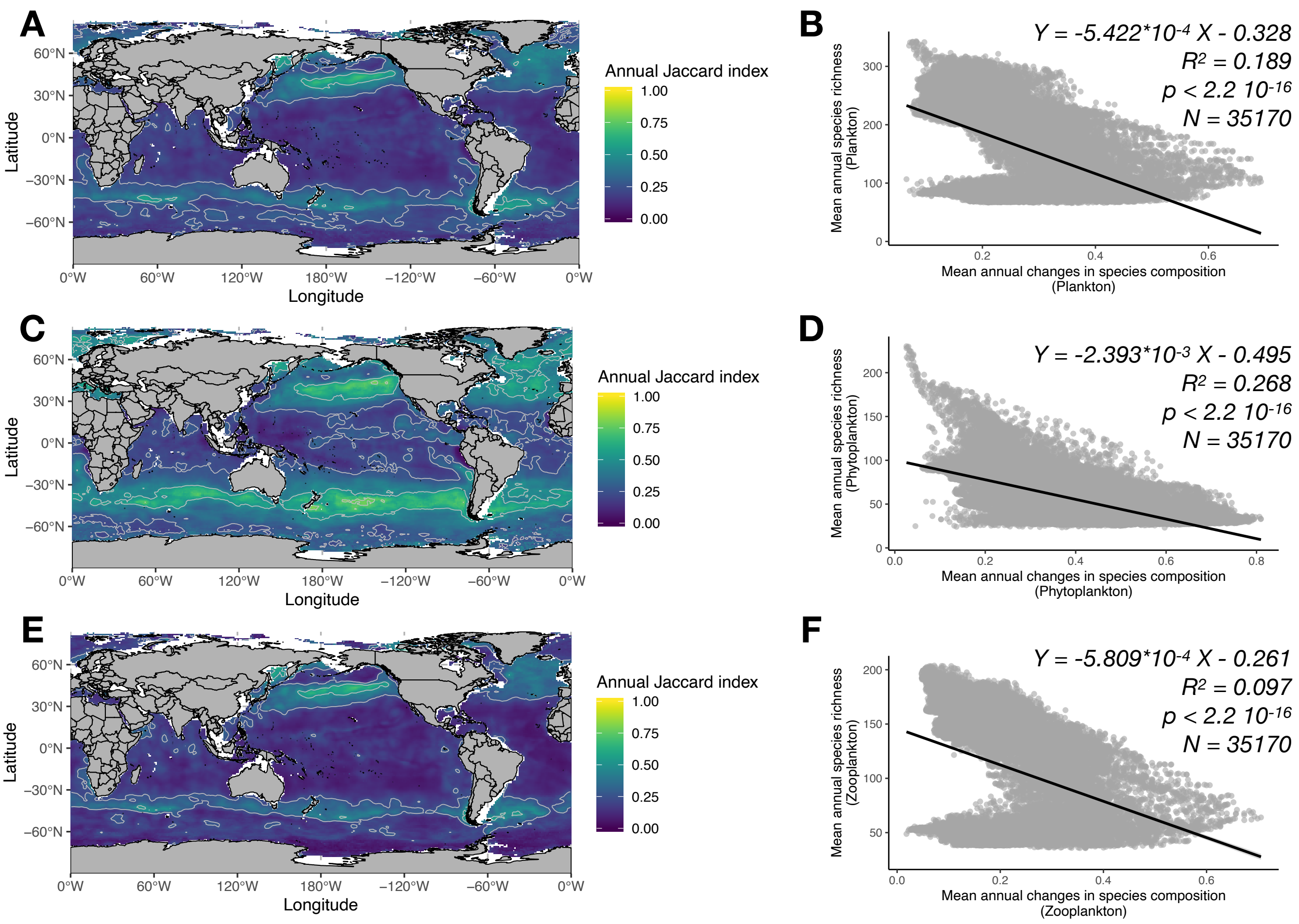

Supplementary Figure 6: Spatial distribution of (A)-(C)-(E) mean annual changes in month-to-month species composition and (B)-(D)-(F) its relationship to mean annual species richness for (A) and (B) all plankton, (C) and (D) phytoplankton, and (E) and (F) zooplankton. Month-to-month changes in composition were estimated through the Jaccard dissimilarity index and its two additive components (i.e. turn-over and nestedness). Jaccard's dissimilarity index is quantitative and scales between 0 and 1, with a value of 1 indicating a full re-shuffling in community composition. Lower Jaccard index values thus indicate the regions where month-to-month changes in species composition are weaker throughout the year. Contour lines indicate the 0.25 isopleth in mean annual Jaccard dissimilarity index. Ordinary linear regressions were used in (B)-(D)-(F) to test the strength of the linear relationships between mean annual species richness and month-to-month changes in species composition. The linear fits evidence the negative relationships observed between annual mean species richness and monthly changes in species composition. The plots indicate that the stronger seasonality in environmental conditions (and especially temperature) that prevails in temperate latitudes leads to a seasonal succession of species that prevents the establishment of a high species richness on the annual scale. Polar regions display both low mean annual species richness and low mean

annual changes in month-to-month composition since their annual species richness is limited by cold temperatures. Consequently, these regions depart from the overall trend of decreasing annual richness with higher mean month-to-month changes in composition. Fitting an ordinary linear regression without accounting for polar regions reinforces the explanatory power and the significance of the linear model. N = 35170 grid cells for all maps and ordinary linear regressions.

Supplementary Note 1: Analyzing the skills of species distribution models (SDMs) for various sets of environmental predictors of decreasing complexity (i.e. increasingly lower number of predictors), and justification of final choice of predictors set for modelling the phyto- and zooplankton species.

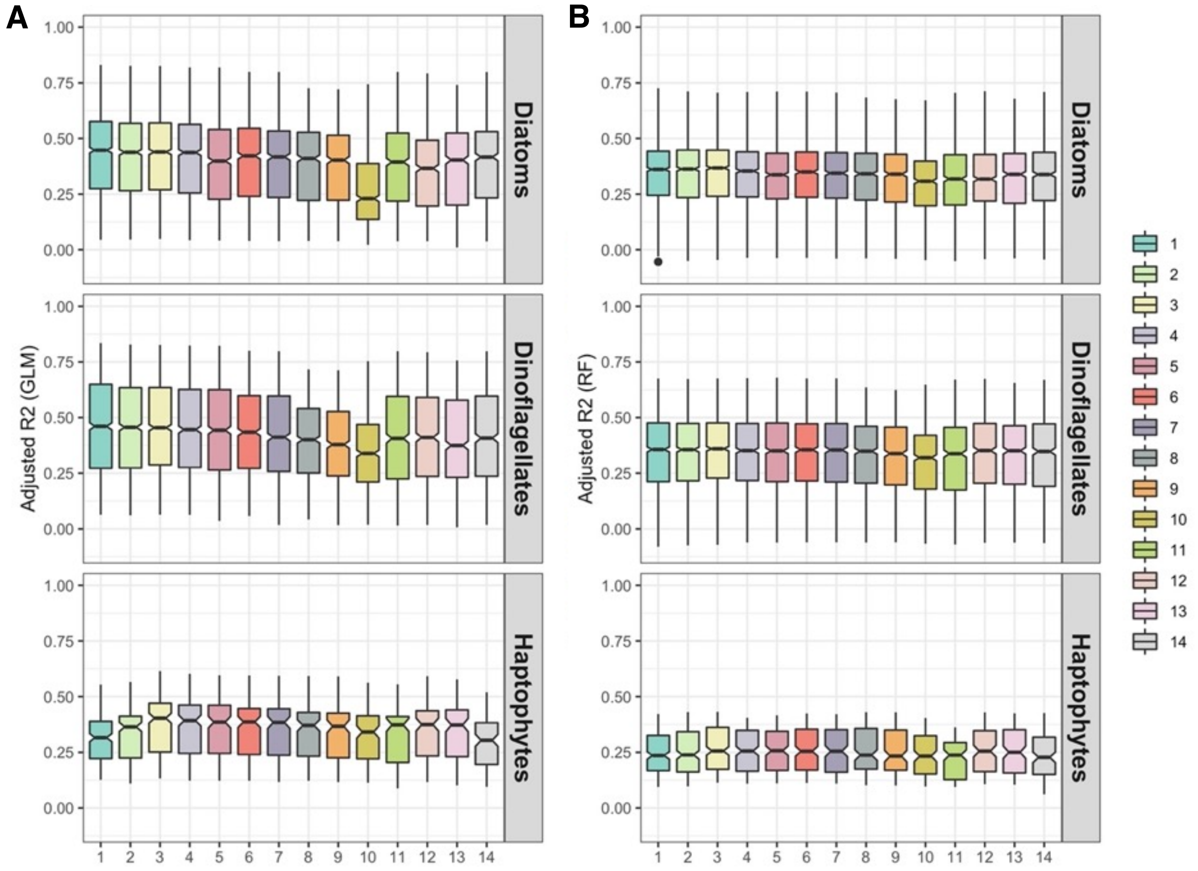

Supplementary Note 1-1: Distribution of the adjusted R^2 values of the models used to explore the explanatory power of 14 sets of predictors for predicting phytoplankton species distributions (presence/background data). Distributions are shown for the three main phytoplankton groups. Boxplots (A) display the interspecific variations in R^2 based on Generalized Linear Models (GLM), while (B) display the interspecific variations in R^2 for Random Forest (RF) models. The lower, middle and upper boundaries of the boxplots correspond to the 25th, 50th and 75th percentiles. The lower and upper whiskers extend from the hinges to the lowest or largest value no further than 1.5*IQR (inter-quartile range) from the lower and upper hinges. The sample size (N species modelled) of each boxplot is N = 163 for Diatoms, N = 157 for Dinoflagellates, N = 24 for Haptophytes.

The sets of predictors are as follows:

1. SST+dSST+logNO3+logChl+logSiOH4+logEKE+N*+Si*+Wind
2. SST+dSST+logNO3+logChl+logSiOH4+logEKE+N*+Si*+MLPAR
3. SST+dSST+logNO3+logChl+logSiOH4+logEKE+N*+Si*+MLD+PAR
4. SST+dSST+logNO3+logChl+logSiOH4+logEKE+N*+PAR
5. SST+dSST+logNO3+logChl+logEKE+N*+PAR
6. SST+dSST+logChl+logSiOH4+logEKE+N*+PAR
7. SST+dSST+logChl+logSiOH4+N*+PAR
8. SST+dSST+logChl+logSiOH4+logEKE+PAR

9. SST+dSST+logChl+logSiOH4+PAR
10. dSST+logChl+logSiOH4+N*+PAR
11. SST+logChl+logSiOH4+N*+PAR
12. SST+dSST+logChl+N*+PAR
13. SST+dSST+logSiOH4+N*+PAR
14. SST+dSST+logChl+logSiOH4+N*

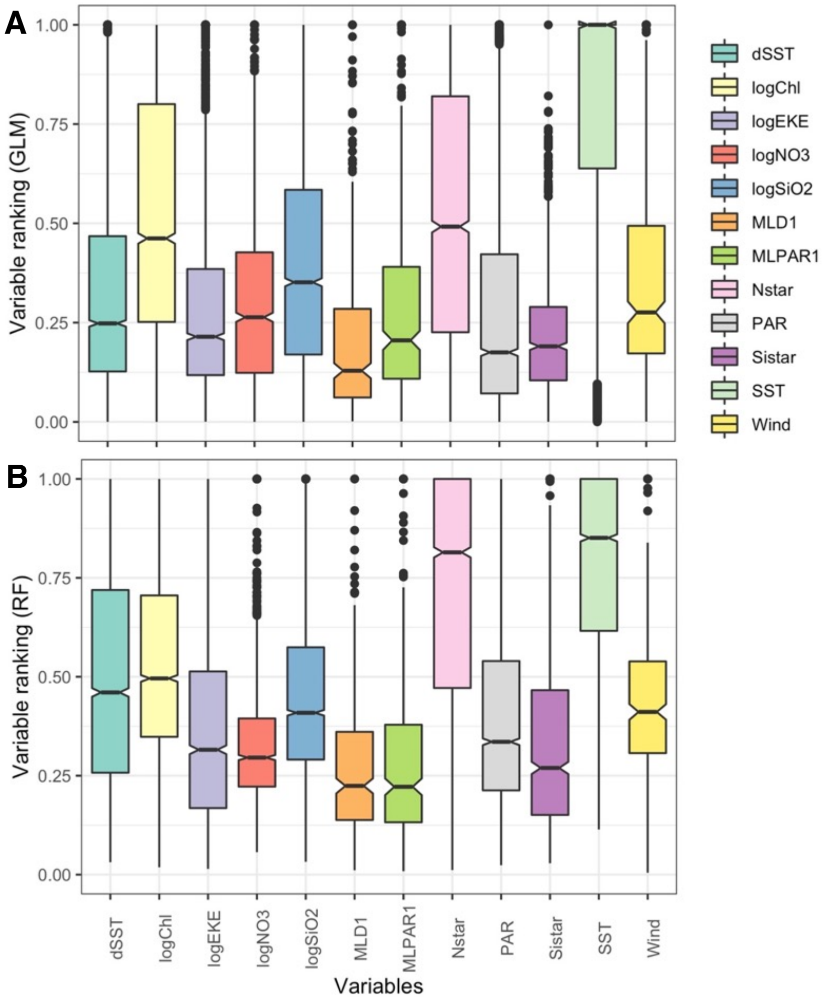

Supplementary Note 1-2: Distribution of the normalized relative importance rank of each environmental predictor across the 14 sets, for all phytoplankton species together, based on (A) Generalized Linear Models (GLM) and (B) Random Forest (RF) models. The lower, middle and upper boundaries of the boxplots correspond to the 25th, 50th and 75th percentiles. The lower and upper whiskers extend from the hinges to the lowest or largest value no further than 1.5*IQR (inter-quartile range) from the lower and upper hinges. For each boxplot, N = 4816 models (344 phytoplankton species x 14 predictors sets). Ranks were normalized so that a value of 1 indicates the most important variable for predicting a species' presence/ background dataset. For GLM, predictor importance was determined according to the absolute t-statistic. For RF, predictor importance was measured though the mean decrease in node impurity by summing over the number of splits (across all trees) that includes a variable, proportionally to the number of samples it splits. SST = Sea Surface Temperature; dSST = annual range of SST; logEKE = log of mean Eddy Kinetic Energy; MLD1 = Mixed-Layer Depth based on the temperature criterion; PAR

= Photosynthetic Active Radiation; MLPAR1 = PAR integrated over the MLD1; Wind = surface wind speed; logNO3 = log of nitrates surface concentration; logSiO2 = log of silicic acid surface concentration (same as logSiOH4); Nstar = excess of nitrates relative to phosphates based on the global Redfield ratio (N*); Sistar = excess of silicic acid relative to nitrates based on the global Redfield ratio (Si*); logChl = log of surface chlorophyll concentration.

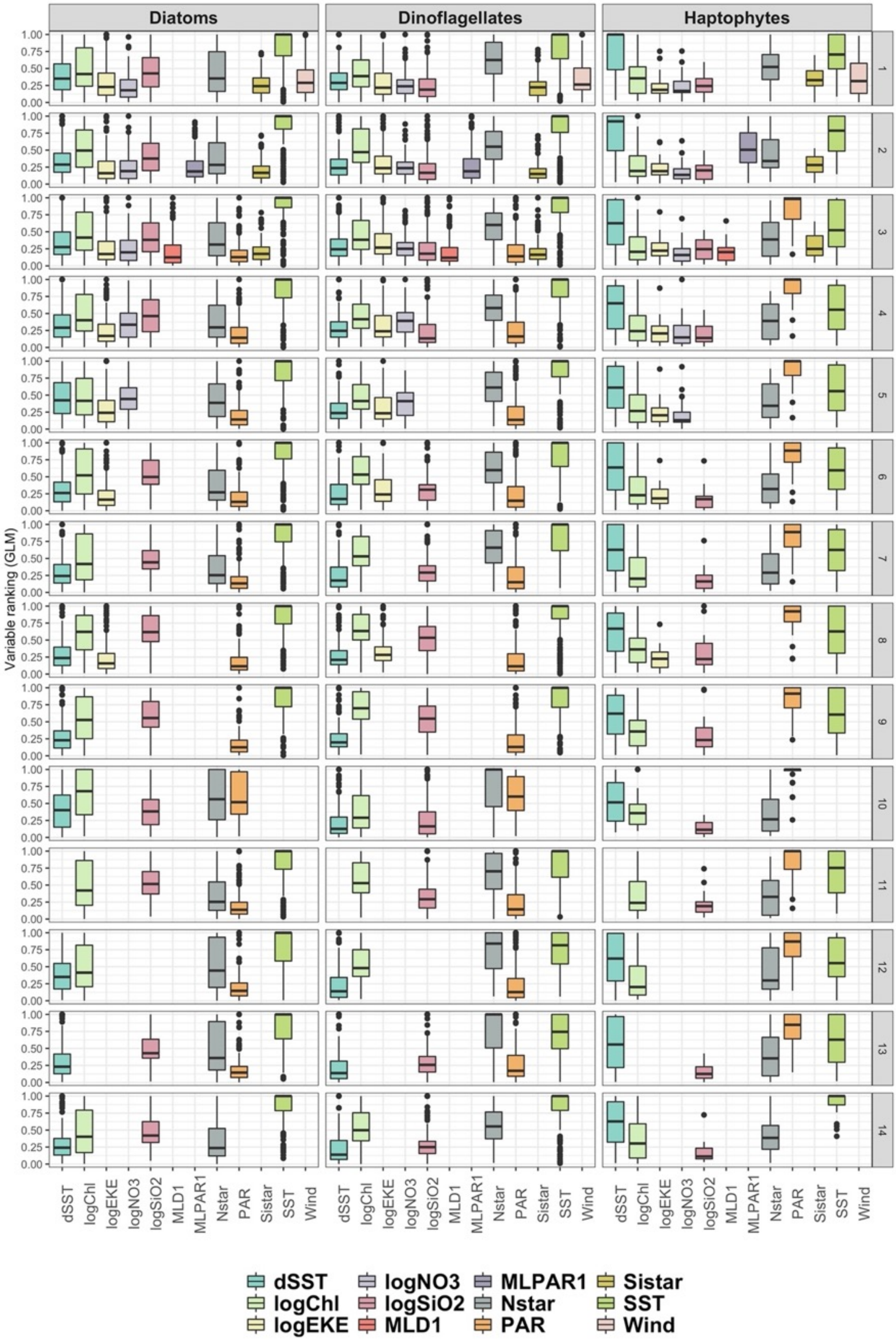

Supplementary Note 1-3: Distribution of the normalized ranks of relative importance of each environmental predictor across all of the 14 sets described above, for the three main phytoplankton groups based on Generalized Linear Models (GLM). The lower, middle and upper boundaries of the boxplots correspond to the 25th, 50th and 75th percentiles. The lower and upper whiskers extend from the hinges to the lowest or largest value no further than 1.5*IQR (inter-quartile range) from the lower and upper hinges. The total sample size is N = 4816 models (344 phytoplankton species x 14 predictors sets) and per group is: N = 2282 for Diatoms, N = 2198 for Dinoflagellates, and N = 336 for Haptophytes.

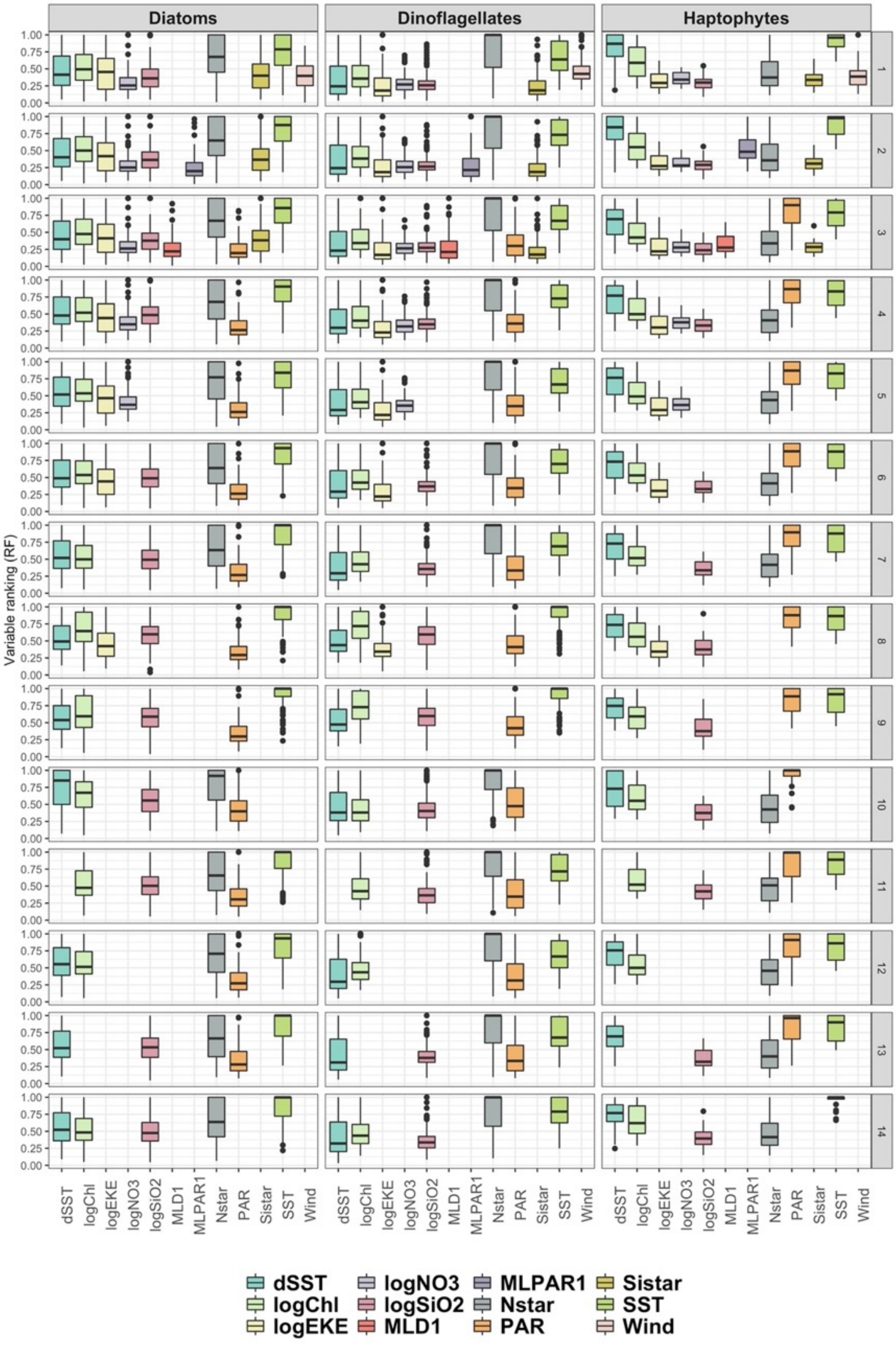

Supplementary Note 1-4: Distribution of the normalized ranks of relative importance of each environmental predictor across all of the 14 sets described above, for the three main phytoplankton groups based on Random Forest (RF) models. The lower, middle and upper boundaries of the boxplots correspond to the 25th, 50th and 75th percentiles. The lower and upper whiskers extend from the hinges to the lowest or largest value no further than 1.5*IQR (inter-quartile range) from the lower and upper hinges. The total sample size is N = 4816 models (344 phytoplankton species x 14 predictors sets) and per group is: N = 2282 for Diatoms, N = 2198 for Dinoflagellates, and N = 336 for Haptophytes.

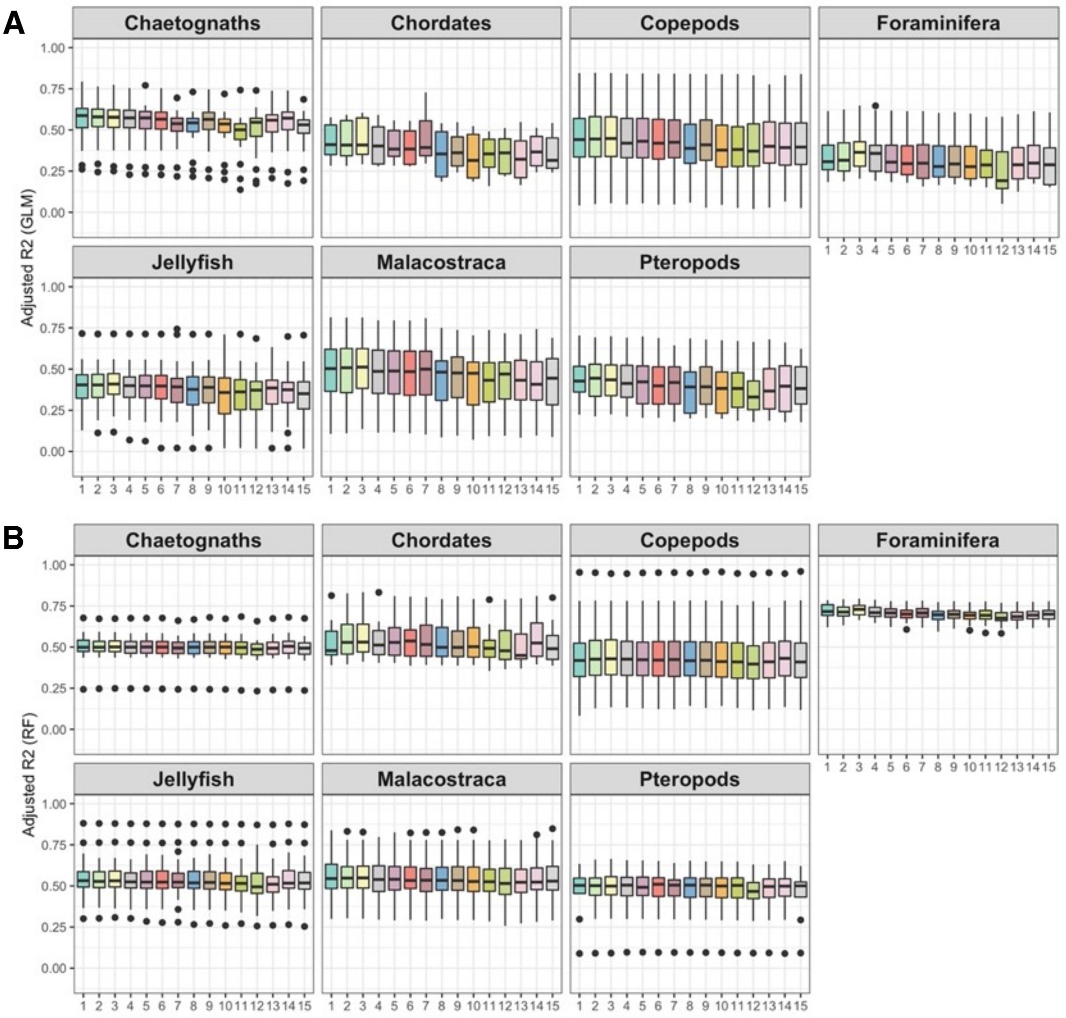

Supplementary Note 1-5: Distribution of the adjusted R^2 values of the models used to explore the explanatory power of 15 sets of predictors for predicting zooplankton species distributions (presence/background data). Distributions are shown for the seven main zooplankton groups. Boxplots (A) display the interspecific variations in R^2 based on Generalized Linear Models (GLM), while (B) display the interspecific variations in R^2 for Random Forest (RF) models. The lower, middle and upper boundaries of the boxplots correspond to the 25th, 50th and 75th percentiles. The lower and upper whiskers extend from the hinges to the lowest or largest value no further than 1.5*IQR (inter-quartile range) from the lower and upper hinges. The sample size (N species modelled) of each boxplot is N = 25 for Chaetognaths, N = 11 for Chordates, N = 209 for Copepods, N = 23 for Foraminifera, N = 39 for Jellyfish, N = 46 for Malacostraca, and N = 10 for Pteropods.

The sets of predictors are as follows:

1.SST+dSST+dO2+logChl+logSiOH4+logEKE+Wind+N*+Si*

2.SST+dSST+dO2+logChl+logSiOH4+logEKE+MLPAR+N*+Si*

3.SST+dSST+dO2+logChl+logSiOH4+logEKE+MLD+PAR+N*+Si*

4.SST+dSST+dO2+logChl+logSiOH4+logEKE+N*+PAR

5.SST+dSST+dO2+logChl+logSiOH4+logEKE+N*+MLD

6.SST+dSST+dO2+logChl+logSiOH4+logEKE+N*

7.SST+dSST+dO2+logChl+logNO3+logEKE+N*

8.SST+dSST+dO2+logChl+logSiOH4+N*

9.SST+dSST+dO2+logChl+logSiOH4+logEKE

10.SST+dSST+dO2+logChl+logSiOH4

11.dSST+dO2+logChl+logSiOH4+logEKE

12.SST+dO2+logChl+logSiOH4+logEKE

13.SST+dSST+logChl+logSiOH4+logEKE

14.SST+dSST+dO2+logSiOH4+logEKE

15.SST+dSST+dO2+logChl+logEKE

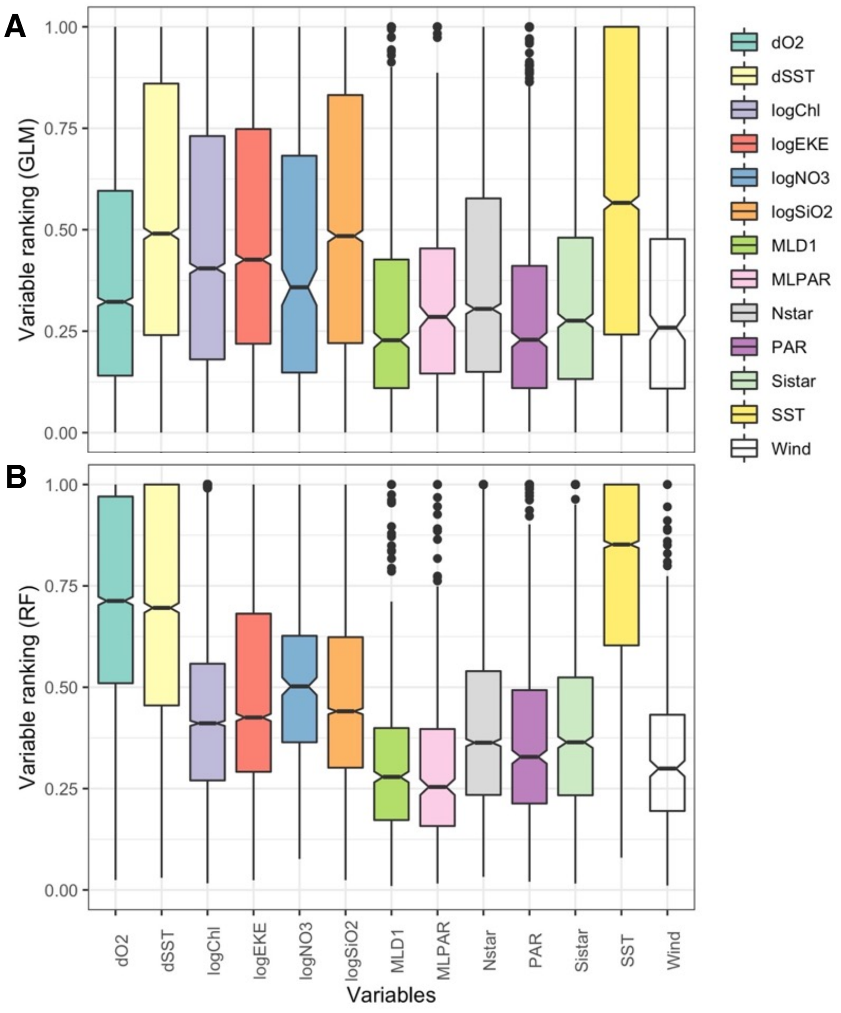

Supplementary Note 1-6: Distribution of the normalized ranks of relative importance of each environmental predictor across all of the 15 sets described above, for all zooplankton species together, based on (A) Generalized Linear Models (GLM) and (B) Random Forest (RF) models. The lower, middle and upper boundaries of the boxplots correspond to the 25th, 50th and 75th percentiles. The lower and upper whiskers extend from the hinges to the lowest or largest value no further than 1.5*IQR (inter-quartile range) from the lower and upper hinges. For each boxplot, N = 5565 models (344 zooplankton species x 15 predictors sets). Ranks were normalized so that a value of 1 indicates the most important variable for predicting a species' presence/background dataset. For GLM, predictor importance was determined according to the absolute t-statistic. For RF, predictor importance was measured though the mean decrease in node impurity by summing over the number of splits (across all trees) that includes a variable, proportionally to the number of samples it splits. SST = Sea Surface Temperature; dSST = annual range of SST; dO2 = dissolved oxygen concentration at 175m depth; logEKE = log of mean Eddy Kinetic Energy; MLD1 = Mixed-Layer Depth based on the temperature criterion; PAR = Photosynthetic Active Radiation; MLPAR1 = PAR integrated over the MLD1; Wind = surface wind speed; logNO3 = log of nitrates surface concentration; logSiO2 = log of silicic acid surface concentration (same as logSiOH4); Nstar = excess of nitrates relative to phosphates based on the global Redfield ratio (N*); Sistar = excess of silicic acid relative to nitrates based on the global Redfield ratio (Si*); logChl = log of surface chlorophyll concentration.

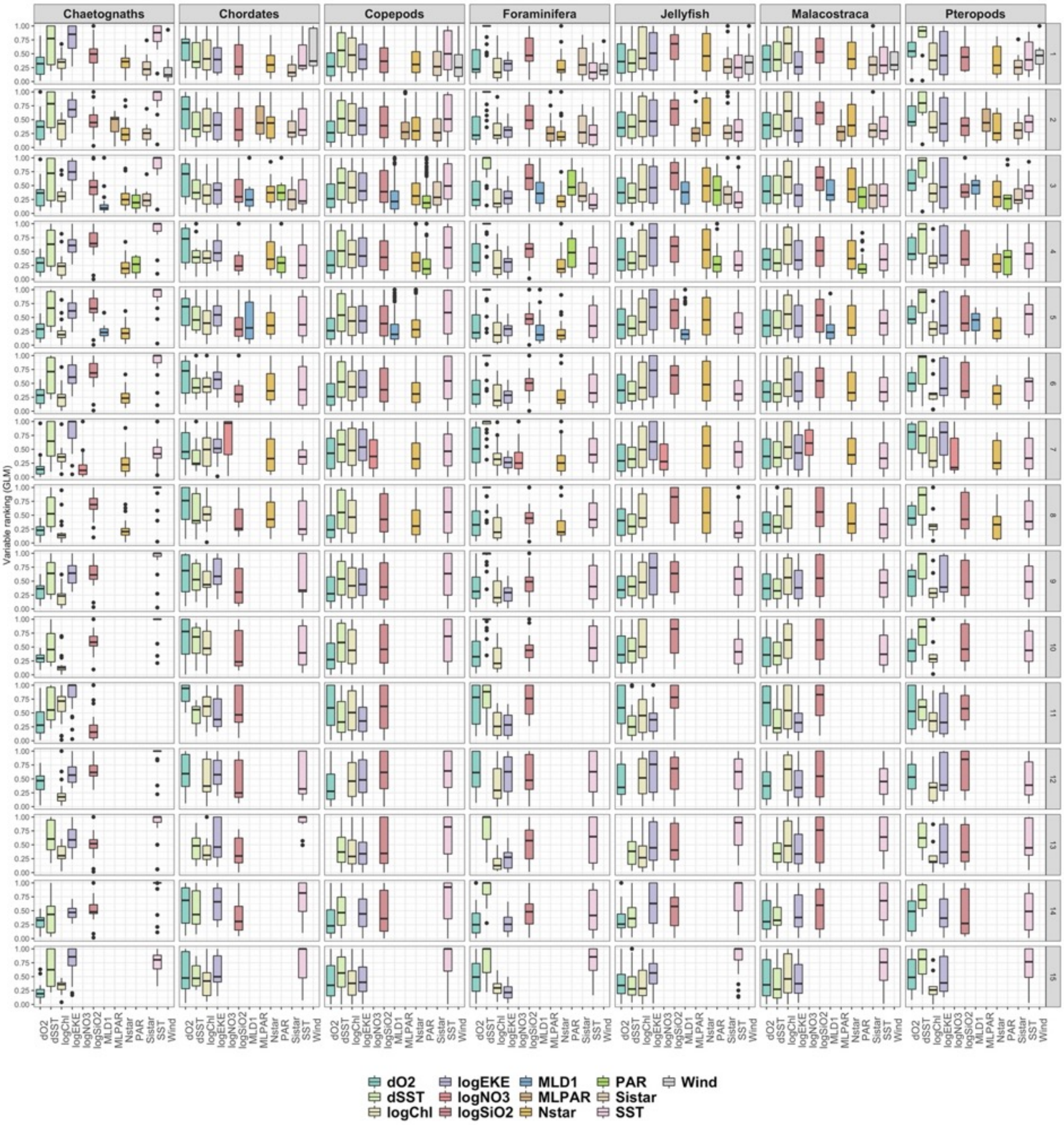

Supplementary Note 1-7: Distribution of the normalized ranks of relative importance of each environmental predictor across all of the 15 sets described above, for the seven main zooplankton groups based on Generalized Linear Models (GLM). The lower, middle and upper boundaries of the boxplots correspond to the 25th, 50th and 75th percentiles. The lower and upper whiskers extend from the hinges to the lowest or largest value no further than 1.5*IQR (inter-quartile range) from the lower and upper hinges. The total sample size is N = 5565 models (371 zooplankton species x 15 predictors sets) and per group is: N = 375 for Chaetognaths, N = 165 for Chordates, N = 3135 for Copepods, N = 345 for Foraminifera, N = 585 for Jellyfish, N = 690 for Malacostraca, and N = 150 for Pteropods.

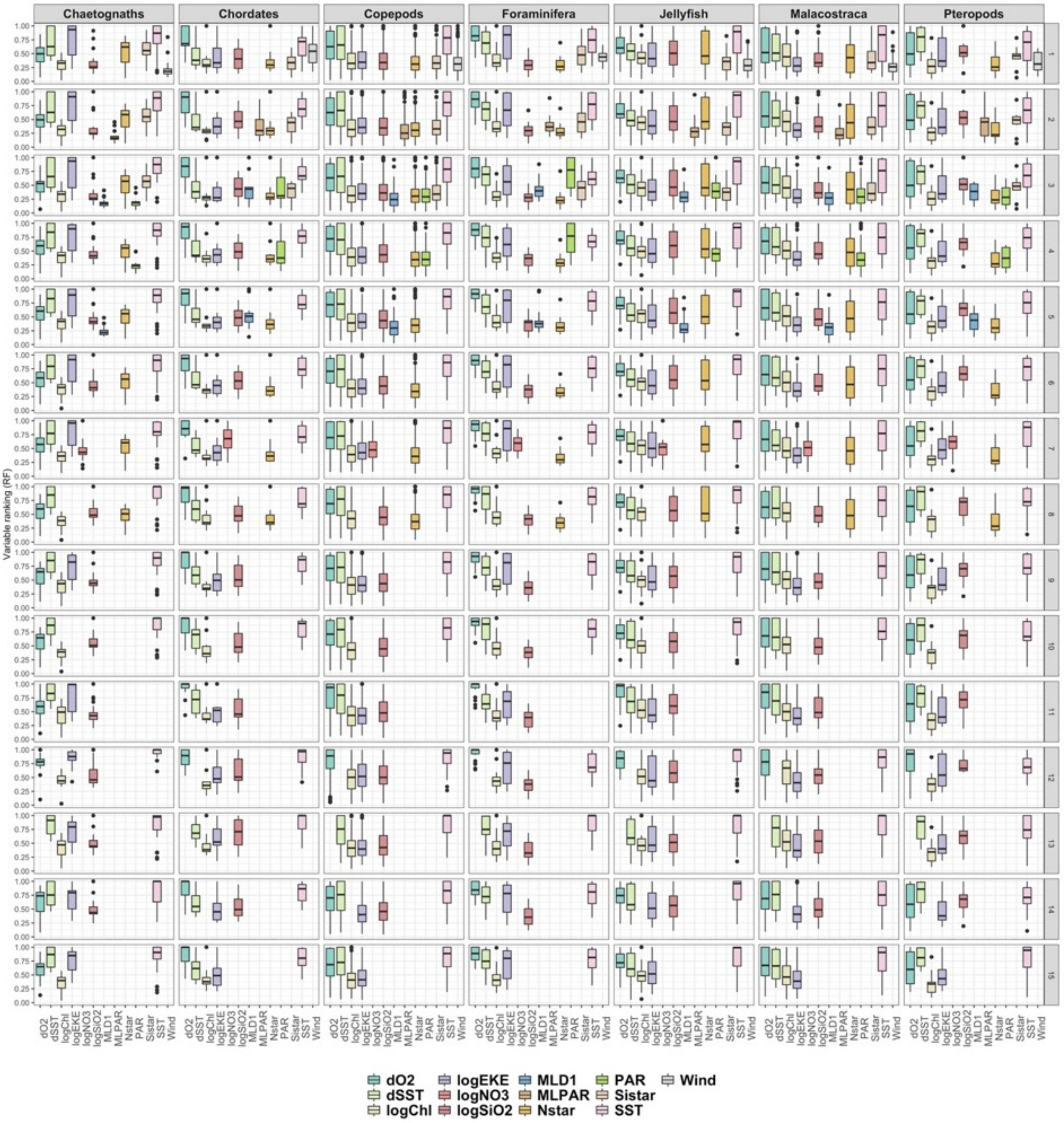

Supplementary Note 1-8: Distribution of the normalized ranks of relative importance of each environmental predictor across all of the 15 sets described above, for the seven main zooplankton groups based on Random Forest (RF) models. The lower, middle and upper boundaries of the boxplots correspond to the 25th, 50th and 75th percentiles. The lower and upper whiskers extend from the hinges to the lowest or largest value no further than 1.5*IQR (inter-quartile range) from the lower and upper hinges. The total sample size is N = 5565 models (371 zooplankton species x 15 predictors sets) and per group is: N = 375 for Chaetognaths, N = 165 for Chordates, N = 3135 for Copepods, N = 345 for Foraminifera, N = 585 for Jellyfish, N = 690 for Malacostraca, and N = 150 for Pteropods.

To select the sets of predictors to be used in the SDMs, preliminary Generalized Linear Models (GLM) and Random Forest (RF) models were performed for each species with several predictors sets of decreasing complexity (from 10 to 5). The explanatory power (adjusted R^2) of the models and the ranking of predictors within each set were extracted and their changes in distribution between the various sets were examined. This was done for total phytoplankton and total zooplankton separately, and then between their respective constituting groups: Bacillariophyta (Diatoms), Dinoflagellata (Dinoflagellates) and Haptophyta (Coccolithophores) for phytoplankton, and Copepoda, Chaetognatha, Pteropoda, Malacostraca, Jellyfish, Chordata and Foraminifera for zooplankton. This way, we identified the most important predictors for modelling the species distributions and to evaluate if a decrease in the models' skill was linked to the removal of certain variables. Group patterns allowed to test whether different groups differed in their main environmental drivers.

For phytoplankton species, 14 sets of variables were examined. The first nine aimed to test the impact of: (i) alternative choices between variables that were identified as collinear (Wind *vs*. MLPAR *vs*. MLD+PAR); (ii) progressively discarding variables that presented lower ranks (logEKE, Si^*); and (iii) choosing logNO3 over logSiOH4, two variables embodying global macronutrients availability and that presented relatively high correlation coefficient ($\rho = 0.59$). The last five sets of predictors (10-14) allowed to test the impact of alternatively removing those variables that presented relatively high ranks in the previous sets: SST, dSST, N^*, logSiOH4, logChl, PAR.

Similarly, 15 sets of variables were tested for zooplankton. The first ten aimed to test the impact of: (i) choosing Wind over MLPAR or over MLD+PAR; (ii) selecting PAR over MLD; (iii) discarding Si^*, N^*, logEKE; and (iv) choosing logNO3 over logSiOH4 (ρ=0.64). The last five sets of predictors (11-15) aimed to test the impact of alternatively discarding the top 5 predictors: SST, dSST, dO2, logSiOH4, and logChl.

All the resulting R^2 and predictors ranks are shown in the Supplementary Note 1-1 above. Pairwise non parametric variance analyses (Kruskal-Wallis tests) were carried out to test for significant increase/decrease in the models' R^2 between pairs of predictors set. We now describe those results to clarify our final choices of predictors sets mentioned in the main Methods section. First, we describe the variations in the models' R^2 for the phytoplankton species (and their three main groups) across the predictors sets. Second, we summarize the overall predictors ranking for all phytoplankton and their three groups. The results relative to the zooplankton species will be described right after.

Phytoplankton

The first three sets allowed to test between sets including Wind (Set 1), or MLPAR (Set 2) or MLD+PAR (Set 3). No differences in R^2 were detected for Diatoms and Dinoflagellates, but R^2 were higher for Haptophyta when using Set 3 so the latter was kept.

Sets 4 and 5 tested the differences between PAR and MLD. Set 4 exhibited significantly higher R^2 than Set 5 for Diatoms only. So PAR was preferred over MLD.

Sets 3 and 4 tested the effect of removing Si*. No changes in R^2 were observed when removing Si* so it was discarded for the next tests because we favoured more parsimonious sets.
Sets 5 and 6 tested whether using logNO3 (Set 5) over logSiOH4 (Set 6) lead to higher models of explanatory power. We found no significant changes in R^2 between the two sets, so either one of the two predictors will be used in the alternative final predictors sets. Comparing Sets 4, 5 and 6 together also showed that using one of the two or both at the same time does not change R^2 distributions.
Sets 6 and 7 tested the effect of including logEKE. No significant differences in R^2 were found so logEKE was discarded for phytoplankton. This was also supported by testing changes in R^2 between Sets 8 (with logEKE) and 9 (without logEKE).
Comparing Set 7 to Set 9, and Set 6 to Set 8 allowed to test the effect of removing N*. A significant drop in R^2 was found for Dinoflagellates (R^2 did not vary for the two other groups) so N* was kept.
Comparing Set 7 to the last five sets (10-14) allowed to test the effect of removing those five variables that seemed essential from the previous tests (and the variable importance rankings): SST (Set 10), dSST (Set 11), logNO3/logSiOH4 (Set 12), logChl (Set 13) and PAR (Set 14). According to GLM, removing either SST, logChl and logNO3/logSiOH4 decreased model skill significantly for Diatoms. Similarly, the models built for Dinoflagellates exhibited significant drops in R^2 when discarding SST and logChl. For Haptophyta, removing PAR and SST lead to models of lower explanatory power. According to RF models, removing dSST decreased model skill for all groups.

Examining the distribution of the predictors' relative importance in the GLM and RF allowed us to establish the following ranking of variables for phytoplankton (rankings within phytoplankton groups are not explicitly detailed here but are shown in Supplementary Note 1-3,4):

- **GLM**: SST >> N* > logChl > logSiOH4 > Wind > dSST/logNO3 > logEKE/MLPAR/Si* > MLD > PAR
- **RF**: SST > N* >> logChl > dSST > logSiOH4/Wind > PAR > logEKE > logNO3 > Si* > MLPAR/MLD

Consequently, in combination with the results described above, four final sets of predictors chosen for the SDMs were:

1. **SST, dSST, logChl, N*, PAR, and logNO3**

2. **SST, dSST, logChl, N*, PAR, and logSiOH4**

3. **SST, dSST, logChl, N*, PAR, logNO3 and Si***

4. **SST, dSST, logChl, PAR, and logNO3**

Zooplankton

Like for phytoplankton, the first three sets allowed to test between sets including Wind (Set 1), MLPAR (Set 2) and MLD+PAR (Set 3). R^2 slightly increased from Set 1 to Set 3 for all groups, but the sharpest increase was observed for Foraminifera according to GLM and RF. Comparing Sets 4 (with PAR instead of MLD) and 5 (MLD instead of PAR) enabled us to identify PAR as the main driver of these variations in model skill, as removing PAR decreased R^2. Therefore, PAR was kept as a potential predictor as it seemed key to model Foraminifera species.

Comparing Set 3 to Sets 4 and 5 allowed to test the effect of removing Si* as the latter is absent in Sets 4 and 5 but not 3. A slight but significant decrease in the R^2 of GLM was observed for Copepoda, Jellyfish and Foraminifera. Therefore we kept it in the list of potential predictors and waited to further examine its relative importance in the models.

Comparing Set 4 to Set 6 allowed to test the effect of removing PAR. No significant changes in R^2 were found so PAR was discarded to favour predictor parsimony.

Similarly, MLD was also removed since comparing Set 5 to Set 6 (no MLD) showed that it did not improve the models R^2.

Comparing Set 6 to Set 7 allowed to test the effect of choosing logNO3 (Set 7) over logSiOH4 (Set 6). The GLM built for the Chaetognatha showed a slight decrease in R^2 when choosing logNO3. But this result was not confirmed by the RF that showed a slight increase in R^2 for Chordata when picking nitrates over silicates. Like for phytoplankton, both macronutrients variables were kept and could be alternatively chosen in the final predictors sets.

Comparing Set 6 to Set 8, and Set 9 to Set 10, allowed to test the impact of removing logEKE. GLMs and RF showed a decrease in R^2 for Copepoda and Chaetognatha, so logEKE was kept.

Comparing Set 6 to Set 9, and Set 8 to Set 10, allowed to test the impact of removing N*. Neither GLM nor RF showed a change in R^2 so N* did not appear as a key predictor for most of the species. However, we considered keeping it because it could be used as a predictor to embody gradients in nitrates availability when logSiOH4 is used instead of logNO3, without decreasing model skill.

Comparing Set 9 to the last five sets (11-15) allowed to test the effect of removing those five variables that seemed essential from the previous tests (and the variable importance rankings): SST (Set 11), dSST (Set 12), dO2 (Set 13), logChl (Set 14) and logSiOH4/logNO3 (Set 15). Removing SST significantly decreased the R^2 of GLM for Copepoda, Chaetognatha and Malacostraca. Surprisingly, no changes in the R^2 of the RF models were found. On the contrary, both RF and GLM found significant decreases in models R^2 for Copepoda, Jellyfish, Chaetognatha, Pteropoda, Malacostraca and Foraminifera when removing dSST, thus suggesting this variable is quite relevant for modelling the species' distribution. Removing dO2 significantly decreased the models' R^2 for some groups, but those differed according to the type of model: Malacostraca and Chordata with GLM, Jellyfish, Malacostraca and Foraminifera with RF. Omitting logChl from the predictors weakened the GLM explanatory power for Copepoda and Malacostraca (none with the RF). And finally, removing either logSiOH4 or logNO3 significantly decreased the R^2 of GLM for:

Copepoda, Chaetognatha, Jellyfish, Malacostraca and Foraminifera. Again, RF were better able to accommodate this loss than GLMs as no significant decrease in their R^2 could be observed.

Examining the distribution of the predictors' relative importance in the GLM and RF allowed us to rank the variables for total zooplankton (rankings within zooplankton groups are not explicitly detailed here but they are shown in Supplementary Note 1-7,8). GLM and RF provided slightly different rankings, which help explain why the models' R^2 sometimes decreased according to one type of model but not the other. Therefore, we chose to clarify the rankings for both types of algorithms:

- **GLM**: SST > dSST/logSiOH4 > logEKE/logChl > logNO3/dO2 > N* > Si*/MLPAR/Wind > MLD/PAR
- **RF**: SST > dO2/dSST >> logNO3 > logSiOH4 > logEKE > logChl > N*/Si* > PAR > Wind > MLD > MLPAR

Please note how the distributions of ranks were much less even between the predictors when using RF instead of GLM, especially for the variables that usually ranked lower than SST. This showed why the R^2 of the RF models were much less affected when removing a predictor other than SST, dSST and dO2.

Consequently, in combination with the results described above, four final sets of predictors chosen for the SDMs were:

1. SST, dSST, dO2, logChl, and logNO3

2. SST, dSST, dO2, logChl, and logSiOH4

3. SST, dSST, dO2, logChl, logSiOH4 and N*

4. SST, dSST, dO2, logChl, logNO3 and Si*

Supplementary Note 2 - Spatial overlap analysis between climate change impacts on global surface plankton diversity and the current provision of marine ecosystem services

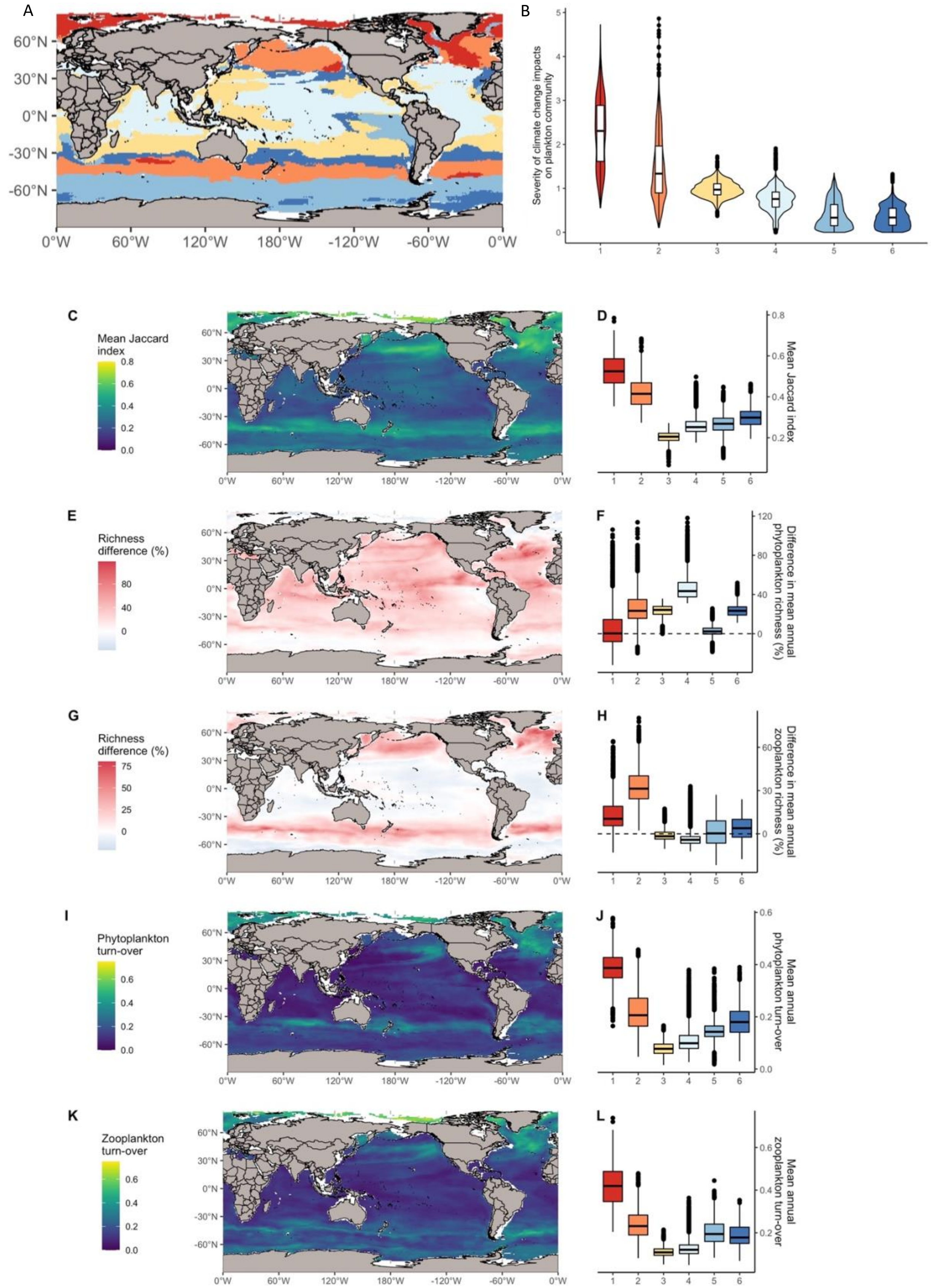

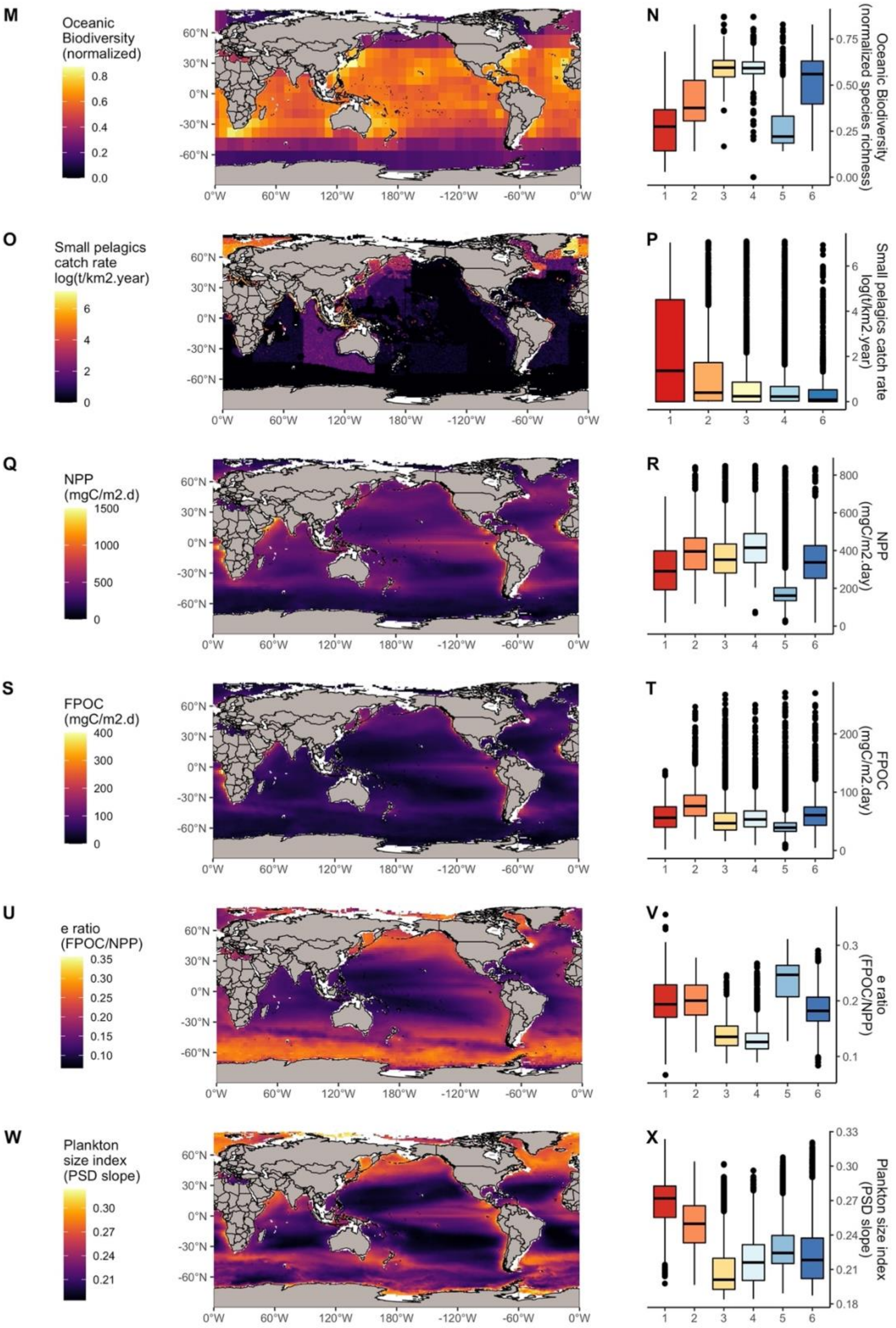

Supplementary Note 2: Spatial overlap analysis between climate change impacts on global surface plankton diversity and the current provision of marine ecosystem services. A clustering approach defined and ranked (A) and (B) six regions based on

(C) to (L) five variables summarizing the ensemble projections of phyto- and zooplankton diversity: (C) and (D) mean dissimilarity in annual community composition, (E) and (F) mean percentage difference in annual phytoplankton species richness, (G) and (H) mean percentage difference in annual zooplankton species richness, (I) and (J) mean turn-over in phytoplankton annual species composition and (K) and (L) mean turn-over in zooplankton annual species composition. Six variables were used as proxies for marine ecosystem services and their distribution across the six regions: (M)-(N) oceanic biodiversity (normalized species richness of the marine megafauna that mainly inhabit the open ocean), (O)-(P) logged mean annual reported and unreported catch rates of small (< 30cm) pelagic fishes, (Q)-(R) mean annual net primary production (NPP; mg C m-2 d-1), (S)-(T) mean annual flux of particulate organic carbon (FPOC; mg C m-2 d-1) below the euphotic zone, (U)-(V) mean annual FPOC/NPP ratio (e ratio), and (W)-(X) mean annual plankton size index (i.e. inverse of the slope of the particles size distribution). The lower, middle and upper boundaries of all the boxplots correspond to the 25th, 50th and 75th percentiles. The lower and upper whiskers extend from the hinges to the lowest or largest value no further than 1.5*IQR (inter-quartile range) from the lower and upper hinges. N = 35023 grid cells for the total sample size ($N_{region1} = 2344$; $N_{region2} = 5954$; $N_{region3} = 6748$; $N_{region4} = 8290$; $N_{region5} = 7637$; $N_{region6} = 4050$).

The ensemble projections of climate change impacts on plankton species richness and composition were summarized using a Principal Component Analysis (PCA; see Methods section) whose principal components were used to compute a (B) quantitative index of the relative severity of climate change impacts on plankton diversity whose distribution across the newly-defined regions was used to rank them in decreasing order. The spatial distribution and the regional variations of (M)-(X) six proxy variables of marine ecosystem services were examined per region: (M)-(N) Oceanic Biodiversity (normalized species richness of the marine megafauna that mainly inhabit the open ocean [1]), (O)-(P) logged mean annual reported and unreported catch rates of small (< 30cm) pelagics fisheries [2], (Q)-(R) mean annual Net Primary Production (NPP; mg C m^{-2} d^{-1}) [3], (S)-(T) mean annual flux of particulate organic carbon (FPOC; mg C m^{-2} d^{-1}) below the euphotic zone [3], (U)-(V) mean annual FPOC/NPP ratio (e ratio) [34], and (W)-(X) mean annual plankton size index (i.e. inverse of the slope of the particles size distribution) [4]. Nonparametric variance analyses (Kruskal-Wallis tests) were performed to test the significance level of the regional variations in plankton diversity changes and proxy variables of marine ecosystem services (0.01 significance threshold). Normality and homoscedasticity tests were performed prior to the Kruskal-Wallis tests. Pairwise post hoc tests of multiple comparisons of mean rank sums were performed to identify which pairs of regions displayed those significant variations. Bonferroni corrections were applied for p-values adjustment.

For the ensemble projections of plankton diversity changes, all five variables displayed significant variations across the six regions (all $p < 0.001$) and post hoc tests found all pairwise comparisons to be significant except for (F) the mean percentage difference in annual phytoplankton species richness between regions 3 and 6. For the proxy

variables of marine ecosystem services, all six variables displayed significant variations across the six regions (all $p < 0.001$) and post hoc tests found all pairwise comparisons to be significant except for the variations in: (N) Oceanic Biodiversity between regions 3 and 4 and between regions 1 and 5; (P) annual small pelagics catch rates between regions 3 and 4; (T) mean annual FPOC between regions 1 and 4; and (V) e ratio between regions 1 and 2. Because the majority of low catch rates for the Southern Ocean are due to a lack of reported data, region 5 was discarded when comparing mean annual fisheries catch rates.

Supplementary Note 3: List of the characters used to identify the datasets in GBIF and OBIS issued from sediment cores that provide sediment or fossil species records.

Zooplankton groups that are characterized by a calcareous or a siliceous shell (e.g. Foraminifera, Thecosomata, and Radiolaria) are often found as fossils in sediment cores where they are used as paleo-indicators of past environmental conditions. As a result, many of these groups' biological observations are archived in online biodiversity repositories come from deep sediment cores. This is particularly relevant for Foraminifera with the advent of global sediment core databases such as CLIMAP, MARGO or ForCenS [5]. In the context of our study, that aims to model extant species diversity from observations collected in the surface ocean, it is critical to remove such observations from the OBIS and GBIF datasets we used.

To do so, we first examined the names of the datasets we downloaded from OBIS ('bibliographicCitation' column) and GBIF (using the '*datasets*' function of the *rgbif* R package). Then, we excluded those that contained at least of the following keywords: *sediment, paleo, Hole, core, CLIMAP, ODP, temperature reconstruction, DSDP, Oligocene, Neogene, Miocene, Pliocene, Holocene, Paleocene, Pleistocene, abundance of Hole, SST, sedimentological, GeoB, sediments, stratigraphically, time slice, Sediment, reconstruction, last glacial maximum, Site GIK, fossils, Shipboard Scientific Party, DSDP Site, lithic, meiofauna, Sedimentological, sediment corev, Stable oxygen isotope, Paleocene-Eocene, Cretaceous, Maastrichtian, K/T, Benth, benthic, AMK21, Discovery Reports, NU2_trap, trap L2.*

Finally, the names of the remaining datasets were inspected again, and those that were not discarded but still presented equivocal names (e.g. '*Earland, A. 1934. Foraminifera. Part III.*', '*Electron Micrograph Database - Marine Specimens*', or '*Arctic Ocean Diversity*') were examined through an online literature research to verify if they contained fossil records.

Supplementary Note 4: Analyzing the potential biases induced by the inclusion of sea surface salinity (SSS) and surface CO_2 concentration (pCO_2) in the species distribution models.

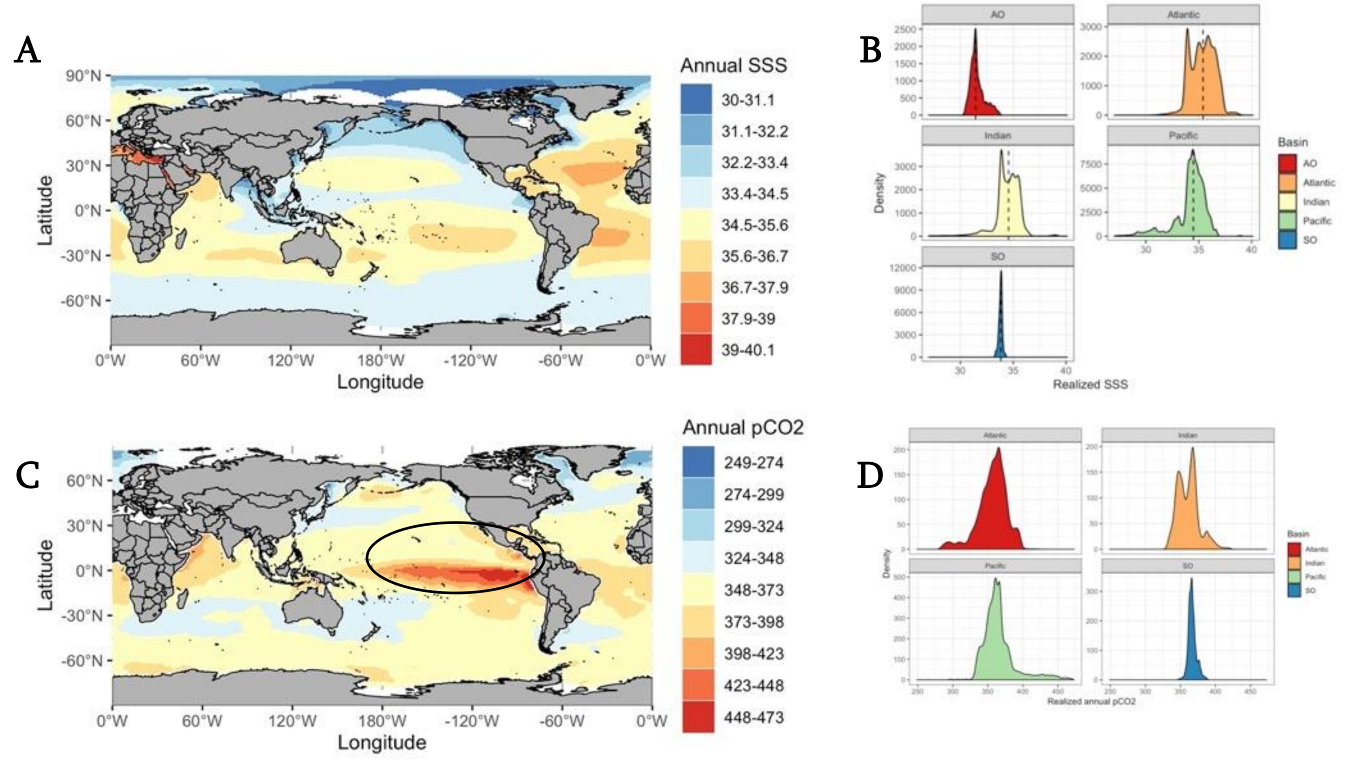

Supplementary Note 4-1: Spatial distribution of (A)-(C) the global annual average and (B)-(D) the corresponding density distribution across the five main ocean basins (AO = Arctic Ocean, SO = Southern Ocean) of (A)-(B) sea surface salinity (SSS) and (C)-(D) partial pressure of CO_2 (pCO_2). Data are lacking for the Mediterranean Sea, the Red Sea and the AO for pCO_2. The SSS values below 30 were not shown to highlight the inter-basins differences in SSS. The dotted lines in (B) illustrate the average SSS value of the basins. Sample size is N = 41088 grid cells for (A)-(B) and N = 33790 grid cells [illegible]

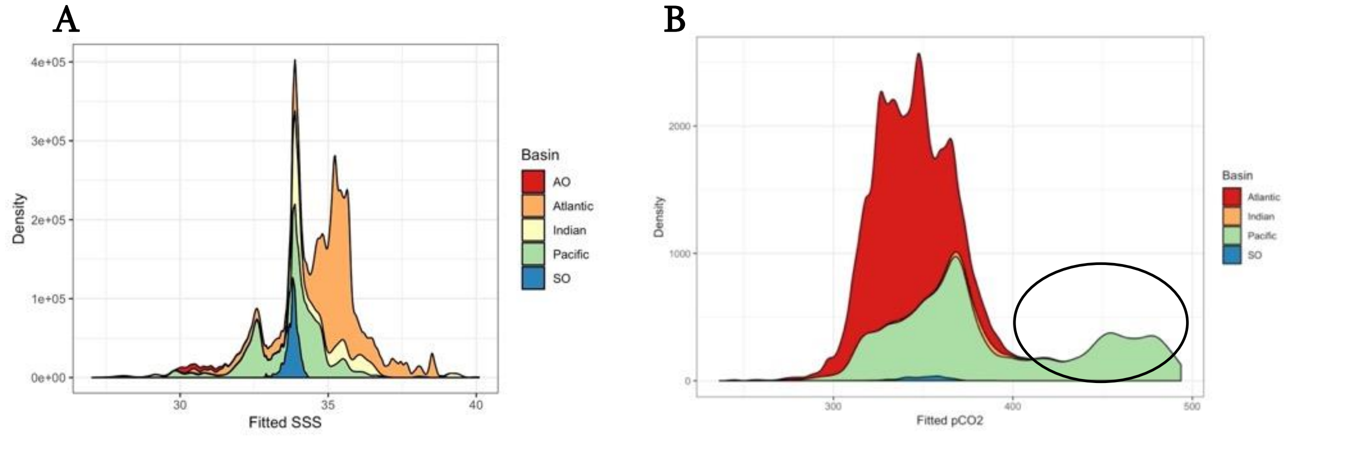

Supplementary Note 4-2: Inter-basin differences in the distribution of (A) SSS and (B) pCO2 values fitted to (A) the monthly zooplankton occurrences and (B) the monthly phytoplankton occurrences. (A) highlights how the zooplankton occurrences are biased towards the higher SSS values found in the Atlantic basin, whereas (B) highlights how the phytoplankton occurrences are biased towards the highest values of pCO_2 because of the sampling bias towards the Peruvian upwelling as the pCO_2 values fitted to the phytoplankton occurrences clearly inflate the higher range of pCO_2 gradient compared to the realized global gradient.

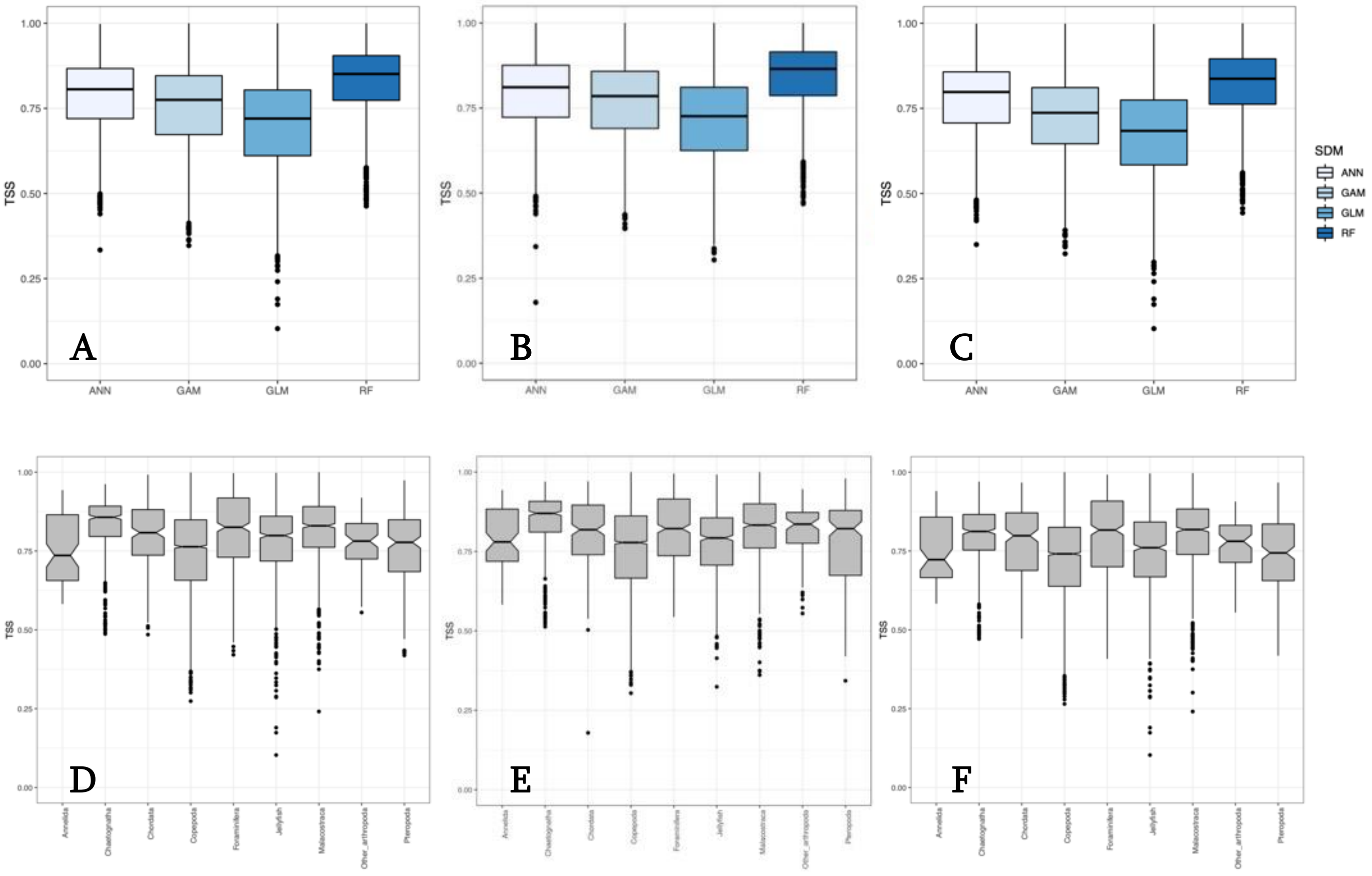

Supplementary Note 4-3: Distribution of SDMs True Skill Statistic (TSS) values for (A)-(B)-(C) each algorithm type (Artificial Neural Networks, Generalized Additive Models, Generalized Linear Models and Random Forest respectively) and (D)-(E)-(F) every zooplankton taxonomic group. SDMs were run across three sets of environmental predictors to test the potential impact of removing sea surface salinity (SSS) from the predictors, and to assess its role as a basin indicator in the SDMs. Set 1 (A)-(D) included sea surface temperature (SST), SSS, annual range of SST (dSST), dissolved oxygen concentration at 175m depth (dO2), logged surface Chlorophyll concentration (logChl) and logged Nitrates concentration (logNO3). Set 2 (B)-(E) included SST, dSST, dO2, logChl, logNO3 and Longitude (0°-360°) instead of SSS. Set 3 (C)-(F) included SST, dSST, dO2, logChl and logNO3. The lower, middle and upper boundaries of all the boxplots correspond to the 25th, 50th and 75th percentiles. The lower and upper whiskers extend from the hinges to the lowest or largest value no further than 1.5*IQR (inter-quartile range) from the lower and upper hinges. For each boxplot shown in (A)-(B)-(C), sample size is N = 3710 (371 zooplankton species x 10 cross evaluation runs). For the boxplot shown in (D)-(E)-(F), the sample size is (N species modelled x 10 cross evaluation runs): N = 40 for Annelida, N = 250 for Chaetognaths, N = 110 for Chordates, N = 2090 for Copepods, N = 230 for Foraminifera, N = 390 for Jellyfish, N = 460 for Malacostraca, N 40 for Other Arthropoda, and N = 100 for Pteropods.

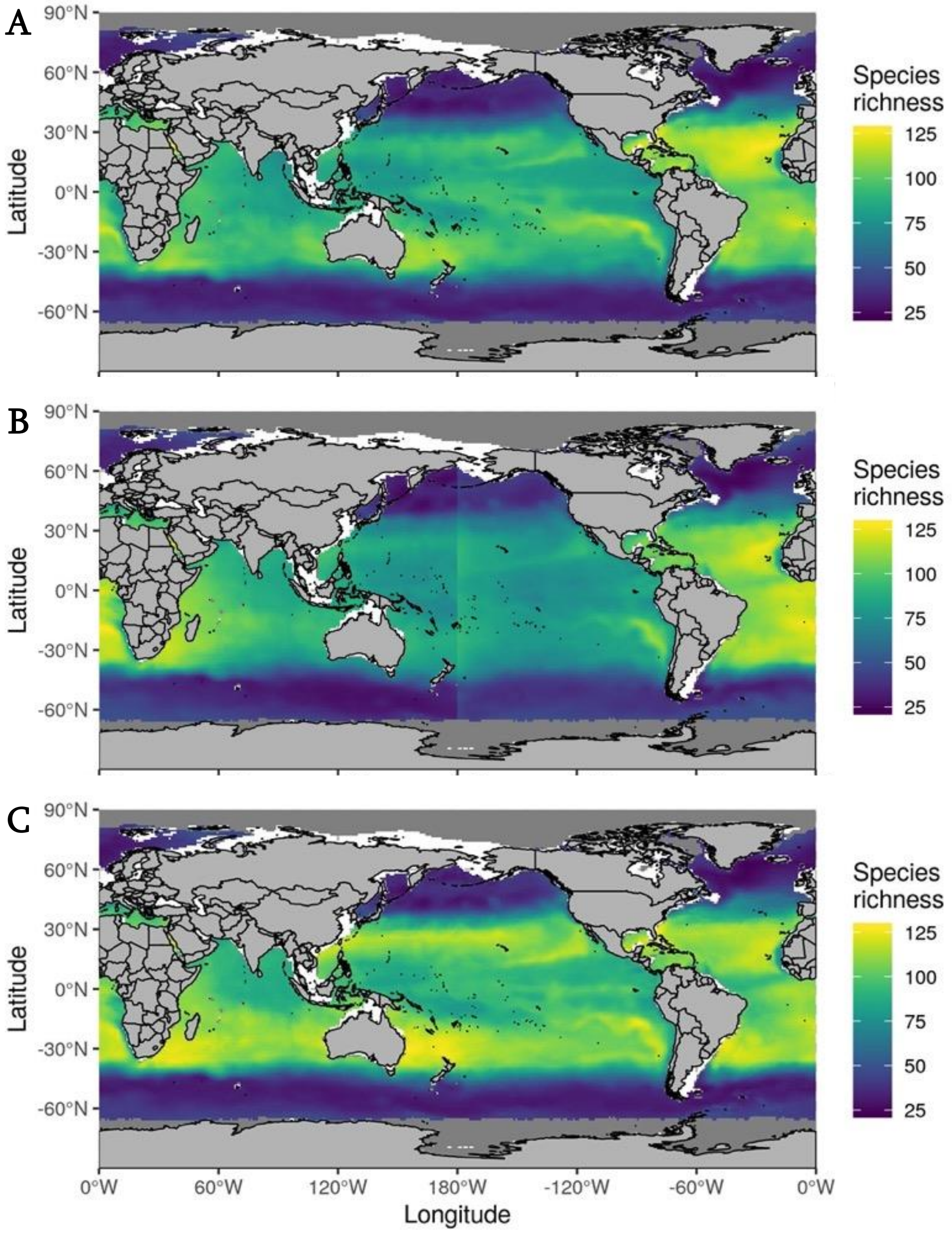

Supplementary Note 4-4: Spatial distribution of zooplankton species richness in April (estimated through the sum of species average habitat suitability indices across all SDMs presenting an average TSS > 0.3) for the three sets of environmental predictors aiming to test the role of SSS as a basin indicator in the SDMs and how removing it might affect the global zooplankton diversity pattern. Set 1 (A) included sea surface temperature (SST), SSS, annual range of SST (dSST), dissolved oxygen concentration at 175m depth (dO2), logged surface Chlorophyll concentration (logChl) and logged Nitrates concentration (logNO3). Set 2 (B) included SST, dSST, dO2, logChl, logNO3 and Longitude (0°-360°) instead of SSS. Set 3 (C) included SST, dSST, dO2, logChl and logNO3.

As shown by the differences between maps (A), (B) and (C), including SSS or Longitude as a predictor leads to peaks in richness being concentrated in the Atlantic Ocean, whereas removing it leads to more even richness values across the basins without significantly decreasing SDMs skill. We interpret these differences in diversity patterns as the result artifactual effect caused by the concentration of zooplankton occurrences in the Atlantic Ocean and an ensuing bias towards higher SSS values in the SDMs. We do not neglect here the potential role of SSS in structuring plankton communities in the surface ocean. But, in light of the biases in environmental space evidenced here that are leading to obvious differences in the resulting species richness patterns, we chose to discard SSS and pCO_2 from the list of potential environmental predictors.

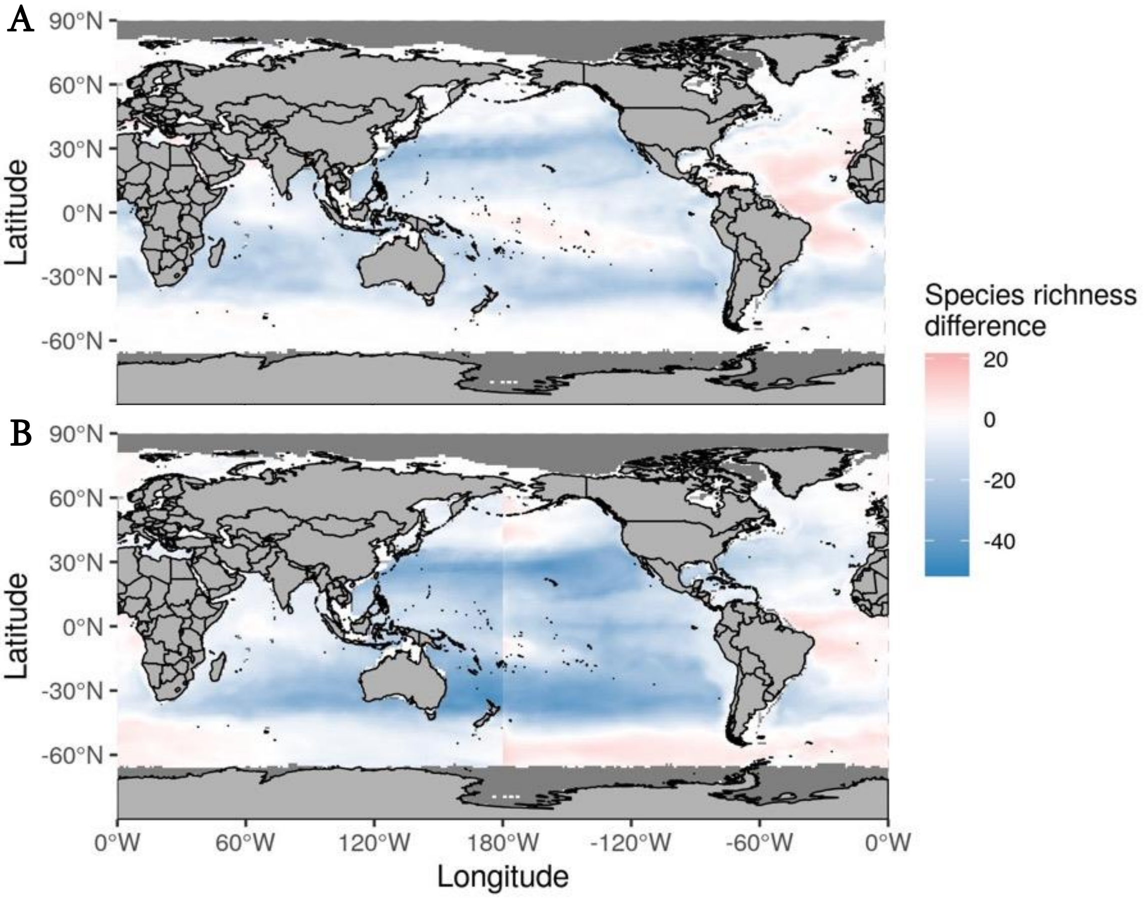

Supplementary Note 4-5: Difference in zooplankton species richness in April between (A) Set 1 and Set 3, and (B) Set 2 and Set 3. Set 1 included sea surface temperature (SST), SSS, annual range of SST (dSST), dissolved oxygen concentration at 175m depth (dO2), logged surface Chlorophyll concentration (logChl) and logged Nitrates concentration (logNO3). Set 2 included SST, dSST, dO2, logChl, logNO3 and Longitude (0°-360°) instead of SSS. Set 3 included SST, dSST, dO2, logChl and logNO3. The similarity between map (A) and map (B) shows how including SSS in the predictors may act as an indicator of ocean basins (like Longitude) and underestimate species richness in the Pacific Ocean and the Indian Ocean and overestimate it in the Atlantic Ocean.

Supplementary Note 5: ODMAP protocol

Major restructuring of marine plankton assemblages under global warming.

– ODMAP Protocol –

Fabio Benedetti, Meike Vogt, Urs Hofmann Elizondo, Damiano Righetti, Niklaus E. Zimmermann, Nicolas Gruber

2021-05-18

Overview

Authorship

Contact : fabio.benedetti@usys.ethz.ch

Study link: Manuscript submitted to Nature Communications #NCOMMS-20-37764C

Model objective

Model objective: Forecast and transfer

Target output: Continuous habitat suitability indices to estimate assemblages richness and composition.

Focal Taxon

Focal Taxon: Marine plankton (main phytoplankton and zooplankton groups).

Location

Location: Global surface ocean

Scale of Analysis

Spatial extent: -180, 180, -90, 90 (xmin, xmax, ymin, ymax)

Spatial resolution: 1°x1°

Temporal extent: 1800 to 2018

Temporal resolution: Monthly climatologies

Boundary: natural

Biodiversity data

Observation type: field survey, standardised monitoring data

Response data type: presence-only

Predictors

Predictor types: climatic, habitat

Hypotheses

Hypotheses: See Assumptions section below.

Assumptions

Model assumptions: our SDMs rely on the following main assumptions: (i) niche conservatism through time; (ii) species distributions are not strongly limited by dispersal at a macroecological scale, an assumption valid for plankton considering the very strong connectivity of ocean basins through surface current on decadal scales, which enables plankton species to display very large spatial ranges; (ii) at the scale of the study, species spatial distributions are primarily shaped by the combinations of environmental factors (not biotic interactions) that define the conditions allowing a species to develop.

Algorithms

Modelling techniques: glm, gam, randomForest, ann

Model complexity: To cover range of models types (regression, classification trees, neural networks) and model complexity.

Model averaging: Models projections (habitat suitability indices) were averaged without weights.

Workflow

Model workflow: See online Methods section upon publication of the study.

Software

Software: biomod2

Code availability: All codes available on the GitHib page of the first author: https://github.com/benfabio

Data availability: All occurrence data obtained from publicly available datasets.

Data

Biodiversity data

Taxon names: 860 Plankton species (336 phytoplankton species and 524 zooplankton species) - see Supplementary Data 2 for the list of species names and their taxonomic classification.

Taxonomic reference system: WoRMS (https://www.marinespecies.org/) for zoopankton species; Algaebase (http://www.algaebase.org/) for phytoplankton species.

Ecological level: species, communities

Data sources: OBIS (https://obis.org/) + GBIF (https://www.gbif.org/) (both accessed between the 12 and the 13 of April 2018) + Cornils et al. (2018) + PHYTObase (Righetti et al., 2020)

Sampling design: Random

Sample size: 934,606 occurrences across all species modelled (226,835 for phytoplankton, 707,861 for zooplankton).

Clipping: Not applicable.

Cleaning: See online extended Methods upon publication of the study (+Supplementary Table S7).

Absence data: No absence data available.

Background data: Species-level background data drawn based on the target-group approach of Philipps et al. (2009).

Errors and biases: See online extended Methods upon publication of the study.

Data partitioning

Training data: Randomly selected 80%.

Validation data: Randomly selected 20% withheld from model fitting.

Test data: Not available.

Predictor variables

Predictor variables: Sea surface temperature (SST), annual range of SST (dSST), dissolved oxygen concentration at 175 depth (dO2), logged surface Chlorophylla concentration (logChla), logged surface nitrates concentration (logNO3), logged surface silicates concentration (logSiOH4), excess of nitrates to phosphates (N*), excess of silicates to nitrates (Si*), and surface photosynthetically available radiation (PAR).

Data sources: World Ocean Atlas 2013v2 (https://www.nodc.noaa.gov/OC5/woa13/), GlobColour for Chlorophylla (https://hermes.acri.fr/), OceanColour for PAR (https://oceancolor.gsfc.nasa.gov/data/seawifs/).

Spatial extent: -180, 180, -90, 90 (xmin, xmax, ymin, ymax)

Spatial resolution: 1°x1°

Coordinate reference system: CRS WGS84

Temporal extent: Monthly climatologies based on in situ data covering the past 20 years. Except dSST which was defined on an annual scale.

Temporal resolution: In situ measurements cover the 1955-2012 time period (WOA13v2 climatologies).

Data processing: See Methods description available on the WOA13 data portal (https://www.nodc.noaa.gov/OC5/woa13/).

Errors and biases: See Methods description available on the WOA13 data portal (https://www.nodc.noaa.gov/OC5/woa13/).

Dimension reduction: Not applicable.

Transfer data

Data sources: 5 Earth System Models from the Coupled Model Intercomparison Project - Phase 5 (CMIP5)

Spatial extent: -180, 180, -90, 90 (xmin, xmax, ymin, ymax)

Spatial resolution: 1°x1°

Temporal extent: Monthly climatologies computed from the monthly outputs of the each CMIP5 Earth System Model for the 2081-2100 time period.

Temporal resolution: Monthly Earth System Model outputs.

Models and scenarios: 5 Earth System Models forced by RCP8.5 greenhouse gas concentration scenario of the IPCC.

Data processing: See online Methods upon publication of the study.

Quantification of Novelty: Multivariate environmental similarity surface (MESS) technique (Elith et al., 2010).

Model

Variable pre-selection

Variable pre-selection: See online extended Methods upon publication of the study.

Multicollinearity

Multicollinearity: Exclusion of one of two environmental predictors when pairwise Spearman rank correlation coefficient > |0.70| (Dormman et al., 2013).

Model settings

- glm: type (quadratic), interaction.level (0), family (binomial logit link), test (AIC), control (glm.control(epsilon = 1e-08, maxit = 50, trace = FALSE))
- gam: Original algorithm ('mgcv' described in Wood, 2017 - doi:10.1201/9781315370279), family (binomial logit link), smoothTerms (k) (5), interaction.level (0), method (GCV.Cp), select (TRUE), knots (NULL), paramPen (NULL), optimizer ('outer' and 'newton')
- randomForest: ntree (750), mtry (number of predictors (p) / 3), nodesize (10), maxnodes (NULL, limited by nodesize)

- ann: maxit (max nb of iterations) (200), size (nb of hidden layers) (Optimised by cross validation based on model AUC (for n = NbCV)), decay (Optimised by cross validation based on model AUC (for n = NbCV)), Nb of cross validation (NbCV) (5)

Model settings (extrapolation): Non applicable.

Model estimates

Coefficients: See Model settings above.

Variable importance: See online extended Methods upon publication of the study.

Model selection - model averaging - ensembles

Model averaging: Arithmetic mean of the models projections of species habitat suitability indices (no weighting by SDM evaluation metric).

Analysis and Correction of non-independence

Spatial autocorrelation: Thinning of species occurrences over 100km.

Nested data: Not applicable.

Threshold selection

Threshold selection: Non applicable. Diversity estimates were based on the species' continuous average habitat suitability indices.

Assessment

Performance statistics

Performance on training data: TSS, AUC, Kappa

Performance on validation data: TSS, AUC, Kappa

Performance on test data: Not available.

Plausibility check

Response shapes: Examination of SDM-specific mean univariate response curve for every species modelled.

Expert judgement: Examination of SDM-specific mean annual habitat suitability maps for every species modelled.

Prediction

Prediction output

Prediction unit: Monthly and mean annual species richness estimated through sum of species habitat suitability indices.

Post-processing: Not applicable.

Uncertainty quantification

Parameter uncertainty: Not applicable.

Scenario uncertainty: Uncertainties in ensemble projections were examined through the standard deviation computed from the ensemble model members projections (n = 16 for contemporary conditions; n = 80 for end of century conditions since 5 Earth System Models were used).

Novel environments: SDMs projections uncertainties (see section above) and non analog climate conditions (i.e. identified and quantified through species-level MESS maps) were all illustrated on the main Figure through two different hatching styles. See Supplementary Figure 4 and Supplementary Figure 5.

Supplementary Note 6

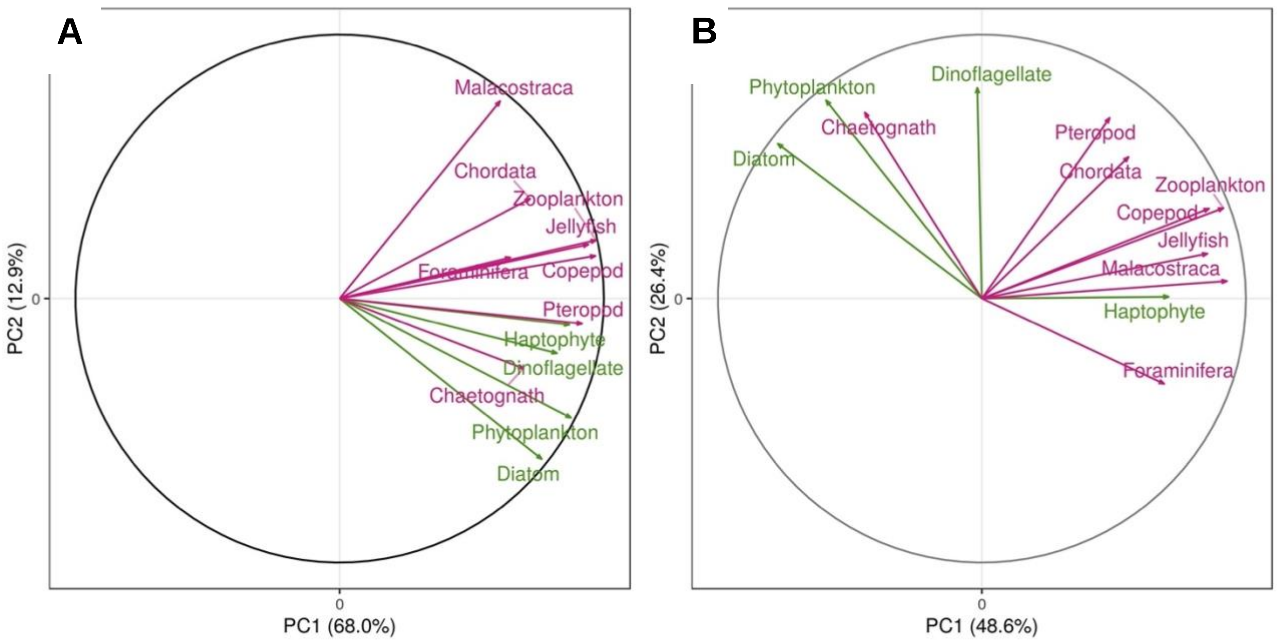

Supplementary Note 6-1: Similarity in phytoplankton (green) and zooplankton (magenta) functional groups projections of (A) global contemporary ensemble mean annual species richness (SR), and (B) difference between future and contemporary ensemble SR estimated through a Principal Component Analysis (PCA). Each vector represents the projection of a plankton functional group (PFG) projection in a reduced 2D space summarizing the main modes of SR spatial variations. The length of a vector indicates the strength of its projection along the two principal components (PC). The angle between two vectors indicates the strength and direction of the covariance between the two variables: a 90° angle indicates orthogonal vectors and thus an absence of covariance whereas a 180° angle indicates a strong negative covariance. The percentage of variance explained by each PC is reported. The PFG species richness projections were scaled to variance units before performing the PCA.

On (A) all PFGs score with PC1 > 0 as they all display a global latitudinal diversity gradient in mean annual SR (i.e. increase in SR from the poles towards the equator). Differences between phyto- and zooplankton PFGs appear on PC2 as phytoplankton score with PC2 < 0 while the zooplankton score with PC2 > 0. This discrepancy in PC space stems from the spatial separation of the SR maxima: the SR of phytoplankton PFGs peaks near the equator and in tropical upwellings whereas the SR of zooplankton PFGs (except Chaetognaths) peaks in the subtropics.

On (B) differences between and within phyto- and zooplankton PFGs are clearer: zooplankton PFGs (and Haptophytes) score with PC1 > 0 whereas phytoplankton groups (and Chaetognaths) score with PC1 < 0 and are projected on PC2 > 0 more strongly. PFGs scoring with PC1 > 0 are those presenting decreasing SR in the tropics and increasing SR at higher latitudes (~40-50°N). PFGs scoring with PC1 < 0 and PC2 > 0 are those presenting increased SR in the tropics and temperature latitudes and slighter decrease in SR near the poles. Dinoflagellates do not score with PC1 because they display decreasing SR in tropical upwellings country to Diatoms and

Chaetognaths. Foraminifera is the only group slightly scoring with PC2 < 0 because it displays stronger decrease in SR than other PFGs.

The PFGs constituting our projections of phyto- and zooplankton SR here show distinguishable mean annual SR spatial patterns in the contemporary and future ocean. This supports our assumption that phytoplankton and zooplankton can be separated into PFGs whose diversity responds to environmental variations in different ways.

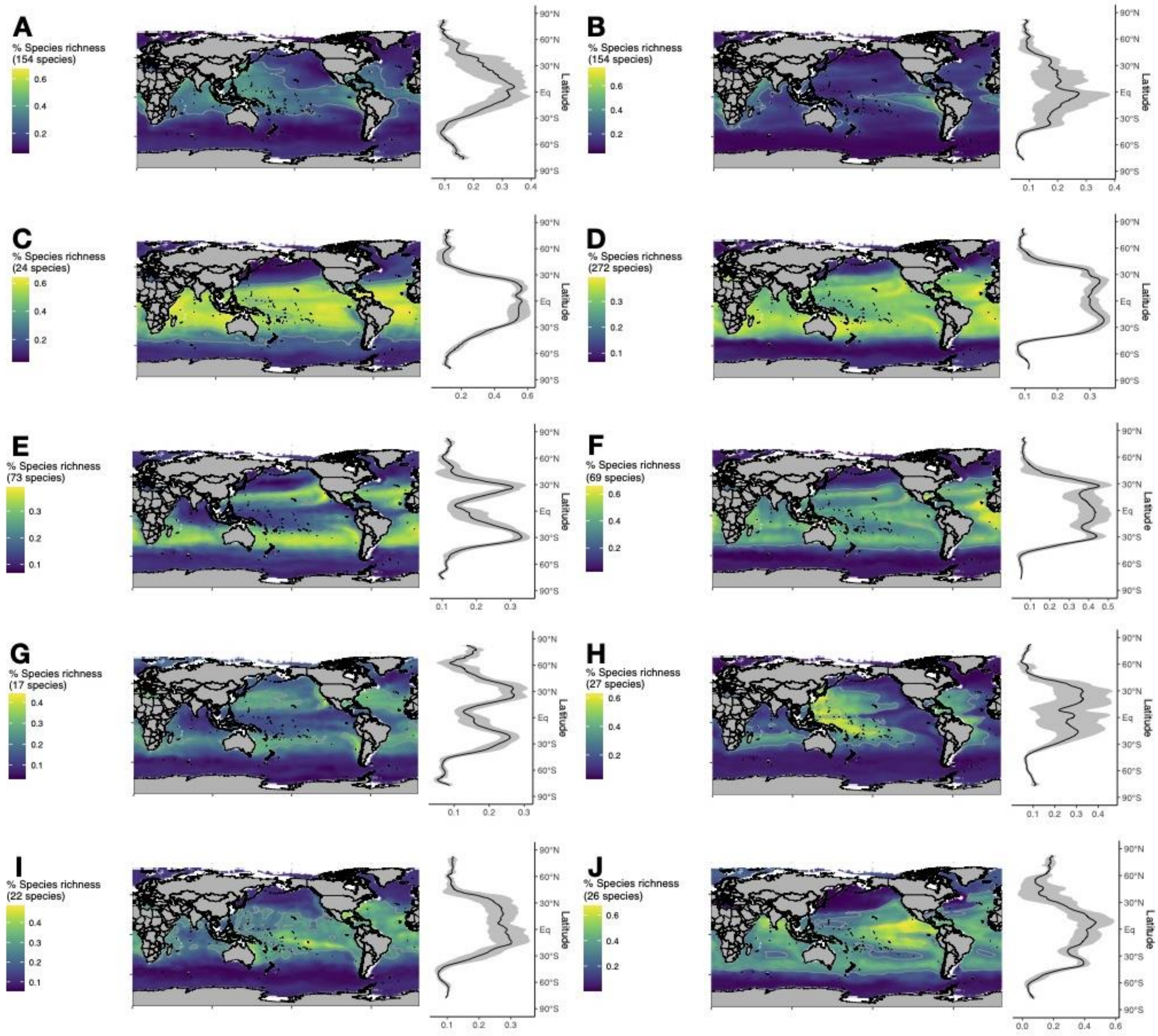

Supplementary Note 6-2: Global latitudinal diversity gradients (LDGs) in mean annual species richness (expressed in % of species modelled) for each of the 10 plankton functional groups (PFGs) investigated: (A) Diatoms, (B) Dinoflagellates, (C) Haptophytes, (D) Copepods, (E) Malacostraca, (F) Jellyfish, (G) Chordates, (H) Chaetognaths, (I) Pteropods, and (J) Foraminifera.

The PFGs patterns shown here are those used in the PCA of Supplementary Note 6-1A. The grey ribbons on the latitudinal plots illustrate the standard deviation

(i.e. longitudinal variability) associated with the average % species richness (i.e. bold lines).

These LDGs were used to further validate our ensemble projections of mean annual phyto- and zooplankton diversity by comparing them to previously published plankton groups LDG that were based on observations. Please note that direct global comparisons to previous studies are made difficult because: (i) previous diversity estimates are often based on single sampling cruises and thus do not span the same spatio-temporal scales as our estimates (e.g. single point measurements vs. 1°x1° monthly estimates); (ii) previous cruises estimate plankton alpha diversity through approaches that are quite different from ours (e.g. high throughput sequencing of environmental DNA; sum of observed taxa accounting for their relative abundances); (iii) the global LDG of some of the PFG have simply not been documented from observations (e.g., Dinoflagellates, Jellyfish, Chordates) which emphasizes the importance of our study. Therefore, it is important to keep in mind that we are here looking for similarities in overall latitudinal patterns. Below, we cite the studies that were used to validate our PFG diversity patterns and briefly highlight the elements that validate our mean annual estimates while discussing discrepancies.

- **Diatoms**:
    - ♦ Ibarbalz et al. [8] - Fig. 2A (« Protists (P) »). The authors also found a peak in species richness near the equator and a decrease towards poles. Their diversity estimates were based on molecular taxonomic units (MOTUs) derived from DNA sequencing from seawater sampled at 189 stations (TARA Oceans cruises). But see figures below for a further comparison between our results to those of Ibarbalz et al. [8].
    - ♦ Busseni et al. [9] – Figs. 4 & 5. The authors also found Diatom richness to peak near the equator and identify tropical upwelling regions as hotpots of Diatom richness. Authors also found lower diversity towards tropical gyres but find Diatom richness to increase more strongly towards the poles. Their Diatom richness estimates were obtained by merging of morphology-based estimates and MOTUs-based estimates (125 stations; TARA Oceans expedition).
    - ♦ Olguín Salinas et al. [10] – Fig. 3. A smaller scale study that documented latitudinal gradients in Diatom species richness between the Southwestern Atlantic Ocean and the Weddell Sea (30°S-60°S; estimates based on seawater samples and microscopic counts of taxonomic species). The authors also found Diatom richness to decrease from 30°S to 45°S but then to increase again from 45°S to 60°S. This provides further support to our projections of mean annual Diatom richness which increase from 40°S to > 60°S.
- **Dinoflagellates**: no previous study; closest estimate is the LDG of “photosynthetic/mixotrophic (P) Protists” from Ibarbalz et al. [8] – Fig. 2A (same as for Diatoms). The authors also found a peak in species richness near the equator and decrease towards poles.
- **Haptophyta** (mainly Coccolithophores): O’Brien et al. [11] – Fig. 3C & Fig. 6A. The authors modelled global Coccolithophores species richness from in situ observations and monthly climatologies through neural networks. The authors

found mean annual Coccolithophores richness to peak in the tropics and decrease towards high latitudes, with notable decreases in richness near tropical upwelling regions, which is very similar to our mean annual estimates.

- **Copepods**:
  - ♦ Rombouts et al. [12] – Fig. 7. The authors also found Copepod species richness to increase from the poles to the equator with peaks around 20°-30° latitude. The authors found Copepod richness to peak in the subtropical Atlantic Ocean likely because of the overrepresentation of this basin in their dataset (their Fig. 1) and the inclusion of sea surface salinity as one of the main predictors of Copepod diversity (but see Supplementary Note 4 of our study).
  - ♦ Beaugrand et al. [13] – Figs. 1 & 2. The authors projected zooplankton diversity to peak in the subtropics as a result of the selection of species based on their relative thermal tolerances. Their modelling approach was validated against the observations of Rombouts et al. [12]. The authors also found zooplankton diversity to decrease >23°C.
  - ♦ Hirai et al. [14] – Figs. 2 & 6. The authors also found Copepod species diversity (i.e. estimated from OTUs sampled across the Pacific Ocean) to decrease from the equatorial Pacific Ocean to the Arctic Ocean, with a peak in the subtropical North Pacific.
- **Malacostraca** (mainly Euphausiids): Tittensor et al. [1] - Fig. 1L. The authors also found Euphausiids species richness to decrease from the equator to the poles with peaks ~30° latitude. Overall, and despite the much lower spatial resolution of their estimates (880km), both our studies found very similar hotspots in Euphausiid diversity and a decrease in diversity at the equator.
- **Jellyfish** (holoplanktonic Cnidaria+Ctenophora): no previous study for this PFG.
- **Chordates** (Salps, Doliolids and Larvaceans): no previous study for this PFG.
- **Chaetognaths**: Miyamoto et al. [15] – Figs. 3 & 4B & 7A. The authors also found Chaetognatha species richness (estimated from species counts in the Indo-Pacific) to decrease from tropics to high latitudes, with peaks in the western equatorial Pacific and west of Australia. The authors also found zooplankton richness to decrease > 23°C. However, their observed patterns in Chaetognatha richness differs from ours in the Indian Ocean. This may be linked to the differences of our approaches but also by the high seasonality in Chaetognatha diversity they documented for this basin (Fig. 7).
- **Pteropods**: Burridge et al. [16] – Figs. 2C & 4B. Based on a single transect through the Atlantic Ocean that spanned 40°N-40°S. The authors also found Pteropod species richness to be higher between 30°S-30°N with no clear peak, and also found Pteropod richness to decrease towards 40° latitude.
- **Foraminifera** (*here it should be kept in mind that previous studies mainly estimated Foraminifera species diversity based on the top sections of sediment cores, whereas we chose to focus on occurrences obtained from net tows within the surface layers; this greatly reduced the quantity of data available and makes direct comparison difficult to interpret*):

- Rutherford et al. [17] – Figs. 1 & 2. The authors also found Foraminifera species richness to decrease from the equator to the pole with peaks in the subtropics, whereas we found Foraminifera richness to peak near the equator (i.e. in the tropical western Pacific) though with substantial longitudinal variations. However, the authors also found zooplankton richness to decrease with SST beyond 23°C.
- Tittensor et al. [1] - Fig. 1M. The authors heavily relied on the data of Rutherford et al. [17] so they found similar Foraminifera richness patterns.
- Yasuhara et al. [18] – Fig. 2. The authors also found Foraminifera species richness to decrease from the tropical Atlantic Ocean to the Arctic Ocean. Plus, the authors also found zooplankton diversity to decrease with temperature above 23°C.

Supplementary Note 6-3: Comparison of the present ensemble projections of contemporary and future changes in mean annual phytoplankton and copepod species richness (SR) to the contemporary and future changes in mean annual species diversity estimates (Shannon index, H') of Ibarbalz et al. [8].

We were able to directly compare their model projections of copepods and photosynthetic-mixotrophic protists (labelled as "Protists (P)" in Ibarbalz et al. [8]) species diversity to our results. These two groups provide the most comparable projections to our present phytoplankton (Figure 1) and copepod (Supplementary Note 6-2 above) diversity (i.e. mean annual species richness) estimates. In short, the following pairs of fields were compared:

- Contemporary mean annual phytoplankton species richness (i.e. from ensembles of habitat suitability; Fig. 1d) versus their contemporary (1996-2006) estimate of Protists (P) species diversity (i.e. from Shannon index H' based on molecular measurements; their Fig. S12A).
- Contemporary mean annual copepod species richness (Supplementary Note 6-2) versus their contemporary (1996-2006) estimate of copepod species diversity (i.e. from Shannon index H' based on molecular measurements; their Fig. S12A).
- Ensemble % Difference in mean annual phytoplankton species richness (2081-2100 minus 2012-2031) versus their anomalies of Protists (P) species diversity (2090-2099 minus 1996-2006; their Fig. S12B).
- Ensemble % Difference in mean annual copepod species richness (2081-2100 minus 2012-2031) versus their anomalies of copepod species diversity (2090-2099 minus 1996-2006; their Fig. S12B).

Before a direct comparison could be made, we normalized both ours and their estimates of contemporary phytoplankton and copepod diversity by their respective maximum values. For each pair of variables, bivariate plots were drawn with our own model estimates on the y axes and Spearman rank correlation coefficients (rho) were computed. This way we evaluate the similarity of the spatial patterns in diversity between the results of Ibarbalz et al. [8] and ours. Then, using the bivariate plots illustrating the amplitude of the future differences in diversity, we identified the regions were our projections agree or disagree on the sign of the response of protist/copepod diversity to future climate changes. Plus, by drawing the 1:1 line of these two plots, we also identified the regions where our changes in diversity are predicted to be larger or weaker than those of Ibarbalz et al. [8], relative to the respective contemporary conditions. All the plots and the results from the correlation analyses are summarized below.

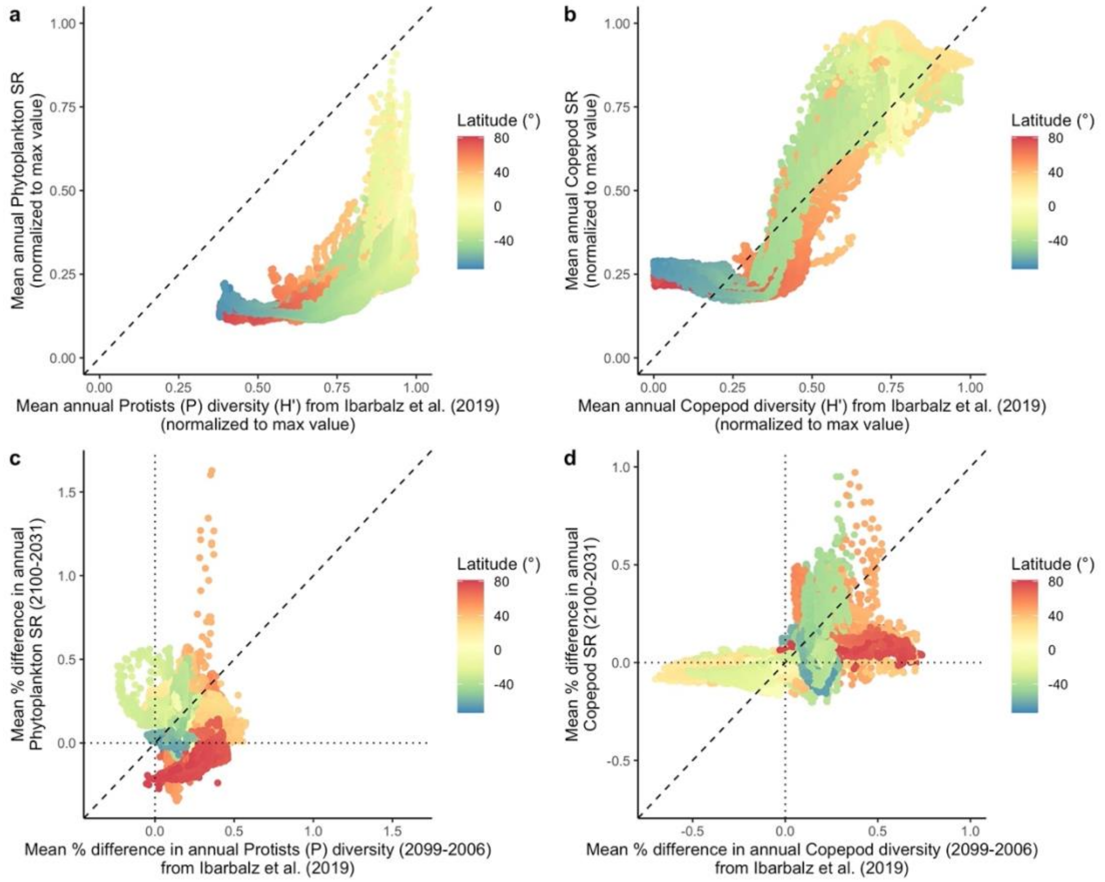

Supplementary Note 6-4: Comparison between our present estimates (always on the y axis) to those of Ibarbalz et al. [8] (always on the x axis) for (a) contemporary mean annual phytoplankton/ Protists (P) species diversity (species richness SR vs. Shannon diversity index H') and (c) mean difference (future decade – contemporary decade) in mean annual phytoplankton/ Protists (P) species diversity. (b) same as (a) and (d) same as (c) but for copepod species diversity instead of phytoplankton/ Protists (P). Each point corresponds to a 1°x1° grid cell and was colored as a function of its latitude. The dashed line represents the 1:1 line, and the dotted lines show where changes in species diversity are equal to zero.

Results from the Spearman' rank correlation tests associated to the data plotted above:

a. Rho = 0.852 ; p-value < 2.2 10^{-16} ; S = 8.019 10^{-11}
b. Rho = 0.838 ; p-value < 2.2 10^{-16} ; S = 8.748 10^{-11}
c. Rho = 0.248 ; p-value < 2.2 10^{-16} ; S = 4.064 10^{-12}
d. Rho = 0.663 ; p-value < 2.2 10^{-16} ; S = 8.821 10^{-11}

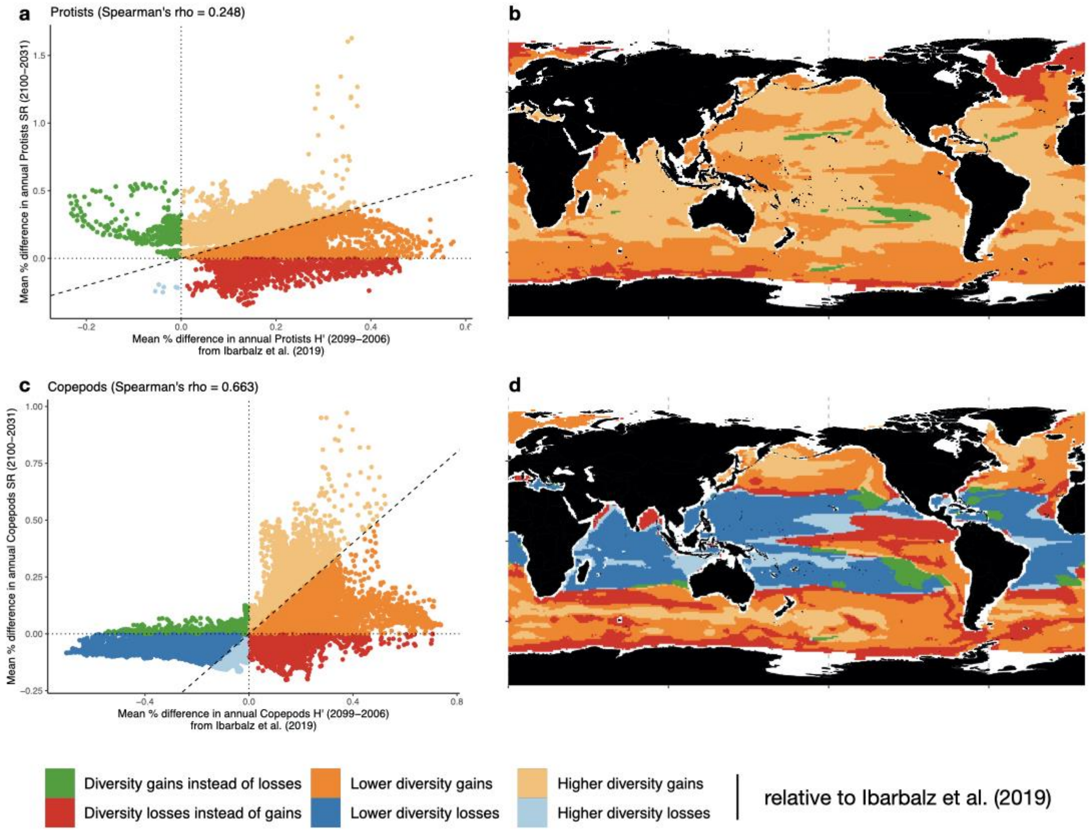

Supplementary Note 6-5: Comparison between our present estimates to those of Ibarbalz et al. [8] for (a)-(b) mean difference (future - contemporary) in mean annual phytoplankton/ Protists (P) species diversity. (c)-(d) same as (a)-(b) but for copepods. Plots (a) and (c) are the same as plots (c) and (d) in the Figure above but the points were colored according to the state of agreement between our projections and those of Ibarbalz et al. [8] as well as the relative amplitude of the projected differences in species diversity when both estimates agree on the direction of change. Cells in red and green thus correspond to those regions where our projections disagree with those of Ibarbalz et al. [8].

In short, we find significant correlations coefficients for four pairs of variables which indicates that our results are overall in line with those of Ibarbalz et al. [8]. Yet, the correlations vary in strength. Mean annual Protists (P)/phytoplankton and copepod species diversity display a similar patterns between the two studies (all rho > 0.83), indicating that both studies find very similar latitudinal diversity gradients of species diversity for Protists (P)/phytoplankton and copepods for the contemporary ocean. Although the correlation coefficient is relatively weak (rho = 0.248), both studies find Protists (P)/phytoplankton diversity to increase in the future, but they strongly disagree on the response of diversity in high latitudes of the northern hemisphere (increase vs. decrease in our case). The predicted future changes in global copepod species diversity are more similar between the two studies compared to phytoplankton,

although Ibarbalz et al. [8] found more regions where copepod diversity is likely to increase in the future.

We would like to underline that there are several major methodological differences, which makes it difficult to pinpoint the reasons behind the differences shown above. Indeed, a key issue besides the broader spatio-temporal coverage of our data, is the way plankton diversity is measured and modelled. Ibarbalz et al. [8] derived Shannon diversity indices (H') from numbers of reads of operational taxonomic units (OTUs) obtained from high throughput 16S and 18S RNA sequencing. Therefore, it is hard to evaluate the similarity of the phytoplankton (or the "Protists (P)") community they sample compared to ours. They directly estimate species diversity at 189 sampling stations and then model it as a response variable through GAMs. Then, the authors use the latter GAMs to project species diversity in space and time as a function of sea surface temperature (SST) and chlorophyll a only. Meanwhile, we estimate species diversity as a property that emerges from stacking several hundreds of different species for which we can reliably model the global habitat suitability patterns. Furthermore, we use a broader range of model types and complexity whereas Ibarbalz et al. [8] rely on GAMs only. Therefore, our respective approaches differ in a multitude of ways: (i) our biological observations span much broader spatial and temporal scales (several decades and all ocean basins vs. two cruises); (ii) species diversity is directly measured and modelled in their case contrary to our approach; (iii) we use a broader range of statistical models that better covers the commonly used range of algorithms and thus actually accounts for this major source of uncertainty in diversity forecasts; (iv) they rely on two environmental predictors (SST and chlorophyll a) to model the diversity of all the plankton groups whereas we made sure to use four different sets of predictors that span several niche dimensions adapted to each trophic level; (v) the baseline and end-of-century periods defined to compute the future environmental fields on which the statistical models are projected on differ too (10 years in their case; 20 in ours); and (vi) the Earth System Models used are not the same between our studies.

Supplementary Note 7: Analyzing the effect of occurrence rarefaction (i.e. evening sampling effort across latitudes) on the projections of zooplankton species richness.

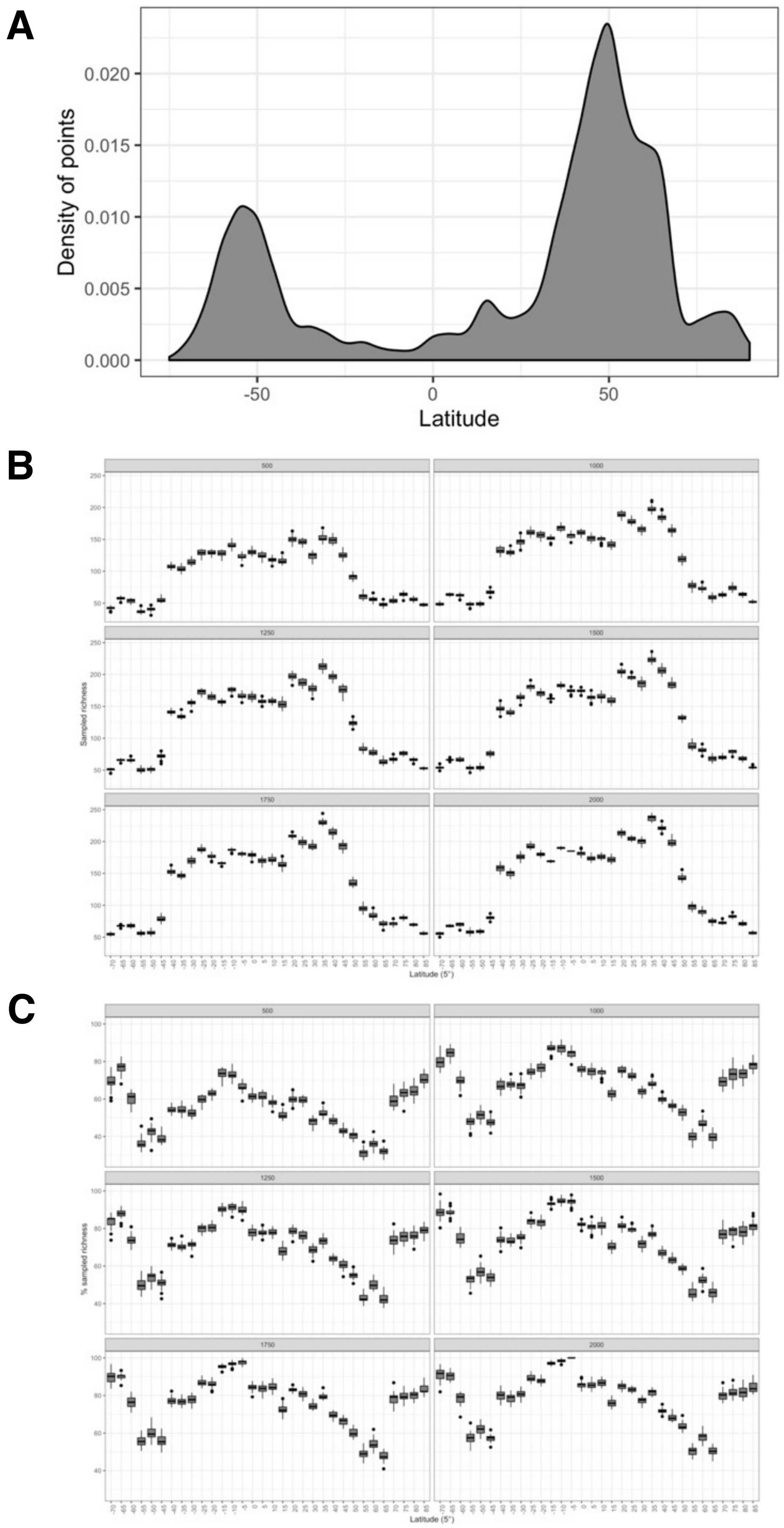

Supplementary Note 7-1: Latitudinal distribution of (A) occurrence density in the zooplankton dataset (Supplementary Figure 1). The threshold used to rarefy the zooplankton occurrence dataset was equal to the minimum density of occurrences found near the equator (here, near the 10°S band). Within latitudinal bands (5° here, but the results were tested for 2° bands), n occurrences were randomly selected 30 times and for six increasingly higher values of N: 500, 1000, 1250, 1500, 1750, 2000. These values were chosen because the threshold of minimum occurrences oscillated between ~500 and ~2000 when gathering occurrences within latitudinal bands of 2° or 5°. The (B) distribution of the total number of species richness sampled for every values of N and 5° latitudinal band was examined, as well as the (C) corresponding % of species richness sampled relative to the species richness found in each latitudinal band of the unrarefied dataset. The final N chosen was 2000 because it was the value enabling to sample the highest % of species richness relative to the unrarefied dataset, while presenting a latitudinal distribution of rarefied species richness similar to those obtained with lower N. As visible in the Supplementary Note 7-2 below, the two 5° latitudinal bands located at the polar extremes (i.e. 75°S and 90°N) presented even lower sampling effort than the equatorial bands, so the observations falling within those were simply added after the rarefaction process.

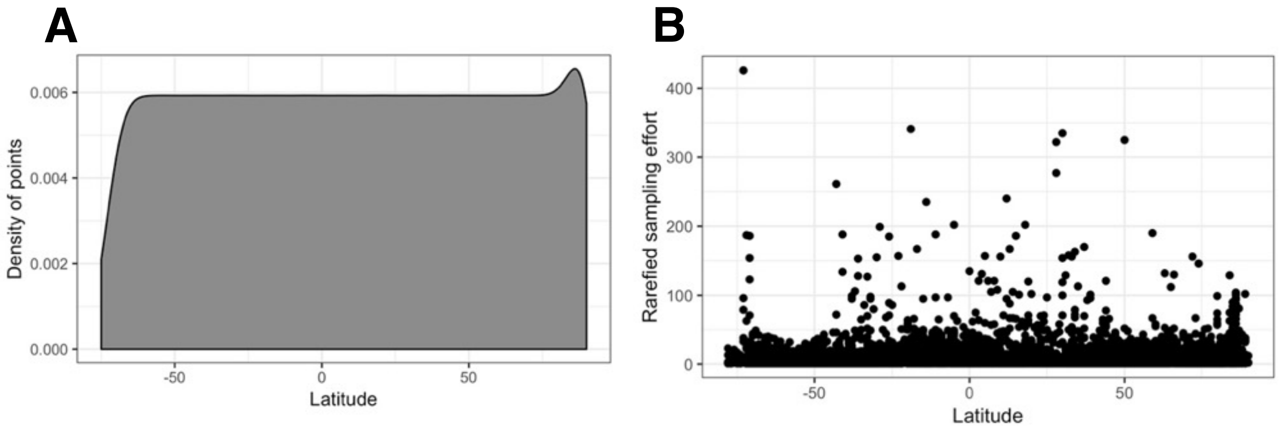

Supplementary Note 7-2: Latitudinal distribution of (A) occurrence density in the zooplankton dataset after rarefying the occurrences per 5° latitudinal bands, and (B) the corresponding number of occurrences per 1°x1° grid cell. All zooplankton species with >50 occurrences in the rarefied dataset (n = 257) were considered for species distribution modeling.

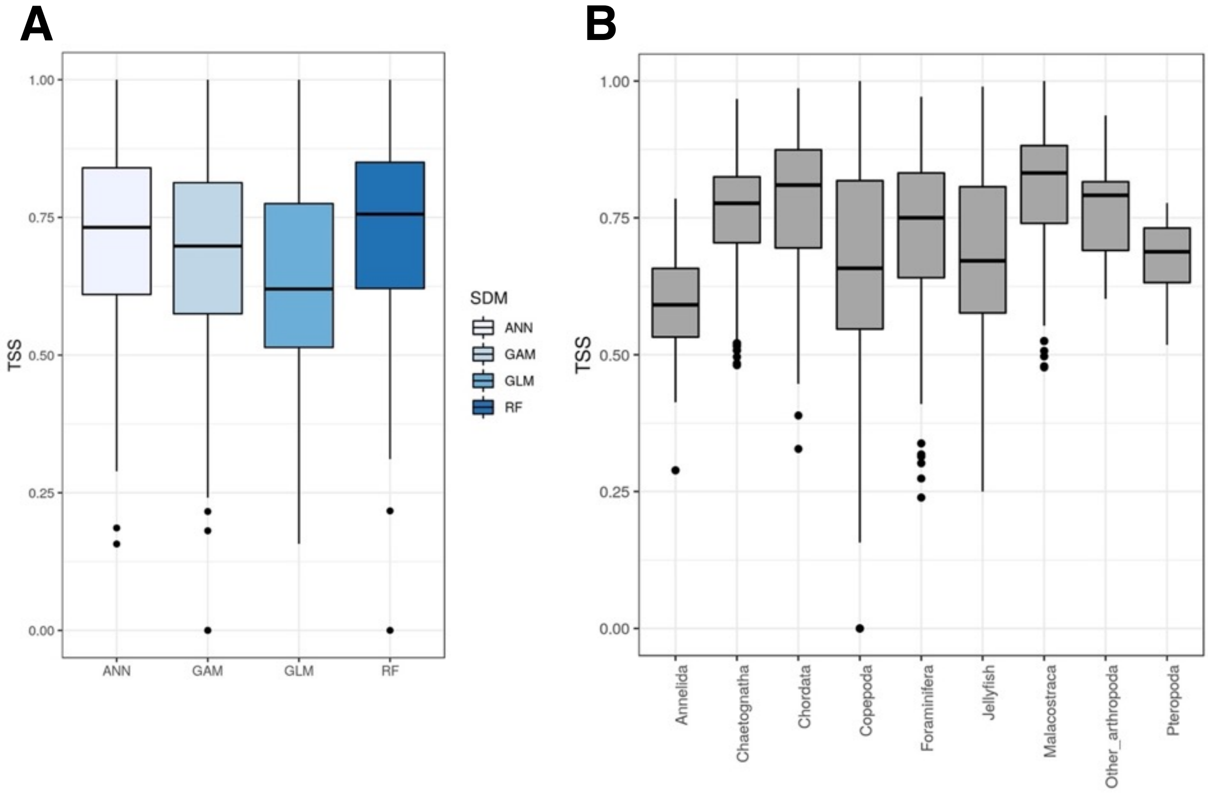

Supplementary Note 7-3: Distribution of True Skill Statistics (TSS) for the zooplankton species (n = 257) modeled based on the rarefied occurrence dataset across (A) the four types of species distributions models (SDMs) developed: Generalized Linear Models (GLM), Generalized Additive Models (GAM), Artificial Neural Networks (ANN) and classification Random Forest (RF); and (B) the main zooplankton taxonomic groups represented. The overall mean TSS was 0.698±0.16. None of the species modelled showed an average TSS value < 0.37. The predictors used for the SDMs here were: SST, dSST, dO2, logChl and logNO3. A total-background data approach was adopted again from the rarefied dataset to model the species distributions. The monthly and annual mean species richness generated by those SDMs are shown below. The lower, middle and upper boundaries of all the boxplots correspond to the 25th, 50th and 75th percentiles. The lower and upper whiskers extend from the hinges to the lowest or largest value no further than 1.5*IQR (inter-quartile range) from the lower and upper hinges. In (A), the sample size of each SDM-specific boxplot is N = 1285 (257 species modelled x 5 cross evaluation runs). In (B), the sample size of each boxplot (N species modelled x 4 SDMs x 5 cross evaluation runs) is: N = 40 for Annelida, N = 480 for Chaetognatha, N = 100 for Chordata, N = 3320 for Copepoda, N = 380 for Foraminifera, N = 340 for Jellyfish, N = 380 for Malacostraca, N = 20 for Other Arthropoda, and N = 80 for Pteropoda.

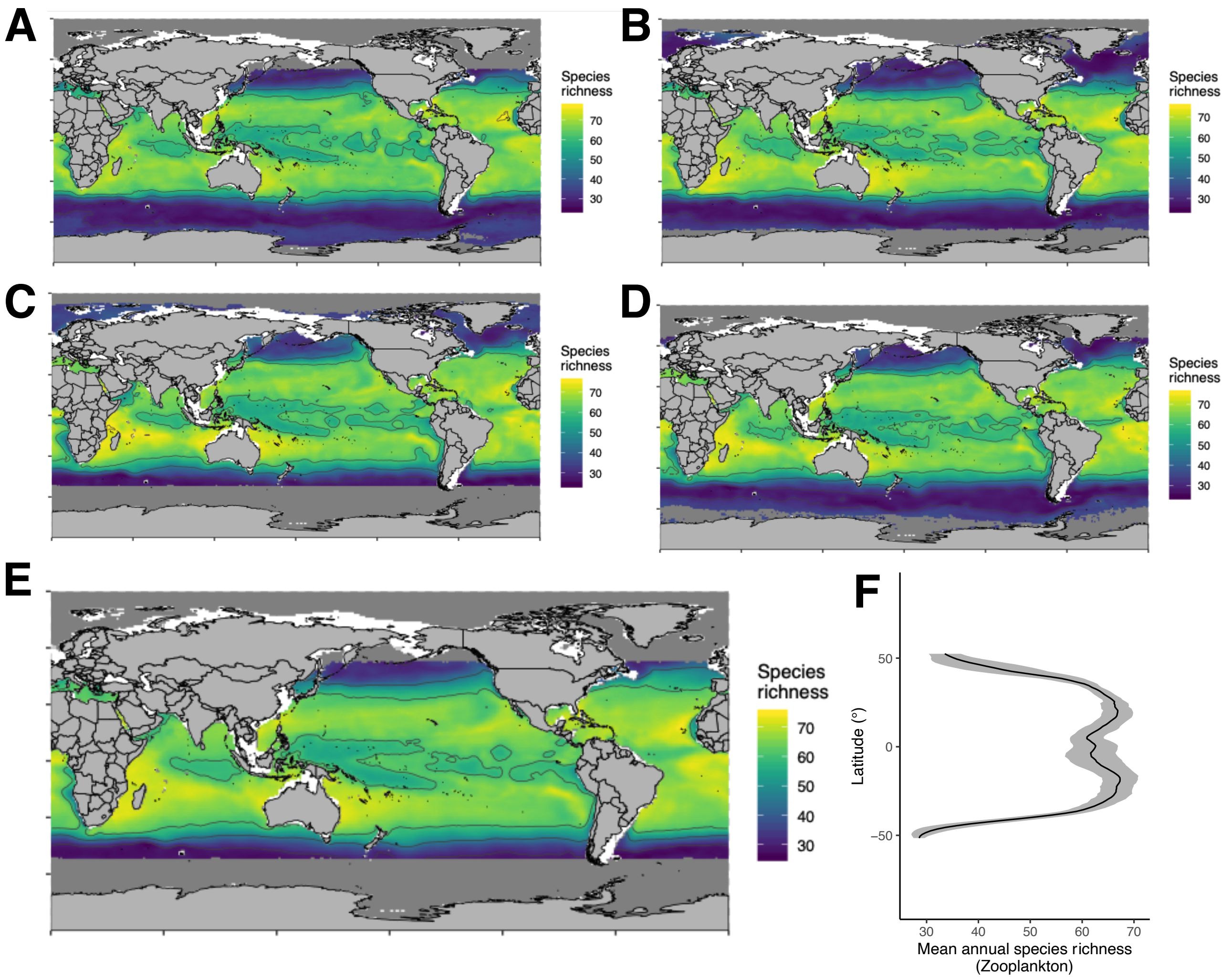

Supplementary Note 7-4: Maps of the global zooplankton species richness patterns obtained from the species distribution models (SDMs) trained from the rarefied dataset of occurrences and projected on the monthly climatological conditions of the four months depicting seasonal variations: (A) January, (B) April, (C) July and (D) October. The four monthly maps were combined to obtain an estimate of (E) annual zooplankton species richness and (F) its corresponding latitudinal zonal mean species richness. Estimates of monthly and annual species richness were obtained the same way as for the main projections (see Methods). The missing values at high latitudes correspond to the regions where satellite observations of surface chlorophyll-a (i.e. logChl) are unavailable. The grey contour in (F) illustrates the standard deviation (std) associated with the zonal average displayed by the bold line.

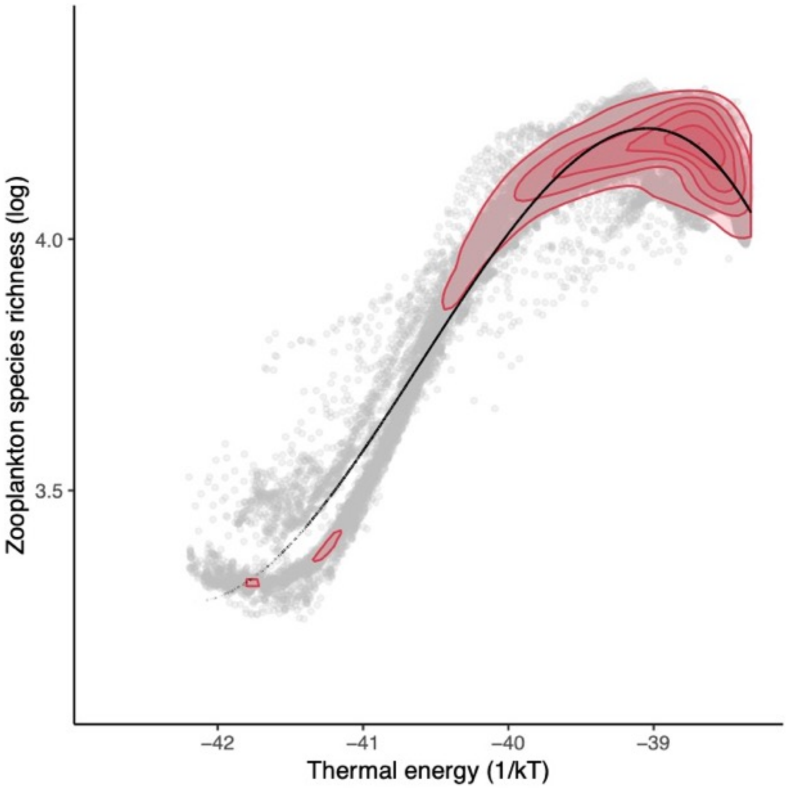

Supplementary Note 7-5: The modelled relationship between mean annual zooplankton species richness and temperature (as shown in Figure 2) obtained from the rarefied dataset of zooplankton occurrences. The modelled species richness estimates are lower than those obtained from the unrarefied dataset, as expected from the necessarily lower number of zooplankton species modelled (~50%). Yet, the diversity-thermal energy relationship observed is very similar to the one obtained from the full dataset. As temperature is the main driver of the modelled diversity patterns, this strongly suggests that our main results are not an artefact of sampling biases and that the emergent decrease of zooplankton diversity towards the highest temperatures is not linked to the lower sampling effort observed near the equator. The solid curve illustrates the 3rd degree polynomial fit that best explains the variation in log(Zooplankton SR) as a function of mean annual available thermal energy. The colored isopleths illustrate the density of ocean grid cells, based on two dimensional kernel density estimates, and were used to highlight the parts of the gradients driving the observed non-linear relationships.

Supplementary Note 8: Extended description of Figure 2 (analysis of the relationships between phyto- and zooplankton mean annual species richness under the framework of the Metabolic Theory of Ecology, MTE).

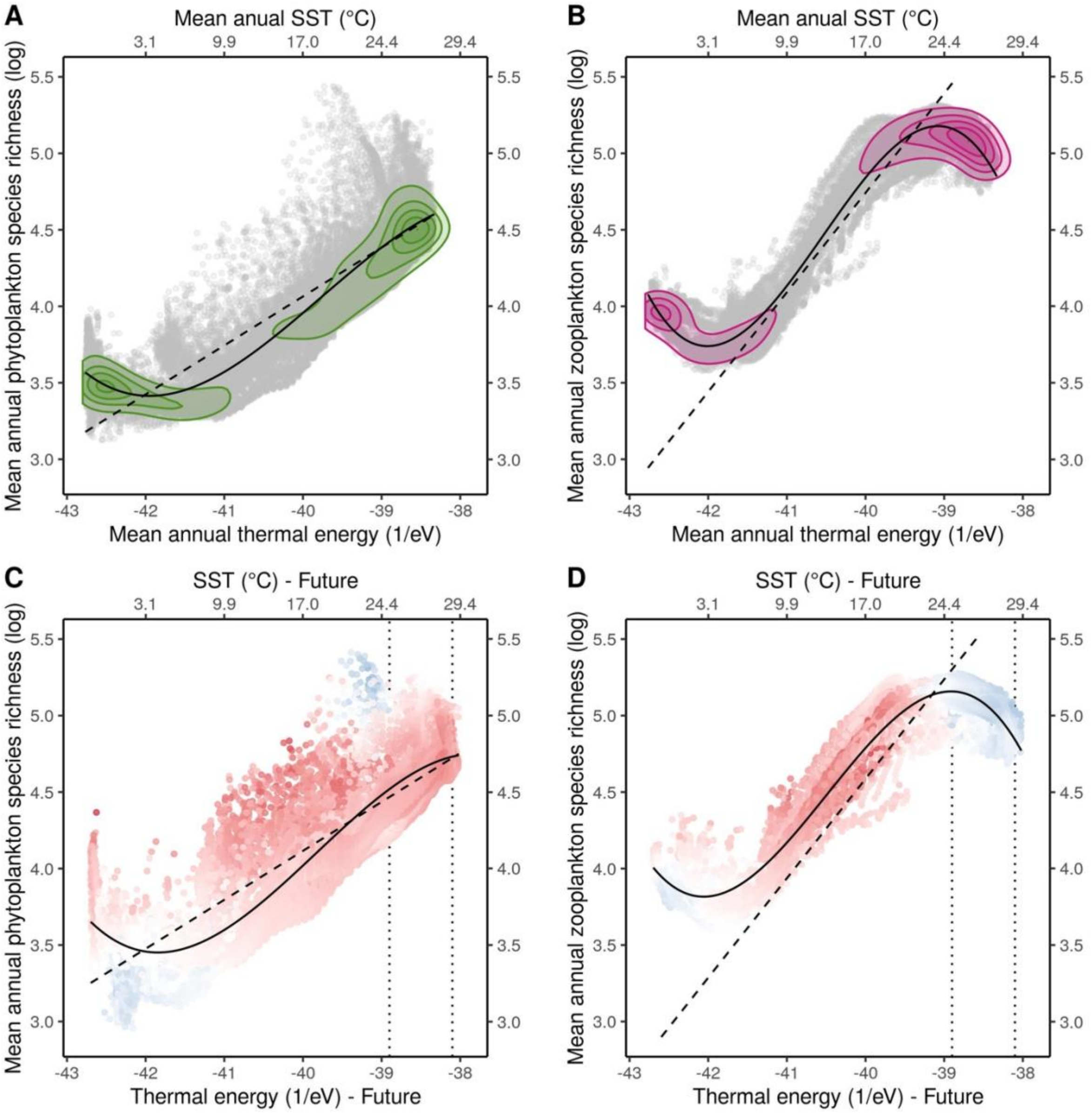

Supplementary Note 8: Relationships between logged mean annual (A) and (C) phytoplankton and (B) and (D) zooplankton species richness (SR) and mean annual surface available thermal energy (eV^{-1}) in (A) and (B) the contemporary ocean and in (C) and (D) the future ocean, examined following the framework of the Metabolic Theory of Ecology (MTE). The mean thermal energy was inferred from the mean annual sea surface temperature (SST, in Kelvin) and Boltzmann's constant (k). The dashed lines illustrate the global linear relationship predicted from the slopes expected

from the MTE (~0.32 for phytoplankton; ~0.65 for zooplankton). The solid curves illustrate the 3rd degree polynomial fit that best explains the global variations of log(SR) as a function of mean annual available thermal energy. The colored isopleths in A,B) indicate the density of ocean grid cells based on 2D kernel density estimates and highlight the parts of the thermal gradient driving the observed nonlinear relationships. The vertical dotted lines indicate the range of SST prevailing in the tropical band (i.e. latitudes < 30°) for the end-of-century period, according to the ensemble of earth system models forced by a RCP8.5 greenhouse gas emission scenario. In C,D) the points were colored as a function of the ensemble projection of the difference in mean annual SR between the future and the contemporary ocean. This way we highlight how future warming leads to an increase in phytoplankton SR and a decrease in zooplankton SR in the future tropical ocean.

Following the MTE framework, we examined the relationship between the logged annual species richness (SR) and available thermal energy to better understand why phyto- and zooplankton SR respond differently to climate change in the tropical ocean. The SR of both groups displays non-monotonic relationships with thermal energy that depart from the linear slope predicted by the MTE (~|0.32| for phytoplankton; ~|0.65| for zooplankton). For both groups, different modes of SR variations are modelled across the thermal energy gradient as illustrated by the polynomial fits. First, a mode of decreasing SR is found from the coldest temperatures (-1.8°C) to approximately 10.5°C, with slope values steeper than -0.13 for phytoplankton ($R^2 = 0.11$; $p < 0.001$), and -0.31 for zooplankton ($R^2 = 0.60$; $p < 0.001$) where mean annual sea surface temperature (SST) is < 5°C. This decrease in SR is not predicted by the Metabolic Theory of Ecology (MTE) and can be ascribed to the strong environmental seasonality prevailing in such temperate regimes because it prevents the establishment of a high phyto- and zooplankton SR on the annual scale, as supported by relatively high rates of month-to-month species turn-over (Supplementary Figure 6). Second, a mode of steep SR increase was found to start ~11°C above which the fitted linear slopes are closer to the predictions of the MTE. For phytoplankton, the linear slopes fitted from 11°C to increasingly higher thermal energies range between 0.33-0.40 until ~23°C ($R^2 = 0.16$-0.58; all $p < 0.001$), after which a third mode of weaker SR increase takes over, as evidenced by slopes < 0.29. The linear slope best matching the MTE predictions is the one fitted for oceans regions where mean annual SST is > 22°C (slope = 0.33, $R^2 = 0.164$; $p < 0.001$). For zooplankton, the linear slopes fitted from 11°C to 13-19°C exceed the slope predicted by the MTE (all slopes range between 0.68-1.00; $R^2 = 0.31$-0.77; all $p < 0.001$). The best fit to the MTE was found for the regions where mean annual SST is comprised between 11°C-22°C (slope = 0.66; $R^2 = 0.78$; $p < 0.001$). Beyond a maximum SST of 22°C, the linear slopes fitted from 11°C become lower than MTE predictions as zooplankton SR clearly starts decreasing towards highest temperatures. This pattern of decreasing diversity at high temperatures has been reported for other animal clades. This third mode of decreasing zooplankton SR does not match the corresponding

mode found for phytoplankton where SR keeps increasing. Such different arrangements of SR to the warmest range of the thermal gradient explain why SR peaks are not co-located for phyto- and zooplankton and why their responses differ in the tropics (Figure 1). In regions where climate change will create conditions of mean annual SST >25°C, zooplankton SR is predicted to decrease abruptly because the novel temperature regime might exceed the thermal tolerances of many species, which lowers habitat suitability. Meanwhile, phytoplankton SR increases nearly linearly when SST is >11°C, and is thus promoted by future warming and very high temperatures remain suitable for most phytoplankton taxa.

Supplementary Note 9

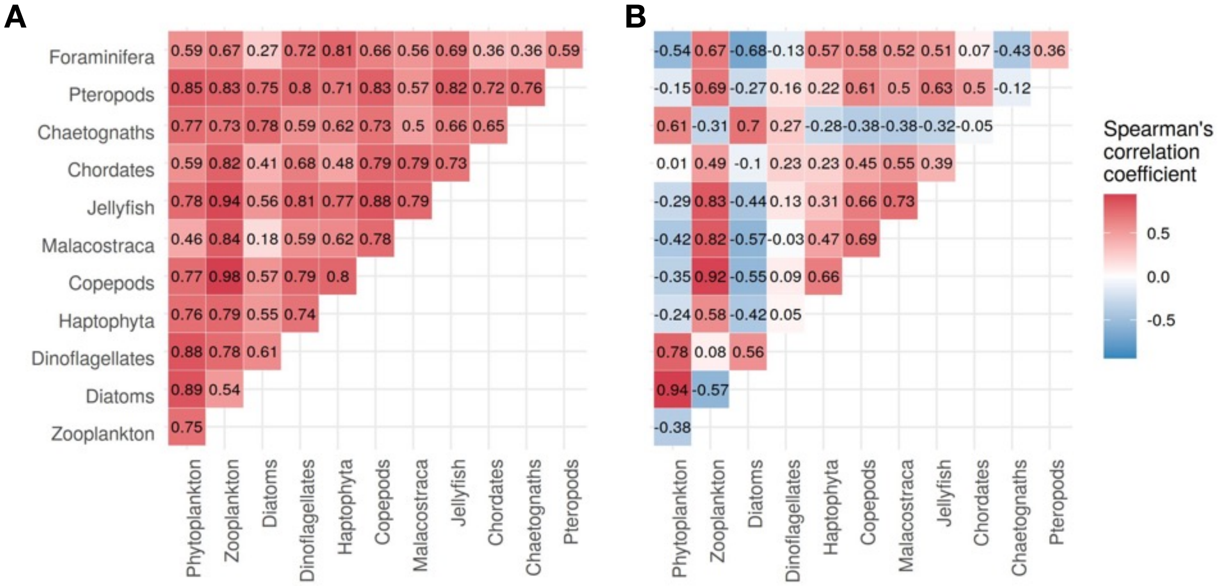

Supplementary Note 9-1: Heatmaps showing the Spearman's rank correlation coefficients computed between the (A) mean annual baseline species richness and (B) the % difference in mean annual species richness (future-baseline) of phyto- and zooplankton as well as their ten main Plankton Functional Groups (PFGs). Total sample size is N = 35023.

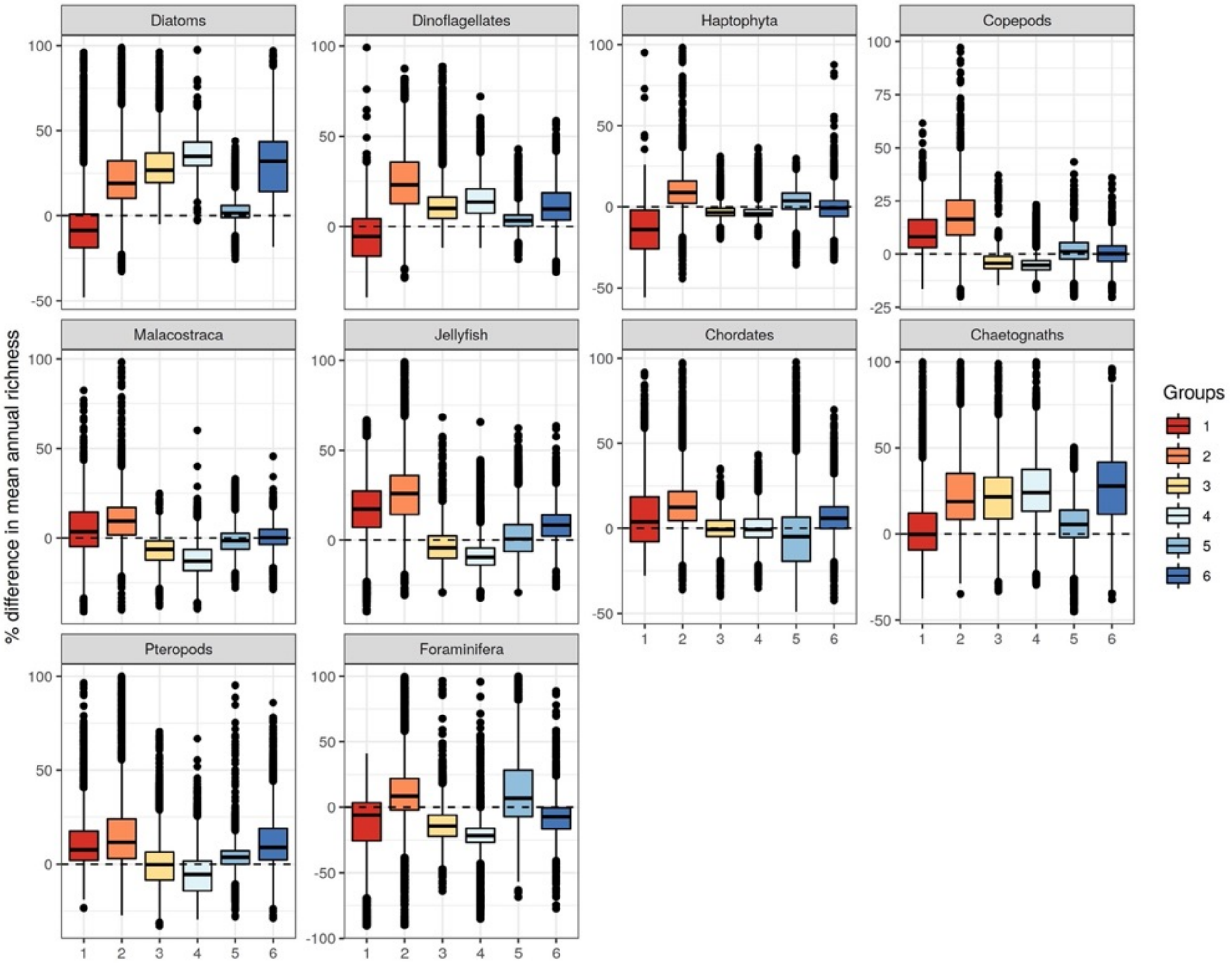

Supplementary Note 9-2: Boxplots showing the distribution of the % difference in mean annual species richness (future-baseline) between the six regions of climate impacts severity for the ten main Plankton Functional Groups (PFGs) studied. The lower, middle and upper boundaries of all the boxplots correspond to the 25th, 50th and 75th percentiles. The lower and upper whiskers extend from the hinges to the lowest or largest value no further than 1.5*IQR (inter-quartile range) from the lower and upper hinges. N = 35023 grid cells for the total sample size (Nregion1 = 2344; Nregion2 = 5954; Nregion3 = 6748; Nregion4 = 8290; Nregion5 = 7637; Nregion6 = 4050).

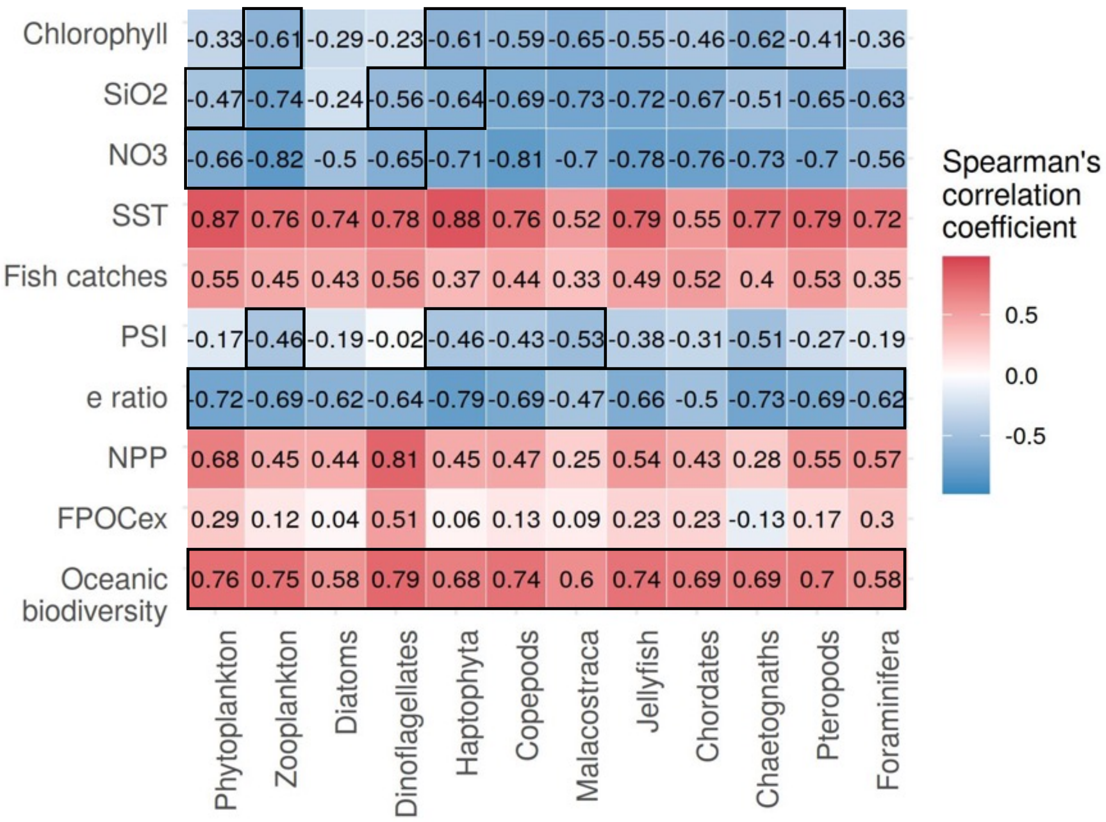

Supplementary Note 9-3: Heatmap showing the Spearman's rank correlation coefficients (rho) computed between the proxy variables of ecosystem services (ES), a few key environmental covariates and the mean annual baseline species richness (SR) of phyto- and zooplankton, and their ten main Plankton Functional Groups (PFGs). Total sample size is N = 35023.

While we cannot infer mechanistic links based on our modelling approach and from the data at hand, we use the present correlation analysis to assess the strength of associations between the PFGs SR and the proxy variables of ES, on an annual scale. This way, we can test relevant hypotheses that link plankton diversity to ecosystem functioning. Because we rely on non parametric rank correlation coefficients that can detect weak signals as significant, we carefully highlighted with black outlines the strongest correlation coefficients (rho > I0.4I) that we deem interesting for our study as

they support potentially robust Biodiversity-ES relationships. All correlations displaying a rho > |0.1| tested as significant (p-value < 0.01).

Please note that the variable used here represent the mean annual conditions of the contemporary offshore global ocean. Oceanic biodiversity quantifies the species richness of higher trophic levels (tunas, sharks, mammals, squids) [1]. FPOCex (mg Carbon m^{-2} day^{-1}) quantifies the amount of particulate organic matter exported beyond the euphoric zone [3]. NPP (mg Carbon m^{-2} day^{-1}) quantifies the rate at which biomass is stored in the phytoplankton and made available to grazers [3]. The e ratio is the FPOCex to NPP ratio and thus quantifies the efficiency of the biological carbon pump in exporting carbon out of the euphotic zone [3]. PSI is an index of surface plankton particles size derived the from the slope of the plankton particles spectrum [4]. Fish catches (tons km^{-2} $year^{-1}$) estimates the reported and unreported catch rates of small (< 30cm) pelagic fishes over the 1990-2019 period [2]. NO3 and SiO2 (μM) quantify surface macronutrients concentrations (nitrates and silicates respectively). SST (°C) corresponds to sea surface temperature. Chlorophyll (mg C m^{-3}) was used as a proxy of surface phytoplankton biomass. Chlorophyll, NO3, SiO2, Fish catches, NPP and FPOCex were log transformed.

Our analysis shows that the SR of PFGs covaries positively with the SR of higher trophic levels, meaning they might respond to similar drivers (i.e. temperature, productivity) in the same way. This also brings further support to the validity of our projections as our models do seem to capture the main processes that drive marine biodiversity. We also find that the efficiency of the biological carbon pump decreases with the SR of all PFG, which supports the view that species-rich and functionally diverse communities better retain the biomass produced within surface layers while species-poor communities tend to leak higher rates of biomass out of the surface ocean. This can also be mediated through re-arrangement of community size structure as species rich communities are composed of more numerous smaller species (but see Supplementary Notes 10 & 11). This is supported by the fact that plankton size (i.e. PSI) shows similar correlation patterns to the e-ratio. Haptophyta (i.e. Coccolithophores) SR decreases with plankton size but also silicates. Plus, we evidenced that Haptophyta SR strongly decreases where Diatoms SR peaks (see Supplementary Note 6). This could stem from our models partly capturing competitive effects leading to the dominance of Diatoms over Coccolithophores under conditions of silicates replenishment, though this is hard to evaluate at the scale of our study. The ensuing decrease/increase in Coccolithophores/Diatoms habitat suitability could help explain why plankton side distribution shifts towards larger particles (microphytoplankton). Such competitive effects could also help explain why Diatoms SR and Haptophyta SR display such contrasting responses to future climate change (Supplementary Note 9-2 above). Zooplankton SR decreases with plankton particles size, suggesting that higher densities of large phytoplankton cells might not favour the diversity of grazing PFGs (i.e. Copepods and Malacostraca) as only a fraction of their taxa may be able to graze upon larger cells due to size-based limitations in prey capture. Similarly, we find zooplankton SR to decrease with phytoplankton biomass (i.e. Chlorophyll). This implies that higher food availability may lead to a decrease in SR through competitive exclusion, instead of promoting niche partitioning within the grazing zooplankton. Ultimately, we find phytoplankton SR to decrease with higher nutrients availability this can be interpreted by two mutually non-exclusive hypotheses: (i) species rich communities draw down nutrients concentrations more efficiently, meaning phytoplankton diversity optimizes nutrients use

efficiency in the ecosystem which would translate into less biomass/energy leaving the surface ecosystem (corroborated by negative correlations between phytoplankton SR and e-ratio); (ii) less seasonally varying and warmer and nutrients-poor ecosystems (e.g. tropical gyres) sustain species-rich communities as competition under very low nutrients availability leads to niche partitioning that enables the co-existence of many taxa, potentially characterized by diverse functional traits. Under either hypotheses, our results support the existence of important links between key ecosystem functions (i.e. nutrients use efficiency and carbon export) and plankton species richness, and warrants further studies examining at the relationships between ES, species richness, community traits expression and functional diversity.

Supplementary Note 10

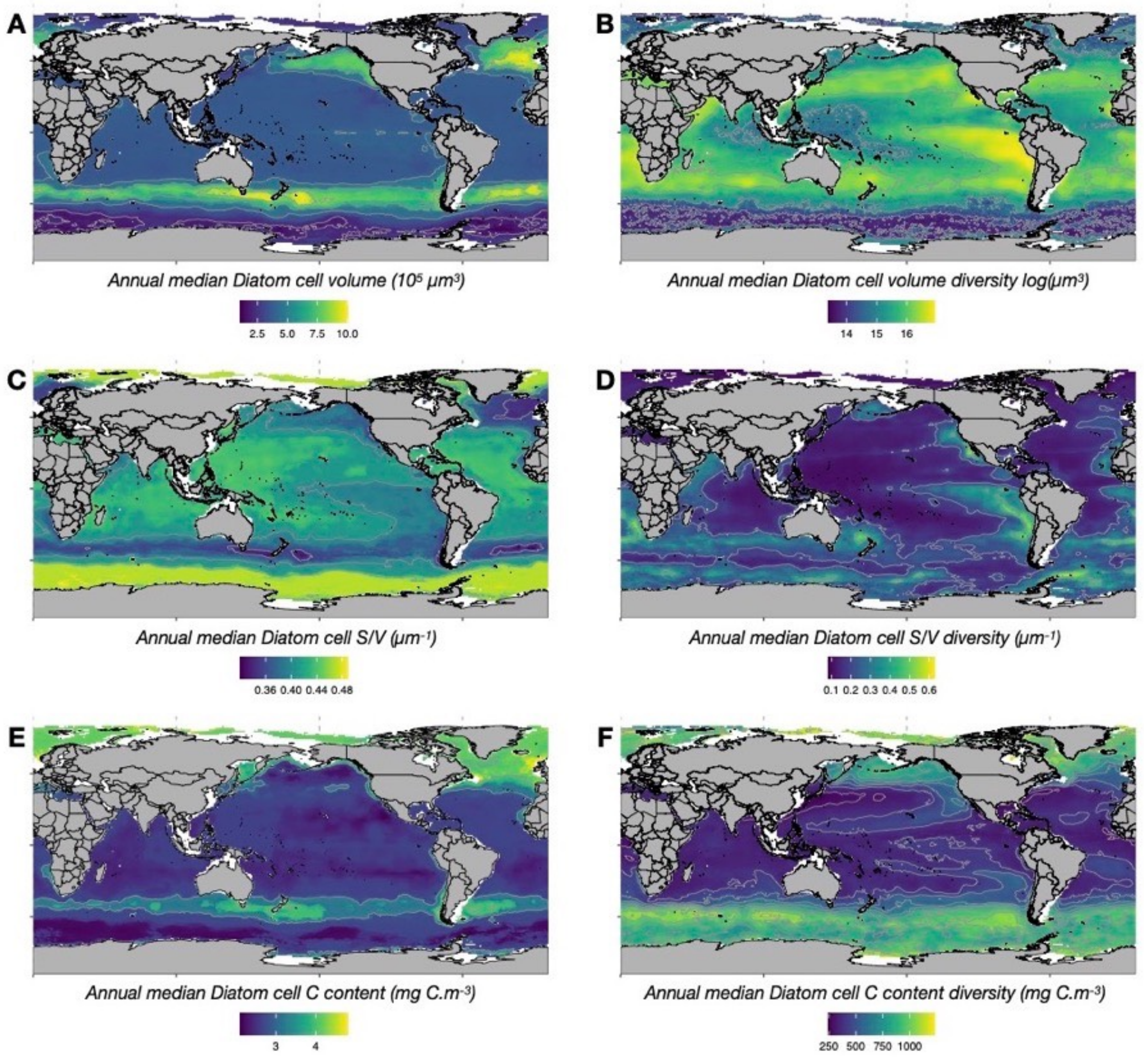

Supplementary Note 10-1: Patterns of annual Diatom community size structure in the contemporary global surface ocean, estimated through (A) median cell volume, (B) median cell volume diversity, (C) median cell surface to volume ratio (S/V), (D) median cell S/V diversity, (E) median cell Carbon content, and (F) median cell Carbon content diversity.

The monthly projections of Diatom species habitat suitability (HSI) from each model member (n = 16) were combined with species-level estimates of average cell size measurements issued from Leblanc et al. [19] to estimate monthly HSI-weighted median Diatoms cell volume, S/V and C content. The diversity of Diatoms cell volume, S/V and C content was estimated through the corresponding HSI-weighted variance. Each monthly model member estimate was then used to derive an annual median estimate for all these six variables aimed to characterize the various dimensions of the Diatom community size structure emerging from our HSI projections. By doing so, we aim to: (i) evaluate if our model estimates of Diatom community composition provide realistic microphytoplankton size patterns (Supplementary Note 10-2 below); (ii) link our projections of phytoplankton species richness with the provision of key ecosystem services in the contemporary global ocean (Supplementary Note 10-3 below; Supplementary Note 9); and (iii) test whether

future climate change will favour Diatom species exhibiting smaller volumes, larger S/V ratios and lower C content (Supplementary Note 10-4 below).

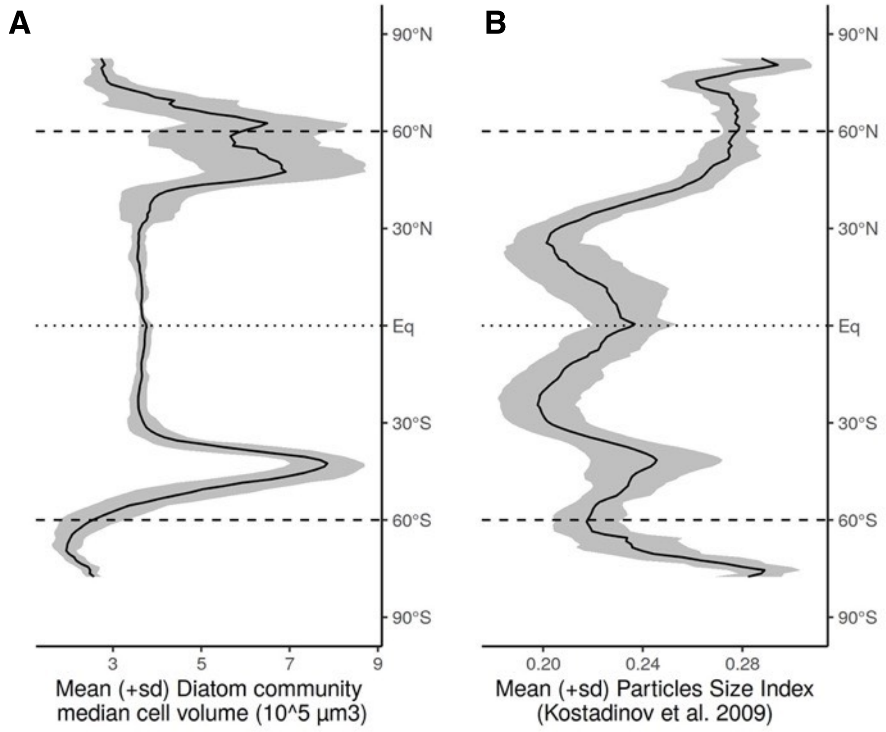

Supplementary Note 10-2: Average latitudinal patterns in (A) annual median surface Diatom median cell volume and (B) annual mean Particles Size Index (PSI) derived from the slope of satellite-based distribution of plankton size.

Here, we assess whether our estimate of Diatom community median cell volume reflects the global latitudinal pattern in plankton particles size observed from space. Higher values of Diatom community median cell volume are expected at latitudes where higher densities of larger plankton cells (i.e. microphytoplankton) occur. Our approach is based on monthly species-level habitat suitability projections (not cell concentrations) for a small subset (~150 Diatom species) of the whole natural phytoplankton community which comprises all size classes (pico-, nano- and microphytoplankton). Therefore, a direct comparison with the satellite models outputs from Kostadinov et al. [4] should be interpreted with caution, as those are based on a plankton particle size distributions that include the whole phytoplankton size spectrum. Consequently, we only aim to assess whether our community composition estimates are able to reproduce the known pattern of increasing microphytoplankton size and carbon content with latitude. We acknowledge our present approach might underestimate phytoplankton size gradients that emerge from interactions within the whole phytoplankton size spectrum.

Interestingly, our approach is able to reproduce the pattern of increasing cell size with latitude, with peaks in subpolar latitudes (~45° latitude). However, we are unable to capture the increase in phytoplankton size observed at the equator which is driven by the

presence of equatorial upwelling systems. Similarly, we estimate a decrease in annual Diatom median cell volume towards the poles which contradicts the satellite observations. In order to remain confident and conservative in our approach, we thus exclude the latitudes beyond >60°S and °N (dashed lines on the zonal plots) for our next analyses.

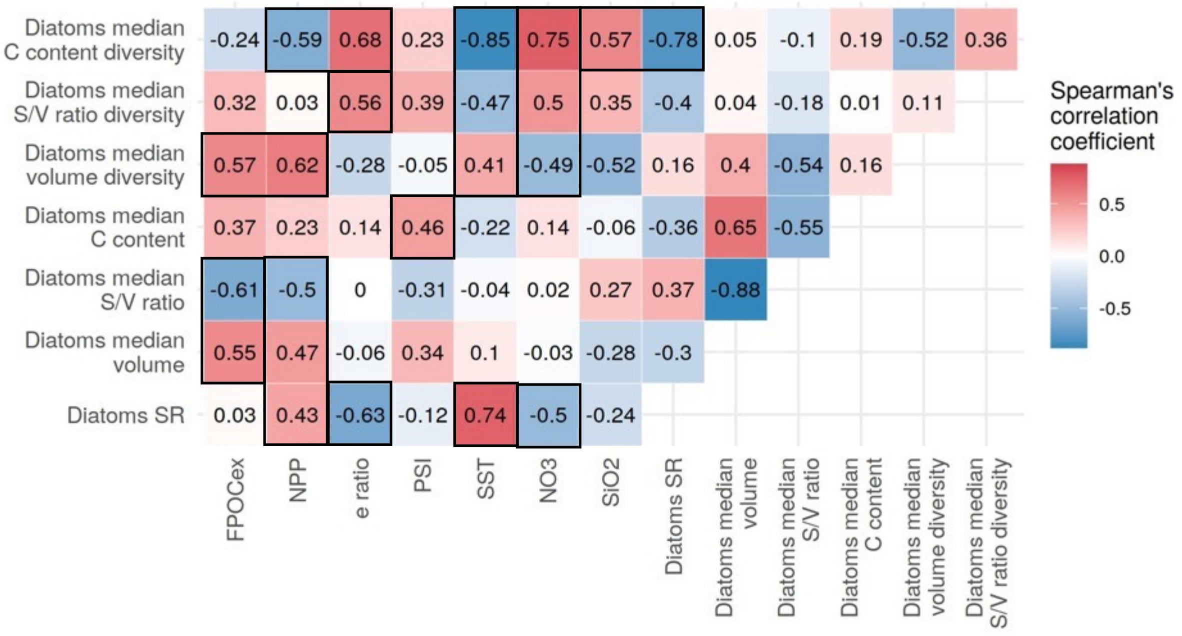

Supplementary Note 10-3: Heatmap of Spearman rank correlation coefficients (rho) computed between the proxy variables of ecosystem services (ES), a few key environmental covariates and the estimates of annual surface Diatom community size structure (Supplementary Note 10-1) and Diatom species richness (SR). Poles (>60° latitude) were removed for this analysis (Supplementary Note 10-2).

While we cannot infer mechanistic links based on our modelling approach and from the data at hand, we use the present correlation analysis to assess the strength of associations between Diatom community size structure and the proxy variables of ES. This way, we test relevant hypotheses that link phytoplankton size structure to ecosystem functioning. Because we rely on non parametric rank correlation coefficients that can detect weak signals as significant, we carefully highlighted with black outlines the strongest correlation coefficients (rho > l0.4l) that we judge interesting for our study as they support potentially robust phytoplankton size-ES relationships. All correlations displaying a rho > l0.1l tested as significant (p-value < 0.01).

Please note that the variable used here represent the mean annual conditions of the contemporary offshore global ocean. Oceanic biodiversity quantifies the species richness of higher trophic levels (tunas, sharks, mammals, squids) [1]. FPOCex (mg Carbon m^{-2} day^{-1}) quantifies the amount of particulate organic matter exported beyond the euphoric zone [3]. NPP (mg Carbon m^{-2} day^{-1}) quantifies the rate at which biomass is

stored in the phytoplankton and made available to grazers [3]. The e ratio is the FPOCex to NPP ratio and thus quantifies the efficiency of the biological carbon pump in exporting carbon out of the euphotic zone [3]. PSI is an index of surface plankton particles size derived the from the slope of the plankton particles spectrum [4]. NO3 and SiO2 (μM) quantify surface macronutrients concentrations (nitrates and silicates respectively). SST (°C) corresponds to sea surface temperature. Chlorophyll (mg C m^{-3}) was used as a proxy of surface phytoplankton biomass. Chlorophyll, NO3, SiO2, NPP and FPOCex were log transformed.

We find that communities with larger Diatom cells and smaller Diatom S/V ratios covary positively with FPOCex and NPP. This supports the view that communities characterized by larger phytoplankton cells favour primary productivity and export a larger quantity of carbon outside the euphotic zone. Interestingly, NPP and FPOCex also covary positively with the variance of Diatom cell volumes (i.e. cell size diversity), implying that the range of Diatom cell sizes could also be linked to primary productivity and export. However, Diatom median cell volume and S/V do not display any relationships with the e-ratio. Rather, the e-ratio is more strongly correlated to Diatom SR and Diatom size and S/V diversity. Interestingly, this supports the view that phytoplankton size structure alone is not sufficient to explain gradients in the fraction of carbon exported into the deeper layers, and that species diversity might play an important role, probably through its effects on the expression of other functional traits (e.g. cell shape, degree cell wall silicification, coloniality, resting spores production etc.; see Tréguer et al. [20]). Therefore, our results support the view that larger microphytoplankton cells favour the quantity of energy available to grazers and of carbon exported but suggest that species diversity plays a more important role in regulating the efficiency of the biological carbon pump. This brings further support to our approach and warrants future studies investigating the impact of plankton species diversity on ecosystem functioning.

In line with Supplementary Note 9-3, we find that Diatom SR and Diatom size diversity increases with SST and decreases with macronutrients concentrations. This can be interpreted by two mutually non-exclusive hypotheses: (i) Diatom rich communities (in terms of SR but also size diversity) draw down nutrients concentrations more efficiently, implying that microphytoplankton diversity optimizes nutrients use efficiency which would translate into less biomass/energy leaving the surface ecosystem (corroborated by negative correlations between Diatom SR/ size diversity and e-ratio); (ii) warmer and nutrients-poor ecosystems (e.g. tropical gyres) sustain species-rich communities dominated by smaller cell sizes, as competition under very low nutrients availability leads to niche partitioning that enables the co-existence of many taxa, potentially characterized by diverse functional traits.

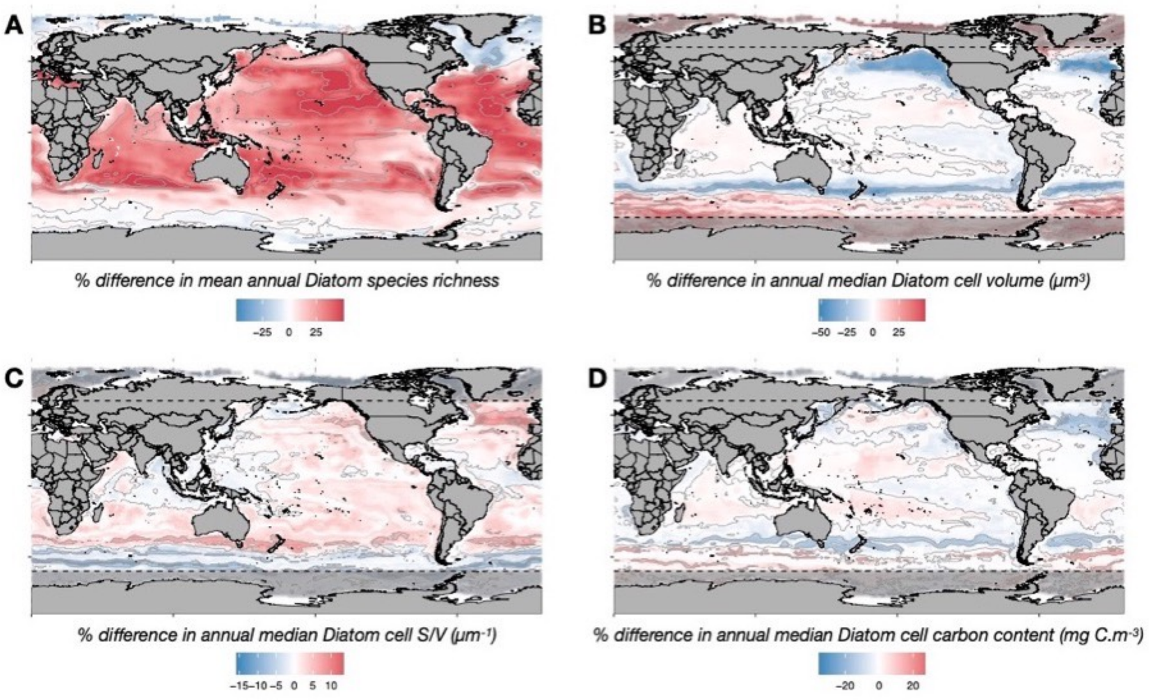

Supplementary Note 10-4: Patterns of % difference (future-baseline) in (A) mean annual Diatom species richness (SR), (B) annual median Diatom cell volume, (C) annual median Diatom cell surface to volume ratio (S/V), and (D) annual median Diatom cell Carbon content.

As in Supplementary Note 10-1, the future monthly projections of Diatom species habitat suitability (HSI) from each model member (n = 80) were combined with the species-level estimates of average cell size measurements to estimate monthly HSI-weighted median Diatoms cell volume, S/V and C content for the future surface global ocean. By looking at the % difference between the future and the baseline estimates of Diatom community size structure, we investigate how anthropogenic climate change might reshuffle the size structure of the microphytoplankton community. More specifically, we here test whether future warming will lead to a decrease in median cell size through the replacement of larger Diatom species by smaller ones, a process expected under global warming, particularly towards higher latitudes. The polar regions (>60° latitude) were shaded in grey as we acknowledge that our estimates of microphytoplankton size structure are not in line with observations there (Supplementary Note 10-2). Please note that these regions also correspond to regions where model members disagree the most reading the sign of the response of phytoplankton SR to climate changes (Figure 1).

Our results show that future warming drives an increase in Diatom SR which translates into a decrease in Diatom median cell volume and C content and an increase in Diatom median S/V, especially in temperate latitudes > 40° (region 2 in Figure 4). Our projections thus corroborate the view that future warming will promote smaller microphytoplankton cells , with larger S/V ratios, at the expense of larger ones. Combined with our results from Supplementary Note 10-3, these projections support our view that future changes in phytoplankton species diversity will affect ecosystem functioning and the provision of ecosystem services related to C cycling. We expect future increases in Diatom SR and

decreases in median cell size predicted to weaken NPP and FPOCex as well as the efficiency of the biological carbon pump, and potentially decrease the efficiency of macronutrients, in region 2 (Figure 4).

Supplementary Note 11

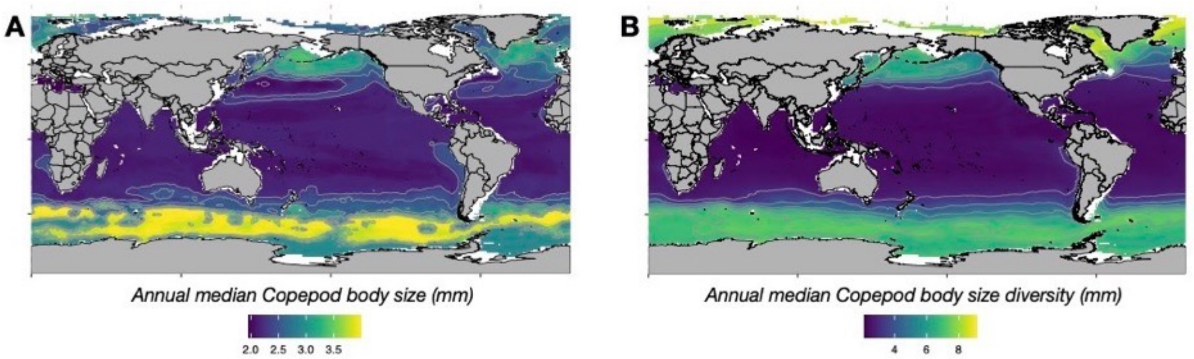

Supplementary Note 11-1: Patterns of annual Copepod community size structure in the contemporary global surface ocean, estimated through (A) median body size, (B) median body size diversity. The monthly projections of Copepod species habitat suitability (HSI) from each model member (n = 16) were combined with species-level estimates of average body size measurements issued from Razouls et al. [21] (https://copepodes.obs-banyuls.fr/en/; synthesized in Benedetti et al. [22] and Brun et al. [23]) to estimate monthly HSI-weighted median Copepod body size. Copepod body size diversity was estimated through the corresponding HSI-weighted variance. Every monthly model member estimate was then used to derive an annual median estimate these two variables that characterize the size structure of surface Copepod communities from our HSI projections.

By doing so, we aim to: (i) evaluate if our model estimates of Copepod community composition provide realistic copepod community size patterns; (ii) link our projections of zooplankton species richness with the provision of key ecosystem services in the contemporary global ocean (Supplementary Note 11-2 below; Supplementary Note 9); and (iii) test whether future climate change will favour smaller Copepod species (Supplementary Note 11-2 below).

No global satellite-based product is available to compare our estimates to zooplankton community size structure observations, we find that our estimates of global surface Copepod body size are extremely similar to the previous global estimates of Brun et al. [23], in terms of both spatial patterns and median values. Our approach is thus able to reproduce a realistic pattern of increasing Copepod body size from the equator to the poles, with increases in tropical upwelling regions (e.g. Peruvian and Benguela upwellings). Furthermore, our estimate of median Copepod body size covaries positively with plankton particles size (see Supplementary Note 11-2 below), which gives us additional confidence in our approach.

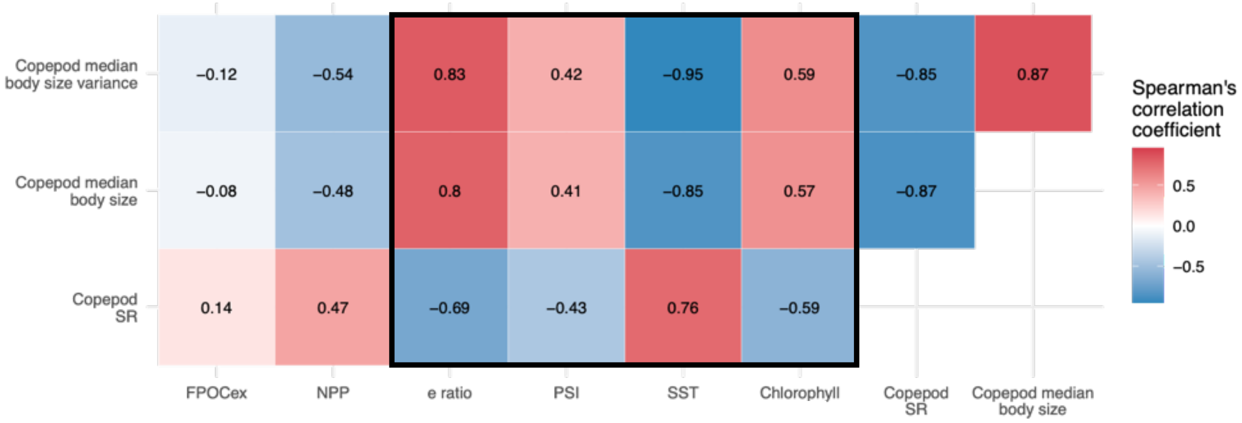

Supplementary Note 11-2: Heatmap of Spearman rank correlation coefficients (rho) computed between the proxy variables of ecosystem services (ES), a few key environmental covariates and the estimates of annual surface Copepod community size structure and Copepod species richness (SR).

While we cannot infer mechanistic links based on our modelling approach and from the data at hand, we use the present correlation analysis to assess the strength of associations between Copepod community size structure and the proxy variables of ES. This way, we test relevant hypotheses that link zooplankton size structure and species diversity to ecosystem functioning. Because we rely on non parametric rank correlation coefficients that can detect weak signals as significant, we carefully highlighted with black outlines the strongest correlation coefficients (rho > |0.4|) that we judge interesting for our study as they support potentially robust zooplankton size-ES relationships. All correlations displaying a rho > |0.1| tested as significant (p-value < 0.01).

Please note that the variable used here represent the mean annual conditions of the contemporary offshore global ocean. Oceanic biodiversity quantifies the species richness of higher trophic levels (tunas, sharks, mammals, squids) [1]. FPOCex (mg Carbon m^{-2} day^{-1}) quantifies the amount of particulate organic matter exported beyond the euphoric zone [3]. NPP (mg Carbon m^{-2} day^{-1}) quantifies the rate at which biomass is stored in the phytoplankton and made available to grazers [3]. The e ratio is the FPOCex to NPP ratio and thus quantifies the efficiency of the biological carbon pump in exporting carbon out of the euphotic zone [3]. PSI is an index of surface plankton particles size derived the from the slope of the plankton particles spectrum [5]. SST (°C) corresponds to sea surface temperature. Chlorophyll (mg C m^{-3}) was used as a proxy of surface phytoplankton biomass. Chlorophyll, NPP and FPOCex were log transformed.

Overall, we find that Copepod SR decreases with Copepod median body size and body size diversity, suggesting that communities characterized by a larger proportion of large-bodied species still comprise smaller bodied ones. Meanwhile, tropical communities are characterized by numerous smaller-bodied species and very few large-bodied species. Interestingly, Copepod SR and size structure also show contrasting correlation patterns with phytoplankton biomass (Chlorophyll), plankton particles size (PSI) and the efficiency of the biological carbon pump (e ratio). Indeed, higher phytoplankton biomass promotes lower Copepod species diversity by favoring larger-bodied species which could

outcompete smaller ones as they are able to graze on larger phytoplankton cells (e.g. corroborated by the positive correlation between Copepod median size and PSI). This supports the view that higher primary production leads to competitive exclusion (and not niche partitioning) within the zooplankton community, which in turn leads to a decrease in species diversity. However, we cannot exclude that SST also strongly influences the emerging pattern of Copepod SR through the selection of species with broader thermal tolerances [13]. Yet, both hypotheses are not mutually exclusive. Our results also suggest that Copepod size structure is a major factor in regulating the efficiency of the biological carbon pump but not the amount of carbon exported outside of the euphotic zone (i.e. no correlation with FPOCex). This can be explained by the fact that larger copepod species present many functional traits that tend to scale with body size (e.g. production of larger fecal pellets, deeper of vertical migrations, higher biomass etc. [23-25]) and that promote carbon export. Overall, this gives us further confidence that our species diversity and community composition estimates are relevant for studying ecosystem functioning and the provision of marine ecosystem services.

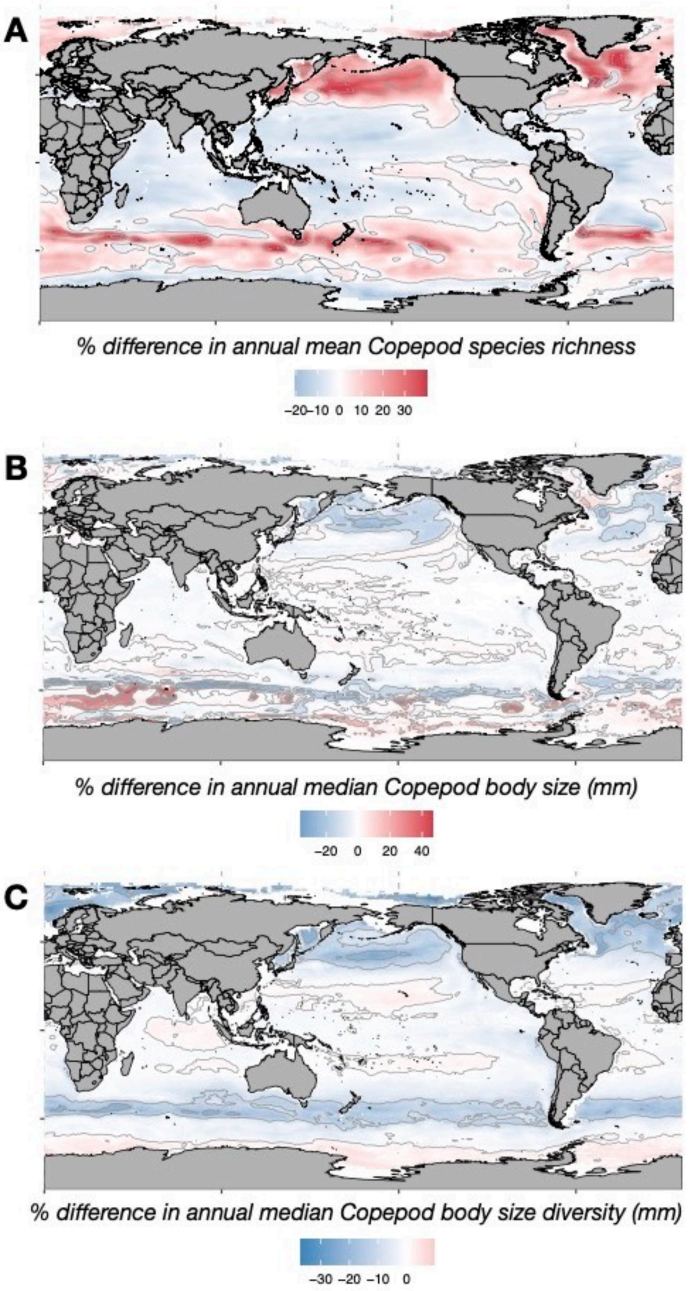

Supplementary Note 11-3: Patterns of % difference (future-baseline) in (A) mean annual Copepod species richness (SR), (B) annual median Copepod body size, and (C) annual median Copepod body size diversity.

As in Supplementary Note 11-1, the future monthly projections of Copepod species habitat suitability (HSI) from each model member (n = 80) were combined with the species-level estimates of average adult female body size measurements to estimate monthly HSI-weighted median Copepod body size for the future surface global ocean. By looking at the % difference between the future and the baseline estimates of Copepod community size structure, we investigate how anthropogenic climate change might reshuffle the size structure of the zooplankton community. More specifically, we here test whether future warming will lead to a decrease in median body size through the replacement of large-bodied Copepod species by smaller ones, a process expected under global warming, particularly towards higher latitudes [24-26].

In line with Figures 1 and 3, our ensemble projections show that anthropogenic climate change will trigger the poleward migration of tropical Copepod species which results in large increase in SR in high latitudes combined with large turn-over rates (i.e. species replacement, see Methods) as the warm-water species replace the cold-water ones (already discussed in the main text). Here, we further show that such changes in SR and community composition will also affect the size structure of the Copepod communities. In agreement with our expectations, we find that global warming will decrease the median body size of the Copepod community in high latitudes (except the Southern Ocean), which is driven by the replacement of large-bodied species by smaller ones. Median Copepod body size variance (i.e. body size diversity) follows a similar pattern: future changes in Copepod SR and composition will decrease body size diversity

Together with Supplementary Note 11-2, our results confirm our expectations that future changes in plankton diversity will alter ecosystem functioning and the efficiency of the biological carbon pump, particularly at high latitudes (regions 1 and 2 in Figure 4). Our result show that future increases in Copepod SR, and the ensuing turn-over in community composition, will decrease the median body size of the surface zooplankton community which might decrease the efficiency of the biological carbon pump (i.e. the fraction of carbon produced in the surface that is exported below the euphotic zone).

Supplementary References

1. Tittensor, D. P., Mora, C., Jetz, W., Lotze, H. K., Ricard, D., Berghe, E. V., *et al.* Global patterns and predictors of marine biodiversity across taxa. *Nature*, **466**(7310)**:** 1098-1101 (2010).
2. Watson, R. A., A database of global marine commercial, small-scale, illegal and unreported fisheries catch 1950–2014. *Scientific Data*, **4**(1)**:** 170039 (2017).
3. DeVries, T., & Weber, T. The export and fate of organic matter in the ocean: New constraints from combining satellite and oceanographic tracer observations. *Global Biogeochemical Cycles*, **31**(3)**:** 535-555 (2017).
4. Kostadinov, T., Siegel, D., Maritorena, S. Retrieval of the particle size distribution from satellite ocean color observations. *Journal of Geophysical Research: Oceans*, **114**(C9) (2009).
5. Siccha, M., Kucera, M. ForCenS, a curated database of planktonic foraminifera census counts in marine surface sediment samples. *Scientific Data*, **4:** 170109 (2017).
6. Righetti, D., Vogt, M., Gruber, N., Psomas, A., Zimmermann, N. E. Global pattern of phytoplankton diversity driven by temperature and environmental variability. *Science Advances*, **5**(5)**:** eaau6253 (2019).
7. Righetti, D., Vogt, M., Zimmermann, N. E., Guiry, M. D., Gruber, N. PhytoBase: A global synthesis of open-ocean phytoplankton occurrences. *Earth Syst Sci Data*, **12**(2)**:** 907-933 (2020).
8. Ibarbalz, F. M., Henry, N., Brandão, M. C., Martini, S., Busseni, G., Byrne, H., *et al.* Global Trends in Marine Plankton Diversity across Kingdoms of Life. *Cell*, **179**(5)**:** 1084-1097.e1021 (2019).
9. Busseni, G., Caputi, L., Piredda, R., Fremont, P., Hay Mele, B., Campese, L., *et al.* Large scale patterns of marine diatom richness: Drivers and trends in a changing ocean. *Global Ecology and Biogeography*, **29**(11)**:** 1915-1928 (2020).
10. Olguín Salinas, H. F., Alder, V. A., Puig, A., Boltovskoy, D. Latitudinal diversity patterns of diatoms in the Southwestern Atlantic and Antarctic waters. *Journal of Plankton Research*, **37**(4)**:** 659-665 (2015).
11. O'Brien, C. J., Vogt, M., Gruber, N. Global coccolithophore diversity: Drivers and future change. *Progress in Oceanography*, **140:** 27-42 (2016).
12. Rombouts, I., Beaugrand, G., Ibanez, F., Gasparini, S., Chiba, S., Legendre, L. A multi-variate approach to large-scale variation in marine planktonic copepod diversity and its environmental correlates. *Limnology and Oceanography*, **55**(5)**:** 2219 (2010).
13. Beaugrand, G., Rombouts, I., Kirby, R. R. Towards an understanding of the pattern of biodiversity in the oceans. *Global Ecology and Biogeography*, **22**(4)**:** 440-449 (2013).
14. Hirai, J., Tachibana, A., Tsuda, A. Large-scale metabarcoding analysis of epipelagic and mesopelagic copepods in the Pacific. *PLoS ONE*, **15**(5)**:** e0233189 (2020).

15. Miyamoto, H., Kotori, M., Itoh, H., Nishida, S. Species diversity of pelagic chaetognaths in the Indo-Pacific region. *Journal of Plankton Research*, **36**(3)**:** 816-830 (2014).
16. Burridge, A. K., Goetze, E., Wall-Palmer, D., Le Double, S.L., Huisman, J., Peijnenburg, K. T. C. A. Diversity and abundance of pteropods and heteropods along a latitudinal gradient across the Atlantic Ocean. *Progress in Oceanography*, **158:** 213-223 (2017).
17. Rutherford, S., D'Hondt, S., Prell, W. Environmental controls on the geographic distribution of zooplankton diversity. *Nature*, **400**(6746)**:** 749-753 (1999).
18. Yasuhara, M., Hunt, G., Dowsett, H. J., Robinson, M. M., Stoll, D. K. Latitudinal species diversity gradient of marine zooplankton for the last three million years. *Ecology Letters*, **15**(10)**:** 1174-1179 (2012).
19. Leblanc, K., Arístegui, J., Armand, L., Assmy, P., Beker, B., Bode, A.*, et al.* A global diatom database – abundance, biovolume and biomass in the world ocean. *Earth Syst Sci Data*, **4**(1)**:** 149-165 (2012).
20. Tréguer, P., Bowler, C., Moriceau, B., Dutkiewicz, S., Gehlen, M., Aumont, O.*, et al.* Influence of diatom diversity on the ocean biological carbon pump. *Nature Geoscience*, **11**(1)**:** 27-37 (2018).
21. Razouls, C., Desreumaux, N., Kouwenberg, J., de Bovée F. Biodiversité des Copépodes planctoniques marins (morphologie, répartition géographique et données biologiques). Sorbonne Université, CNRS. Disponible sur http://copepodes.obs-banyuls.fr (2005-2021)
22. Benedetti, F., Gasparini, S., Ayata, S-D. Identifying copepod functional groups from species functional traits. *Journal of Plankton Research*, **38**(1)**:** 159-166 (2016).
23. Brun, P., Payne, M. R., Kiørboe, T. A trait database for marine copepods. *Earth Syst Sci Data*, **9**(1)**:** 99-113 (2017).
24. Brun, P., Payne, M. R., Kiørboe, T. Trait biogeography of marine copepods–an analysis across scales. *Ecology Letters*, **19**(12)**:** 1403-1413 (2016).
25. Brun, P., Stamieszkin, K., Visser, A. W., Licandro, P., Payne, M. R., Kiørboe, T. Climate change has altered zooplankton-fuelled carbon export in the North Atlantic. *Nature Ecology & Evolution*, **3**(3)**:** 416-423 (2019).
26. Beaugrand, G., Edwards, M., Legendre, L. Marine biodiversity, ecosystem functioning, and carbon cycles. *Proceedings of the National Academy of Sciences*, **107**(22)**:** 10120-10124 (2010).
