# Supplementary material for: Major restructuring of marine plankton assemblages under global warming
Source: Nat Commun. 2021 Sep 1;12:5226. doi: 10.1038/s41467-021-25385-x (PMC8410869; doi:10.1038/s41467-021-25385-x)
Supplement: Supplementary file 3 — Description of Additional Supplementary Files [file 41467_2021_25385_MOESM3_ESM.pdf]

Description of Additional Supplementary Files

File Name: Supplementary Data 1
Description: Table summarizing the workflow of the implementation of the zooplankton occurrence dataset and highlighting the number of occurrences discarded at each main step. Those steps are described in section A.2 of the Methods.

File Name: Supplementary Data 2
Description: Scientific names and taxonomic classification of all 860 species modelled in the present study. The classification and species names nomenclature used are the ones of the World Register of Marine Species (WoRMS; https://www.marinespecies.org/).
