# Supplementary material for: Major restructuring of marine plankton assemblages under global warming
Source: Nat Commun. 2021 Sep 1;12:5226. doi: 10.1038/s41467-021-25385-x (PMC8410869; doi:10.1038/s41467-021-25385-x)
Supplement: Supplementary file 6 — Reporting Summary [file 41467_2021_25385_MOESM6_ESM.pdf]

nature portfolio

Corresponding author(s): Fabio Benedetti

Last updated by author(s): Jun 23, 2021

Reporting Summary

Nature Portfolio wishes to improve the reproducibility of the work that we publish. This form provides structure for consistency and transparency in reporting. For further information on Nature Portfolio policies, see our Editorial Policies and the Editorial Policy Checklist.

Statistics

For all statistical analyses, confirm that the following items are present in the figure legend, table legend, main text, or Methods section.

n/a	Confirmed	
☐	☒	The exact sample size (*n*) for each experimental group/condition, given as a discrete number and unit of measurement
☐	☒	A statement on whether measurements were taken from distinct samples or whether the same sample was measured repeatedly
☐	☒	The statistical test(s) used AND whether they are one- or two-sided<br>*Only common tests should be described solely by name; describe more complex techniques in the Methods section.*
☐	☒	A description of all covariates tested
☐	☒	A description of any assumptions or corrections, such as tests of normality and adjustment for multiple comparisons
☐	☒	A full description of the statistical parameters including central tendency (e.g. means) or other basic estimates (e.g. regression coefficient) AND variation (e.g. standard deviation) or associated estimates of uncertainty (e.g. confidence intervals)
☐	☒	For null hypothesis testing, the test statistic (e.g. F, t, r) with confidence intervals, effect sizes, degrees of freedom and P value noted<br>*Give P values as exact values whenever suitable.*
☒	☐	For Bayesian analysis, information on the choice of priors and Markov chain Monte Carlo settings
☐	☒	For hierarchical and complex designs, identification of the appropriate level for tests and full reporting of outcomes
☐	☒	Estimates of effect sizes (e.g. Cohen's *d*, Pearson's *r*), indicating how they were calculated

Our web collection on statistics for biologists contains articles on many of the points above.

Software and code

Policy information about availability of computer code

Data collection	All the data used in the present study were obtained from various publicly available sources described in the Methods section. Reformatting of the data prior to the analyses was done in the R coding environment (R version 4.0.3, 2020-10-10).
Data analysis	All analyses were conducted in the R coding environment (R version 4.0.3, 2020-10-10). The R scripts created for the analyses are available upon request to the corresponding author and are made available on corresponding author's github account.

For manuscripts utilizing custom algorithms or software that are central to the research but not yet described in published literature, software must be made available to editors and reviewers. We strongly encourage code deposition in a community repository (e.g. GitHub). See the Nature Portfolio guidelines for submitting code & software for further information.

Data

Policy information about availability of data

All manuscripts must include a data availability statement. This statement should provide the following information, where applicable:

- Accession codes, unique identifiers, or web links for publicly available datasets
- A description of any restrictions on data availability
- For clinical datasets or third party data, please ensure that the statement adheres to our policy

All the plankton data underlying the results of this paper are publicly available through OBIS (https://www.obis.org), GBIF (https://www.gbif.org), and the references cited in the extended Methods. The occurrence data used for training the species distribution models (SDMs) as well as the spatial gridded fields of plankton species diversity will be made available on PANGAEA (https://www.pangaea.de/) upon publication of the study.

Field-specific reporting

Please select the one below that is the best fit for your research. If you are not sure, read the appropriate sections before making your selection.

☐ Life sciences ☐ Behavioural & social sciences ☒ Ecological, evolutionary & environmental sciences

For a reference copy of the document with all sections, see nature.com/documents/nr-reporting-summary-flat.pdf

Ecological, evolutionary & environmental sciences study design

All studies must disclose on these points even when the disclosure is negative.

Study description	This study implements an ensemble forecasting approach based on a suite of Species Distribution Models and Earth System Models to empirically model and project the diversity (i.e. species richness and composition) of plankton communities in the surface global ocean in both contemporary and end-of-century environmental conditions.
Research sample	We compiled species occurrences for 860 plankton species (336 phytoplankton, 524 zooplankton) spanning 13 phyla, 71 orders and 324 genera. The species field occurrence records (n = 934,696) were compiled from a wide range of field surveys (e.g. oceanographic cruises) involving various sampling methodologies (continuous plankton recorder, vertical and horizontal plankton et tows, seawater bottle samples etc.). All the species-level occurrence and abundance data were aggregated onto a global monthly-resolved 1°x1° cell grid to be matched with observation-based climatologies of environmental predictors. The clades studied here correspond to the most-commonly sampled phyla of the global surface ocean.
Sampling strategy	The present study did not involve sampling of novel observations. All the data used here were retrieved from online data repositories (OBIS, https://www.obis.org; GBIF, https://www.gbif.org) that provide the largest published biogeographic information available for marine species. The phytoplankton species occurrences were retrieved from a recently published data synthesis (PhytoBase, Righetti et al., 2020). A new species occurrences synthesis was implemented for zooplankton species following the same criteria as PhytoBase.
Data collection	The plankton species occurrences were retrieved from publicly available data repositories (OBIS and GBIF) as well as a few complementary references. Similarly, the environmental predictors used to model the species environmental niches were retrieved from publicly available online repositories. The earth system models outputs used to project the species niches in the future global surface ocean were retrieved from the CMIP5 and MAREMIP initiatives data archiving portals.
Timing and spatial scale	All biological and environmental data were aggregated on a global 1°x1° monthly grid cell. This corresponded to the higher spatial-temporal resolution possible considering the variety of the data types and origins at hand.
Data exclusions	During the data filtering steps (described in the Methods section), plankton species occurrences were discarded when: 1) they were missing essential contextual metadata (sampling date, position, depth etc.); 2) they were located in coastal areas; 3) they corresponded to sediment records ; 4) they did not correspond to an organism observations identified at the species-level. Finally, the plankton species displaying too occurrences for their niches to be modelled were also excluded.
Reproducibility	This present study did not include an a priori experimental design, but all the steps of our data analyses and modelling are reproducible based on the species observations dataset that we will make available on PANGAEA upon publication and the functions embedded in the R packages we cite.
Randomization	The present study did not include an a priori experimental design, and therefore randomization was not necessary
Blinding	The present study did not include an a priori experimental design, and therefore blinding was not necessary.

Did the study involve field work? ☐ Yes ☒ No

Reporting for specific materials, systems and methods

We require information from authors about some types of materials, experimental systems and methods used in many studies. Here, indicate whether each material, system or method listed is relevant to your study. If you are not sure if a list item applies to your research, read the appropriate section before selecting a response.

Materials & experimental systems

n/a	Involved in the study
☒	☐ Antibodies
☒	☐ Eukaryotic cell lines
☒	☐ Palaeontology and archaeology
☒	☐ Animals and other organisms
☒	☐ Human research participants
☒	☐ Clinical data
☒	☐ Dual use research of concern

Methods

n/a	Involved in the study
☒	☐ ChIP-seq
☒	☐ Flow cytometry
☒	☐ MRI-based neuroimaging
